# Effects of different neuromuscular training modalities on balance performance in older adults: a systematic review and network meta-analysis

**DOI:** 10.3389/fphys.2025.1623908

**Published:** 2025-08-08

**Authors:** Yuanji Zhong, Pengwei Chen, Wenhao Guo, Yongshun Wang, Yang Xue, Penghong Chen, Jingjin Liu

**Affiliations:** ^1^ School of Physical Education and Arts, Jiangxi University of Science and Technology, Ganzhou, China; ^2^ School of Recreational Sports and Tourism, Beijing Sport University, Beijing, China; ^3^ College of Physical Education, Huaqiao University, Quanzhou, China; ^4^ School of Physical Education, Zhengzhou Normal University, Zhengzhou, China; ^5^ Department of Cardiology, Shenzhen People’s Hospital, The First Affiliated Hospital of Southern University of Science and Technology, The Second Clinical Medical College, Jinan University, Shenzhen, Guangdong, China

**Keywords:** neuromuscular training, older adults, fall prevention, balance performance, network meta-analysis

## Abstract

**Background:**

Neuromuscular training (NMT) is widely utilized to enhance balance and reduce fall risk in older adults, yet comparative effectiveness across various modalities remains unclear. This study aimed to systematically assess and rank the effects of sensorimotor training (ST), whole-body vibration training (WBVT), neurofunctional training (NT), and balance training (BT) on balance performance in older adults.

**Methods:**

A systematic review and network meta-analysis was conducted following PRISMA guidelines, including 49 randomized controlled trials with a total of 3,028 older adults. Intervention efficacy was assessed through dynamic balance (Timed Up and Go Test [TUGT], Walk Test [WT]) and static balance (Berg Balance Scale [BBS]) outcomes.

**Results:**

Significant improvements in dynamic balance (TUGT) were observed with ST (SMD = −0.92; 95% CI: −1.66, −0.18) and NT (SMD = −0.92; 95% CI: −1.44, −0.40), which ranked highest in efficacy (NT: 85.9%, ST: 83.4%). WBVT (SMD = −0.35; 95% CI: −0.69, −0.02) and BT (SMD = −0.33; 95% CI: −0.64, −0.01) also showed statistically significant, but modest effects on dynamic balance. In contrast, the effects of all interventions on static balance, as measured by the BBS, were not statistically significant, suggesting limited and inconclusive evidence regarding their impact on postural stability. Although ST ranked highest in WT (73.7%) and BT showed a favorable SUCRA value in BBS (60.2%), these rankings should be interpreted with caution.

**Conclusion:**

The corresponding effect sizes were small and not statistically significant, indicating that SUCRA reflects relative ranking probability rather than actual clinical efficacy. Therefore, the potential benefits for improving static balance and walking speed remain limited and inconclusive. ST and NT were identified as the most effective NMT modalities for significantly enhancing dynamic balance in older adults, indicating their suitability for targeted interventions in fall prevention strategies.

**Systematic Review Registration:**

https://inplasy.com/inplasy-2025-4-0015/, identifier INPLASY202540015

## 1 Introduction

The demographic shift toward global population aging has become increasingly pronounced and appears largely irreversible. Projections from the United Nations estimate that the number of individuals aged 65 and older will more than double over the next 3 decades, exceeding 1.6 billion by 2050 (United Nations, Department of Economic and Social Affairs, 2023). Within this context, falls have emerged as a significant public health issue, frequently cited as a primary contributor to disability, injury, and mortality among older adults. As age-related physiological decline and impaired neuromuscular regulation converge, the deterioration of balance function and the heightened risk of falls are becoming increasingly prominent concerns worldwide (Montero-Odasso et al., 2022; Montero-Odasso et al., 2021; World Health Organization, 2007). According to the World Health Organization (2021), falls are often accompanied by severe adverse outcomes, including fractures, traumatic brain injuries, and persistent functional limitations, all of which considerably undermine the physical independence and psychological wellbeing of older individuals (World Health Organization, 2021; Centers for Disease Control and Prevention, 2023). The economic implications are equally concerning; it has been estimated that fall-related injuries account for approximately 1.5% of total global annual healthcare expenditures. A recent Global Burden of Disease study reported that, in 2021 alone, over 548 million individuals were affected by falls, with more than 215 million incidents recorded and an estimated financial loss exceeding 43.8 million USD (Li et al., 2025). These statistics underscore the urgent need to develop effective and scalable interventions aimed at reducing fall risk, particularly in light of accelerating demographic aging.

In response to these challenges, exercise-based interventions targeting postural control and balance function in older adults have gained increasing empirical support. Aging is frequently accompanied by reductions in proprioceptive acuity, slower neural conduction velocities, and diminished muscular control—all of which can compromise balance and elevate fall risk. Against this backdrop, NMT has emerged as a promising intervention strategy, widely implemented across clinical, rehabilitative, and community-based contexts to support balance maintenance and fall prevention in aging populations (Concha-Cisternas et al., 2023).

NMT refers to a suite of training approaches designed to enhance the integrative functioning of the nervous and muscular systems, thereby improving postural regulation, sensory-motor integration, motor coordination, and rapid response capabilities. Several distinct NMT modalities have been proposed, including ST, WBVT, NT, and BT (Concha-Cisternas et al., 2023). This study focused on these four representative NMT modalities primarily because they possess well-defined physiological mechanisms, standardized intervention protocols, and relatively consistent implementation across existing literature. These characteristics help ensure conceptual homogeneity and methodological comparability, both of which are essential for valid network meta-analytic synthesis. In contrast, other widely used practices such as Tai Chi or resistance training often involve multiple overlapping mechanisms and highly variable training content and pacing, making them less suitable for inclusion within a unified neuromuscular training framework for direct comparison. Each modality engages unique physiological pathways and operates through different training principles. ST, for instance, emphasizes the enhancement of postural adaptability through the integration of proprioceptive, vestibular, and visual sensory inputs, particularly under unstable or dynamic environmental conditions (Lephart et al., 1997). WBVT delivers mechanical vibration stimuli through specialized platforms, triggering neuromuscular activation and reflexive contractions that may enhance muscle function and sensorimotor responsiveness (Liu et al., 2023). NT targets improvements in neural transmission speed and motor reactivity, seeking to optimize coordination between central and peripheral systems, particularly in situations involving postural perturbation or movement initiation (Arumugam et al., 2021). BT, in contrast, remains one of the most commonly employed and traditional modalities, utilizing structured tasks such as unipedal stance, center-of-gravity shifting, gait exercises, and balance board activities to support both static and dynamic postural stability (Sherrington et al., 2019).

Despite the broad application of these interventions, much of the existing evidence base remains fragmented. Most systematic reviews to date have tended to focus on isolated training modalities or have employed pairwise comparisons that lack the methodological capacity to evaluate the relative effectiveness across multiple NMT approaches (Sherrington et al., 2019; Liberati et al., 2009). Moreover, many studies fail to clearly delineate between static and dynamic components of balance, limiting the interpretability and clinical applicability of their findings. To address these gaps, the present study synthesizes randomized controlled trials (RCTs) examining the four primary types of NMT interventions. Through the application of network meta-analysis (NMA), the study integrates both direct and indirect evidence, evaluates training effects across a broad range of static and dynamic balance outcomes, and provides a comparative ranking of intervention efficacy (Florez et al., 2024). Unlike conventional training programs that focus predominantly on muscle strength or flexibility, NMT centers on the activation and reorganization of neuromuscular pathways, aiming to refine neuroregulatory mechanisms and optimize postural control strategies. This paradigm has gained traction across various domains, including rehabilitation, fall prevention, and functional health promotion.

The overarching aim of this review is to offer a comprehensive synthesis of high-quality RCTs investigating the effectiveness of NMT in improving balance performance among older adults. By assessing the magnitude and heterogeneity of effects across multiple training modalities and balance-related outcomes, this study seeks to identify the most efficacious interventions and explore the underlying physiological mechanisms that may account for observed differences. In doing so, it also aims to propose evidence-based, contextually adaptable strategies for stratified intervention design—ultimately contributing to a more precise and scientifically grounded approach to promoting functional health in aging populations. However, the relative efficacy of various neuromuscular training modalities in improving balance remains unclear due to fragmented evidence, and intervention choices are often based on empirical preferences rather than robust comparisons. Therefore, a systematic review and network meta-analysis is warranted to synthesize existing findings and inform evidence-based practice.

## 2 Methods

This systematic review and network meta-analysis was conducted in accordance with the guidelines outlined by the Preferred Reporting Items for Systematic Reviews and Meta-Analyses (PRISMA) statement (Hutton et al., 2015; Sterne et al., 2019). The study protocol was prospectively registered on the International Platform of Registered Systematic Review and Meta-Analysis Protocols (INPLASY), under the registration number INPLASY202540015.

### 2.1 Search strategies

A systematic and exhaustive search was performed across five leading electronic databases, including PubMed, EBSCOhost, Embase, the Cochrane Library, and Web of Science, with the aim of identifying randomized controlled trials (RCTs) that examined the effects of various NMT modalities on balance performance in older adults. Specifically, the search targeted studies evaluating ST, WBVT, NT, and BT. The search window extended from the inception of each database to 21 January 2025.

To maximize both specificity and comprehensiveness, the search strategy integrated a combination of Medical Subject Headings (MeSH) and relevant free-text keywords associated with “balance,” “falls,” “aged,” and the intervention types (Supplementary Table S1). Each database unique indexing framework was taken into account, and search syntaxes were carefully tailored to ensure optimal retrieval across platforms. In addition to the primary search, backward and forward citation tracking was performed for all included studies, enabling the identification of potentially eligible trials that may have been overlooked in the initial screening. This layered approach was designed to enhance the breadth and robustness of the evidence base.

### 2.2 Eligibility criteria

#### 2.2.1 Inclusion criteria

The inclusion criteria were formulated based on the PICOS framework, encompassing five key dimensions: population, intervention, comparison, outcomes, and study design.

Population (P): Studies were eligible if they included participants aged 60 years or older, encompassing both healthy older adults and those with mild functional impairments.

Intervention (I): Eligible studies investigated one of the NMT modalities of interest, including ST, NT (multi-strategies), WBVT, or BT. Other forms of exercise interventions such as Tai Chi, yoga, or isolated resistance training were excluded due to their diverse mechanisms and lack of alignment with standardized NMT frameworks.

Comparison (C): Control conditions included participants receiving no intervention, usual care, or alternative exercise programs. Specifically, these were classified into three categories to improve comparability: (1) passive controls (e.g., no training program), (2) usual care (e.g., conventional exercises), and (3) alternative exercise controls (e.g., Tai Chi, resistance or balance training). This classification was considered during heterogeneity assessment evaluation.

Outcomes (O): Studies were required to report objectively measured outcomes related to balance performance in older adults. Key outcome measures included dynamic balance assessments such as the TUGT and the WT, as well as static balance measures such as the BBS.

Study Design (S): Only randomized controlled trials (RCTs) were considered eligible for inclusion.

#### 2.2.2 Exclusion criteria

Studies were excluded if they met any of the following conditions: (1) Non-original or grey literature such as reviews, dissertations, conference abstracts, or technical reports, which typically lack peer-review and standardized reporting, thereby increasing the risk of bias; (2) Lack of relevant outcome indicators related to balance performance; (3) Duplicate publications or repeated analyses, in which case the most recent or highest-quality version was selected; (4) Full text was unavailable, preventing quality appraisal and data extraction; (5) Articles not published in English were excluded to ensure consistency in methodological assessment and avoid misinterpretation due to language barriers; (6) Studies that did not report both mean and standard deviation for balance outcomes, and for which the necessary data could not be extracted or obtained from the authors; (7) Non-randomized study designs; or (8) Unpublished studies were excluded, as they often lack sufficient methodological transparency and data accessibility for reliable meta-analytic synthesis.

#### 2.2.3 Study selection

The selection of studies followed the PRISMA (Preferred Reporting Items for Systematic Reviews and Meta-Analyses) guidelines and was independently performed by two reviewers [Y.Z. and P.C. (Pengwei Chen)]. Initially, duplicate records were identified and removed using EndNote X9 reference management software. The reviewers then screened the remaining articles by evaluating titles and abstracts to exclude studies that were clearly irrelevant or failed to meet the predefined inclusion criteria. For studies that appeared potentially eligible, the full texts were retrieved and examined in detail to determine final inclusion. Any discrepancies or disagreements arising during the selection process were resolved through discussion. If consensus could not be reached, a third reviewer was consulted to provide adjudication. The entire process is illustrated in a PRISMA flow diagram, which outlines the number of records identified, reasons for exclusion, and the final number of studies included in the review.

### 2.3 Data extraction

Two trained researchers [Y.Z. and P.C. (Pengwei Chen)] independently performed data extraction and quality assessment using a pre-designed Excel-based extraction form. The following information was systematically collected from each included study: (1) bibliographic details, including author name, year of publication, and country of origin; (2) participant characteristics, such as sample size, age, sex distribution, and health status; (3) details of intervention and control conditions; (4) outcome data, including means and standard deviations for relevant balance measures; and (5) information related to risk of bias assessments. Upon completion of extraction, all entries were cross-checked between the two reviewers. Any discrepancies were resolved through discussion, and if disagreement persisted, a third reviewer was consulted to reach a final decision.

### 2.4 Risk of bias

The methodological quality of the included studies was independently assessed by two reviewers [Y.Z. and P.C. (Pengwei Chen)] using the Revised Cochrane Risk-of-Bias Tool for Randomized Trials (RoB 2.0). A total of 49 randomized controlled trials (RCTs) were evaluated for risk of bias across five key domains: bias arising from the randomization process; bias due to deviations from intended interventions (including both the effect of assignment to intervention and the effect of adhering to intervention); bias due to missing outcome data; bias in measurement of the outcome; and bias in selection of the reported results (Sterne et al., 2019; Eldridge et al., 2021; Cumpston et al., 2019).

The overall risk of bias for each study was determined based on a synthesis of judgments across these five domains. A study was categorized as having a “low risk” of bias if all domains were rated as low risk. If any domain was rated as “some concerns” but none were deemed high risk, the study was classified as having “some concerns.” If one or more domains were assessed as “high risk,” the study was considered to have an overall high risk of bias. In cases where discrepancies arose between reviewers, consensus was reached through discussion. When necessary, a third reviewer was consulted to arbitrate unresolved disagreements.

### 2.5 Statistical analysis

#### 2.5.1 Network meta-analysis

All statistical analyses were performed using Stata version 17.0. A network meta-analysis (NMA) based on a random-effects model was conducted to synthesize both direct and indirect evidence across studies (Chaimani et al., 2013).

To visualize the structure of the treatment comparisons, a frequency-based network plot was constructed. In this diagram, each node represents a different intervention, and the size of the node is proportional to the total sample size within that intervention group. Lines connecting the nodes indicate the presence of direct comparisons between interventions; the thickness of each line reflects the number of trials contributing to that comparison. The absence of a connecting line between two nodes indicates that no direct head-to-head trial exists, and the relative effect is estimated through indirect evidence derived from the network structure (Chaimani et al., 2024; Chaimani et al., 2013).

The analysis assumed a common between-study variance (τ^2^) across all comparisons. To preliminarily assess heterogeneity, pairwise meta-analyses were first conducted using the I^2^ statistic and corresponding p-values. When the measurement units for outcome indicators differed across studies, standardized mean differences (SMDs) were used as the summary effect size. When measurement units were consistent, weighted mean differences (WMDs) were applied. The degree of heterogeneity was evaluated using the Q test and I^2^ values; fixed-effects models were adopted when heterogeneity was low (p < 0.1 and I^2^ < 50%), while random-effects models were used in the presence of substantial heterogeneity (p > 0.1 and I^2^ > 50%) (Higgins et al., 2024; Deeks et al., 2024).

In cases where significant heterogeneity was identified in specific comparisons (e.g., I^2^ > 50% or p < 0.1), studies contributing high heterogeneity or classified as high risk of bias according to RoB 2.0 were excluded in sensitivity analyses. Additionally, changes in the Surface Under the Cumulative Ranking curve (SUCRA) values were examined to assess the robustness of the effect estimates.

To examine the consistency between direct and indirect evidence, both global and local inconsistency assessments were conducted. For loops within the network where multiple comparisons formed a closed circuit, inconsistency factors (IFs) and their 95% confidence intervals were calculated. An IF whose confidence interval includes zero suggests consistency between direct and indirect comparisons; otherwise, the presence of inconsistency is suspected. In cases where inconsistency was detected, potential sources such as differences in intervention protocols, sample characteristics, or measurement variability were explored. If necessary, inconsistency models were considered, or the reporting was limited to direct comparisons.

The comparative effects of different interventions were presented using a Netleague table and corresponding triangular plots, which reported SMDs along with their 95% confidence intervals. Interpretation of effect sizes was based on the magnitude of the SMD: values less than 0.2 were considered negligible, 0.2 to 0.5 indicated small effects, 0.5 to 0.8 represented moderate effects, and values greater than or equal to 0.8 were classified as large effects. An SMD whose confidence interval crossed zero was considered statistically non-significant, whereas a confidence interval that did not include zero indicated a statistically significant difference.

To further rank the comparative efficacy of the interventions, SUCRA values were calculated for each treatment. These values range from 0% to 100%, with higher percentages indicating greater likelihood of being the most effective intervention. A SUCRA value of 100% reflects the highest possible rank, suggesting that the intervention is most likely to be the optimal choice, whereas a value of 0% indicates the lowest probability of benefit. This ranking framework enabled a quantitative comparison of the relative performance of the various NMT modalities in improving balance among older adults.

#### 2.5.2 Heterogeneity assessment

After obtaining the pooled effects from the NMA, we quantified between-study variability for each outcome (TUGT, WT, BBS) using the Cochran Q statistic, the I^2^ inconsistency index, and the between-study variance (τ^2^). We also generated Galbraith (radial) plots to visually detect high-leverage outliers and potential dispersion patterns. I^2^ values were interpreted as low (<25%), moderate (25%–75%), or high (>75%) heterogeneity. If I^2^ > 50% or Q-test p < 0.10, heterogeneity was considered substantial and prompted predefined subgroup and sensitivity analyses.

#### 2.5.3 Subgroup analyses

To identify potential sources of heterogeneity, subgroup analyses were conducted based on three predefined stratification variables: (1) type of intervention (ST, WBVT, NT, BT), (2) age group (<70, 70–80, >80 years), and (3) health status (healthy, osteoporosis, stroke, and other conditions).

For each subgroup, the pooled effect size was calculated using Hedges’ g, and between-subgroup differences were tested using Cochran’s Q between statistic. A threshold of p < 0.05 was used to indicate a statistically significant difference between subgroups, suggesting that the corresponding variable may account for a meaningful portion of the observed heterogeneity.

#### 2.5.4 Sensitivity analysis

To assess the robustness of the pooled estimates and intervention rankings, a leave-one-out sensitivity analysis was performed. In this procedure, each included study was iteratively removed, and the summary effect sizes and SUCRA values were recalculated. If the exclusion of any single study led to a change in the pooled estimate or SUCRA exceeding 10% of the original value, the result was considered sensitive to that study, indicating a potentially influential effect on the overall findings.

#### 2.5.5 Assessment of publication bias

To evaluate the potential risk of publication bias, funnel plots were constructed for each outcome using Stata version 17.0. Visual inspection of these plots was employed to assess asymmetry, which may indicate potential small-study effects or selective reporting (Page et al., 2024). In addition to visual methods, Egger’s linear regression test was conducted to formally test for funnel plot asymmetry. A statistically significant intercept (p < 0.05) was considered indicative of possible publication bias. This approach provides a quantitative complement to visual assessments, particularly in meta-analyses involving ten or more studies per outcome.

All statistical procedures and interpretations were independently conducted and cross-validated by two reviewers [Y.Z. and P.C. (Pengwei Chen)] to ensure analytic accuracy and methodological rigor.

## 3 Results

### 3.1 Study selection

**FIGURE 1:**
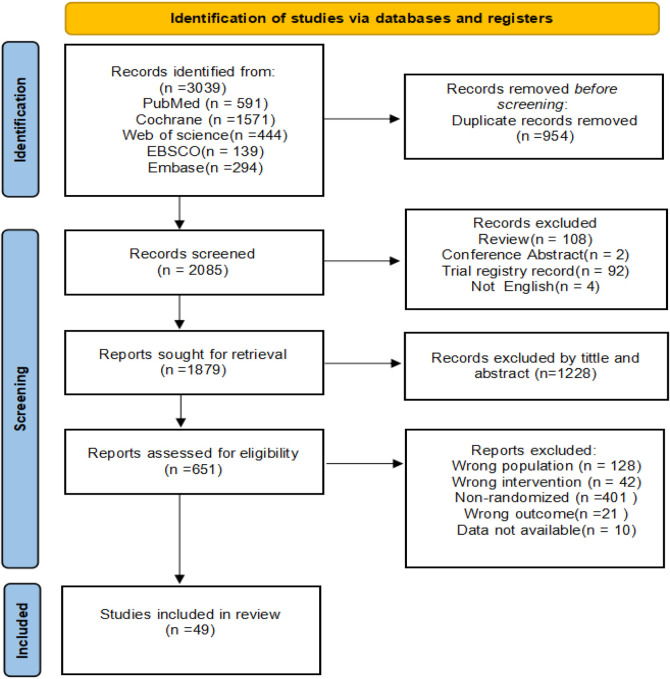
Summary PRISMA (Preferred reporting items for systematic reviews and network meta-analyses) flowchart identifying the study selection process.

A total of 3,039 records were initially retrieved through systematic database searching. Two reviewers independently screened the titles and abstracts based on the predefined inclusion and exclusion criteria, followed by a full-text review to assess eligibility. After removing duplicates and excluding irrelevant or ineligible studies, 49 randomized controlled trials (RCTs) met the inclusion criteria and were ultimately included in the meta-analysis (Karaca and Kılınç, 2024; Shabir et al., 2021; Jimenez-Mazuelas et al., 2024; Espejo-Antúnez et al., 2020; Sinaki and Lynn, 2002; Stolzenberg et al., 2013; Tseng et al., 2016a; Tseng et al., 2016b; Sievänen et al., 2024; Bautmans et al., 2005; Bogaerts et al., 2007; Nawrat-Szołtysik et al., 2022; Lam et al., 2018; Goudarzian et al., 2017; Bogaerts et al., 2011; Zhang et al., 2014; Pollock et al., 2012; Asahina et al., 2023; Ko et al., 2017; Yang et al., 2023; Kang and Park, 2024; Zarzeczny et al., 2024; Jang and Park, 2021; Mesquita et al., 2015; Concha-Cisternas et al., 2024; Smaili et al., 2018; Acheche et al., 2020; Yuzlu et al., 2022; Rossi et al., 2014; Halvarsson et al., 2015; An et al., 2024; Steadman et al., 2003; Hernández-Guillén et al., 2020; Lee et al., 2012; Hirase et al., 2015; El-Khoury et al., 2015; Mikó et al., 2018; Madureira et al., 2007; Madureira et al., 2010; Markovic et al., 2015; Wallén et al., 2018; Bao et al., 2018; Ni et al., 2014; Santos et al., 2017; Carter et al., 2001; Sadeghi et al., 2021; Sörlén et al., 2021; Donath et al., 2016; Allin et al., 2020). The detailed selection process is illustrated in the PRISMA flow diagram (Figure 1).

### 3.2 Study characteristics

This systematic review and network meta-analysis included a total of 49 randomized controlled trials, comprising 3,028 older adult participants. The studies were published between 2001 and 2024 and were geographically diverse, representing 20 countries and regions including China, the United States, the United Kingdom, France, Germany, Canada, Japan, South Korea, Pakistan, Spain, Belgium, Poland, Iran, Brazil, Chile, Tunisia, Turkey, Sweden, Hungary, and Croatia (Table 1).

**TABLE 1:** Characteristics of the included randomized controlled trials.

Study	Population	Research object			Intervention measure				Outcome
		Gender	Type	Age	Intervention	Control	Duration	Frequency	
Karaca O and Kılınç M (2024) Türkiye	IG: 11 CG: 14	M/F	Stroke patients	IG/CG: 63–66	ST	Trunk-centered Bobath exercises	8 weeks	Three times a week; 40 min	BBS↓ 2 m-WT↑
Shabir et al. (2021) Pakistan	IG: 20 CG: 20	F	Postmenopause obesity	IG/CG: 50.27 ± 3.17	ST	Conventional therapy	8 weeks	Three times a week; 35–40 min	FRT↑ TUGT↑ OLST↑
Jimenez-Mazuelas et al. (2024) Spain	IG: 15 CG: 15	M/F	Diabetic	IG/CG: 70.0 ± 8.0	ST	Conventional exercises	3 months	Eight times a month; 60 min	FSST↓ TUGT↑ FES↑
Espejo-Antúnez L et al. (2020) Spain	IG: 21 CG: 21	M/F	N/A	IG: 83.21 ± 6.59 CG: 82.72 ± 6.40	ST	Conventional therapy	12 weeks	Twice a week; 55 min	TUGT↑ CPT↑ TS↑ OLST↑
Sinaki and Lynn (2002) United States	IG: 2 CG: 3	F	Osteoporotic	IG/CG: 70–83	ST	Exercise therapy	4 weeks	Every day	CDP↓
Stolzenberg et al. (2013) Germany	IG: 26 CG: 31	F	Post-menopausal osteoporosis	IG: 65.9 ± 4.5 CG: 67.3 ± 3.7	WBVT	BT	9 months	Twice a week; 5 min	RST↓ STST↓ TST↓ OLST↓
Tseng et al. (2016a) China	IG: 15 CG: 15	M/F	N/A	IG/CG: 69.6 ± 3.9	WBVT	No training program	3 months	Three times a week; 5 min	LOST↑ SRT↑
Tseng et al. (2016b) China	IG: 14 CG: 14	M/F	N/A	IG/CG: 69.22 ± 3.97	WBVT	No training program	3 months	Three times a week; 5 min	LOST↑
Sievänen et al. (2024) United Kingdom	IG: 63 CG: 58	M/F	N/A	IG: 77.8 ± 6.8 CG: 79.2 ± 7.6	WBVT	Wellness training	10 weeks	Twice a week; 3–5 min	SPPB↑ TUGT↑ 5t-CST↑ GT↑
Bautmans et al. (2005) Belgium	IG: 10 CG: 11	M/F	N/A	IG/CG: 77.5 ± 11.0	WBVT	BT	6 weeks	Three times a week; 1–5 min	TUGT↓ GT↓ 30s-CST↓
Bogaerts et al. (2007) Belgium	IG: 94 CG: 66	M/F	N/A	IG: 66.8 ± 0.55 CG: 67.9 ± 0.68	WBVT	Fitness	12 months	Three times a week; 5–10 min	SOT↓
Nawrat-Szołtysik et al. (2022) Poland	IG: 22 CG: 20	F	N/A	IG: 69.0 ± 6.74 CG: 70.7 ± 6.96	WBVT	No training program	12 weeks	Twice a week; 10 min	TUGT↑ 6 m-WT↑ 30s-CST↑ FES-I↓
Lam et al. (2018) China	IG1: 25 IG2: 23 CG: 24	M/F	N/A	IG1: 84.0 ± 6.7 IG2: 82.4 ± 7.6 CG: 80.3 ± 7.3	IG1: WBVT IG2: BT	Conventional exercises	8 weeks	Three times a week; 1–15 min	TUGT↓ BBS↑ FTSST↑ 6 m-WT↓
Goudarzian et al. (2017) Iran	IG: 7 CG: 7	F	N/A	IG: 66 ± 4.58 CG: 68 ± 9.20	WBVT + Placebo	No training program	10 days	Every day; 30 min	FBT↓ TUGT↑ GT↓ 30 m-WT↑
Bogaerts et al. (2011) Belgium	IG: 50 CG: 53	F	N/A	IG: 79.8 ± 5.3 CG: 79.6 ± 5.2	WBVT	No training program	6 months	Three times a week; 1–15 min	SOT↓ 10 m-WT↑ TUGT↑
Zhang et al. (2014) China	IG: 19 CG: 18	M/F	N/A	IG: 85.8 ± 3.58 CG: 84.6 ± 3.68	WBVT	Conventional exercises	8 weeks	Three to five times a week; 4–5 min	TUGT↑ 30s-CST↑ ABC↑
Pollock et al. (2012) United Kingdom	IG: 24 CG: 32	M/F	N/A	IG: 80.0 ± 1.4 CG: 82.2 ± 1.3	WBVT	Exercise	8 weeks	Three times a week; 5 min	TUGT↑ 6 m-WT↑ BBS↓ FES-I↑
Asahina et al. (2023) Japan	IG: 42 CG: 46	M	Hemodialysis patients	IG: 75.0 ± 6.0 CG: 77.0 ± 7.0	WBVT	No training program	12 weeks	Three times a week; 3 min	TUGT↓ OLST↓ 30s-CST↓
Ko et al. (2017) China	IG: 9 CG: 10	M/F	N/A	IG/CG: ≥60	WBVT	No training program	8 weeks	Three times a week; 5 min	LOST↑ STS↑
Yang et al. (2023) United States	IG: 22 CG: 20	M/F	N/A	IG/CG: ≥65	WBVT	No training program	8 weeks	Three times a week; 5 min	BBS↑ CRT↓
Kang and Park (2024) Korea	IG: 11 CG: 11	F	N/A	IG: 74.9 ± 6.33 CG: 74.2 ± 7.92	NT	Traditional training	12 weeks	Twice a week; 40 min	TUGT↑ YBT↑ RST↑
Zarzeczny et al. (2024) Poland	IG: 10 CG: 9	F	N/A	IG: 83.4 ± 6.5 CG: 83.8 ± 4.4	NT	Traditional training	12 weeks	Three times a week; 30 min	TUGT↑ 30s-CST↑ 6 m-WT↑
Jang and Park (2021) Korea	IG: 8 CG: 9	F	N/A	IG: 73.2 ± 4.76 CG: 71.8 ± 6.69	NT	No training program	4 weeks	Three to four times a week; 40 min	FTSST↑ TUGT↑ OLST↑ YBT↑
Mesquita et al. (2015) Brazil	IG: 20 CG: 18	F	N/A	IG: 68.5 ± 5.4 CG: 71.5 ± 6.2	NT	No training program	4 weeks	Three times a week; 50 min	TUGT↑ FRT↑ BBS↑
Concha-Cisternas et al. (2024) Chile	IG1: 16 IG2: 16 CG: 16	F	N/A	IG/CG: 60–80	IG1: NT IG2: BT	No training program	12 weeks	Twice a week; 30 min	SPPB↑ 6 m-WT↑ GT↑ FTSST↑
Smaili et al. (2018) Brazil	IG: 12 CG: 14	M/F	Parkinson	IG: ≥60	NT	RT	12 weeks	Twice a week; 60 min	5 m-WT↓ GT↓
Acheche et al. (2020) Tunisia	IG: 22 CG: 20	M	Chronic	IG: 63.0 ± 4.0 CG: 62.0 ± 6.0	NT	RT	24 weeks	Three times a week; 85 min	TUGT↑ 6 m-WT↑ BBS↑
Yuzlu et al. (2022) Turkey	IG: 25 CG: 29	M/F	N/A	IG: 82.9 ± 6.6 CG: 85.3 ± 7.2	BT	Conventional exercises	8 weeks	Twice a week; 10 min	BBS↑ TUGT↑ 10 m-WT↑ FES↑
Rossi et al. (2014) Brazil	IG: 23 CG: 23	F	N/A	IG: 67.0 ± 2.0 CG: 67.9 ± 3.1	BT	No training program	6 weeks	Three times a week; 30 min	TUGT↑
Halvarsson et al. (2015) Sweden	IG: 25 CG: 26	M/F	Osteoporosis	IG/CG: 66–87	BT	No training program	12 weeks	Three times a week; 45 min	FES↑ GT↑ OLST↑
An et al. (2024) Korea	IG: 21 CG: 21	F	Total knee arthroplasty	IG: 73.6 ± 4.8 CG: 72.3 ± 4.6	BT	Conventional exercises	4 weeks	Five times a week; 30 min	TUGT↑ 10 m-WT↑
Steadman et al. (2003) United Kingdom	IG: 82 CG: 84	M/F	N/A	IG/CG: 82.7 ± 5.6	BT	Conventional exercises	6 weeks	Twice a week; 45 min	BBS↑ 10 m-WT↑
Hernández-Guillén et al. (2020) Spain	IG: 14 CG: 14	M/F	N/A	IG: 76.8 ± 6.8 CG: 72.3 ± 6.8	BT	Exercises	4 weeks	Twice a week; 40 min	BBS↑
Lee et al. (2012) Korea	IG: 17 CG: 18	M/F	N/A	IG: 72.7 ± 5.10 CG: 74.3 ± 3.97	BT	No training program	4 weeks	Twice a week; 60 min	TUGT↓ FRT↓ OLST↓
Hirase et al. (2015) Japan	IG: 29 CG: 28	M/F	N/A	IG: 82.0 ± 5.7 CG: 82.2 ± 6.3	BT	No training program	4 months	A 60-min weekly	OLST↑ CST↑ TUGT↑ TST↑ FES↑
El-Khoury et al. (2015) France	IG: 306 CG: 294	F	Risk women aged	IG: 79.8 ± 2.8 CG: 79.6 ± 2.8	BT	No training program	2 years	Once a week; 60 min	TUGT↓ 6 m-WT↑ FTSST↑ OLST↑ FES-I↑
Mikó et al. (2018) United Kingdom	IG: 49 CG: 48	F	Osteoporosis	IG: 69.3 ± 4.56 CG: 69.1 ± 5.30	BT	Regular walking	12 months	Three times a week; 30 min	TUGT↑ BBS↑ RBT↑
Madureira et al. (2007) Brazil	IG: 34 CG: 32	F	Osteoporosis	IG: 74.5 ± 4.82 CG: 73.4 ± 4.61	BT	No training program	12 months	Once a week; 60 min	BBS↑ TUGT↑
Madureira et al. (2010) Brazil	IG: 30 CG: 30	F	Osteoporosis	IG/CG: ≥65	BT	No training program	12 months	Once times a week; 60 min	BBS↑
Markovic et al. (2015) Croatia	IG: 16 CG: 14	F	N/A	IG/CG: 70.0 ± 4.0	BT	PT	8 weeks	Three times a week;	SB-COP↓
Wallén et al. (2018) Sweden	IG: 47 CG: 44	M/F	Parkinson	IG: 73.1 ± 5.8 CG: 73.0 ± 5.5	BT	Conventional care	10 weeks	Three times a weeks; 60 min	GT↑ Mini-BESTest↑
Bao et al. (2018) United States	IG: 6 CG: 6	M/F	N/A	IG: 76.2 ± 5.5 CG: 75.0 ± 4.7	BT	No training program	8 weeks	Three times a weeks; 45 min	ABC↓ SOT↑ Mini-BESTest↑ FTSST↑ FSST↑ FRT↑ GT↓ TUGT↑
Ni et al. (2014) United States	IG: 15 CG: 11	M/F	N/A	IG: 77.8 ± 7.78 CG: 70.3 ± 5.69	BT	Tai Chi	12 weeks	Twice a week; 50 min	8UG↑ OLST↑ FRT↑ COP↑
Santos et al. (2017) Brazi	IG: 12 CG: 14	M/F	Parkinson	IG: 68.5 ± 6.5 CG: 67.0 ± 7.9	BT	RT	8 weeks	Twice a week; 60 min	RBT↓ OLST↑ BESTest↑
Carter et al. (2001) Canada	IG: 40 CG: 39	F	Osteoporosis	IG/CG: 65–70	BT	No training program	10 weeks	Twice a week; 60 min	SB↓ DB↓ TFE Run↓
Sadeghi et al. (2021) Iran	IG: 14 CG: 15	M	N/A	IG/CG: 71.8 ± 6.09	BT	Daily activities	8 weeks	Three times a weeks; 40 min	OLST↑ TST↑ TUGT↑ 10MWT↑
Sörlén et al. (2021) Sweden	IG: 22 CG: 29	M/F	N/A	IG/CG: ≥70	BT	No training program	4 weeks	Three times a weeks; 15 min	TUGT↓ FES↓ FES-I↓
Donath et al. (2016) Switzerland	IG: 17 CG: 15	M/F	N/A	IG: 69.1 ± 5.8 CG: 69.2 ± 6.1	BT	Regular daily activity	8 weeks	Twice a week; 50 min	OLST↑ YBT↑
Allin et al. (2020) United States	IG: 20 CG: 14	M/F	N/A	IG/CG: 71–75	BT	No training program	2 weeks	Twice a week; 30–60 min	GT↑

Abbreviations: CG, control group; IG, intervention group; M, male; F, female; ST, sensorimotor training; WBVT, Whole-Body Vibration Training; NT, neurofunctional training; BT, balance training; RT, resistance training; PT, pilates training; TIS, trunk impairment scale; SB, static balance; DB, dynamic balance; COP, center of pressure; WT, walk test; OLST, one leg stance test; TUGT, timed up and go test; BBS, berg balance scale; FES, falls efficacy scale; FES-I, falls efficacy scale international; CST, chair stand test; CRT, Chair-Rise Test; STS, Sit-To-Stand; FRT, functional reach test; FSST, four square step test; GT, gait test; SOT, sensory organization test; CPT, cooper test; TS, tinetti scale; RST, Romberg-Stance Test; STST, Semi-Tandem-Stance Test; TST, Tandem-Stance Test; LOST, limits of stability test; SRT, sit and reach test; SPPB, short physical performance battery; FTSST, Five-Times Sit-to-Stand Test; FBT, flamingo balance test; ABC, Activities-specific Balance Confidence Scale; YBT, Y-Balance Test; Mini-BESTest, Mini-Balance Evaluation System Test; 8UG, 8-Foot Up and Go; CDP, computerized dynamic post urography; BESTest, Balance Evaluation Systems Test; TFE, Run, Timed Figure of Eight Run Test; RBT, romberg test; POMA, Tinetti-Performance-Oriented Mobility Assessment; ↑, Significant Between-Group Improvement; ↓, Non-Significant Between-Group.

Participant Characteristics: All included participants were aged 60 years or older. Among the studies, 11 focused on participants aged 50–65, 28 on those aged 65–80, and 10 on individuals over 80 years of age. While the majority of participants were functionally independent older adults, a subset of the studies included individuals with specific conditions such as osteoporosis (n = 7), Parkinson’s disease (n = 3), postoperative recovery (n = 2), stroke (n = 2), and diabetes (n = 1). Importantly, all participants retained the capacity to engage in structured exercise interventions. Female participants constituted the majority of the sample, although some studies included either male-only or mixed-gender samples.

Intervention Characteristics: The experimental groups received one of four NMT modalities: (ST, n = 5), (WBVT, n = 15), (NT, n = 7), or (BT, n = 22). Control groups varied across studies: 22 employed no intervention, 18 used standard care or routine exercise protocols, and nine applied alternative interventions such as resistance training, Pilates, Tai Chi, or aquatic therapy. In several studies, BT was also utilized as a background intervention or a low-intensity comparator for control groups.

Training Protocols: The duration of interventions ranged from 4 weeks to 24 months, with session frequency varying from once to five times per week. Session durations ranged from 5 to 85 min. Overall, most studies implemented moderate-intensity programs lasting 8–12 weeks, with training typically conducted 2 to 3 times per week for 30–60 min per session.

Outcome Measures: A wide array of validated assessment tools was employed to evaluate balance performance. Dynamic balance was most commonly measured using the (TUGT, n = 28) and the (WT, n = 13), while static balance was primarily assessed using the (BBS, n = 12). Additionally, more than 30 other outcome indicators were reported across studies, including but not limited to the Functional Reach Test (FRT), Short Physical Performance Battery (SPPB), Falls Efficacy Scale (FES), FES-International (FES-I), Four Square Step Test (FSST), Gait Test (GT), Activities-specific Balance Confidence Scale (ABC), Y-Balance Test (YBT), Mini-Balance Evaluation Systems Test (Mini-BESTest) and Balance Evaluation Systems Test (BESTest) et al. Collectively, these instruments captured the multidimensional nature of balance function in older adults.

### 3.3 Risk of bias assessment results

**FIGURE 2:**
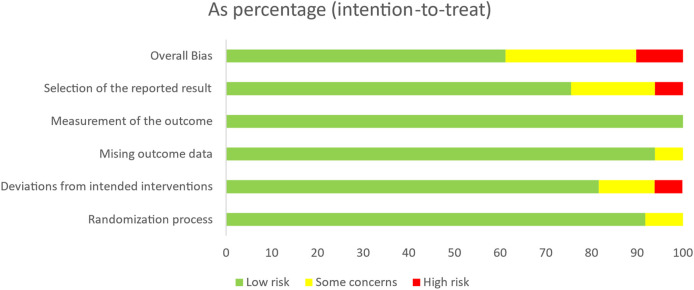
Risk of overall bias.

Figure 2 and Supplementary Figure S1 present the overall and domain-specific results of the risk of bias assessment using the RoB 2.0 tool across the 49 included randomized controlled trials. Of these, 30 studies (61.2%) were judged to have a low risk of bias, 13 studies (26.5%) were rated as having some concerns, and six studies (12.2%) were assessed as high risk. These findings suggest that the majority of the included trials demonstrated strong internal validity, although a small proportion exhibited potential sources of methodological or reporting bias. All 49 studies followed a randomized controlled design, with documented allocation concealment procedures reported in every case. Four studies (Sinaki and Lynn, 2002; Stolzenberg et al., 2013; Bogaerts et al., 2007; Lee et al., 2012) presented baseline differences between groups; however, these differences were not deemed to materially affect the interpretation of outcomes. In 45 of the included trials, dropout rates were reported as below 20%, and participant retention was adequate. Although most studies did not implement blinding of participants or assessors, the risk of performance and detection bias was considered low because the outcomes, including the TUGT, WT, and BBS, are objective measures used to quantitatively assess balance performance.

In terms of selective reporting and other potential sources of bias, a substantial number of studies lacked sufficient detail to permit a definitive judgment, and were therefore rated as having some concerns in these domains. Overall, the included studies were judged to have a generally low risk of bias, providing a solid methodological foundation for the validity the network meta-analysis results. In subsequent analyses, we further assessed the influence of high-risk studies on pooled effect estimates through sensitivity analyses.

### 3.4 Network meta-analysis

#### 3.4.1 Timed up and go test

Among the 28 studies that reported TUGT outcomes, three studies (Madureira et al., 2007; Madureira et al., 2010; Sadeghi et al., 2021) were identified as having potential risk of bias based on the RoB 2.0 assessment (Supplementary Figure S5). Given their methodological limitations and disproportionate influence on overall heterogeneity, these studies were excluded in the sensitivity analysis. Their removal did not substantially alter the pooled effect estimates or SUCRA rankings, confirming the robustness of the primary findings. Consequently, 25 high-quality randomized controlled trials (Shabir et al., 2021; Espejo-Antúnez et al., 2020; Sievänen et al., 2024; Bautmans et al., 2005; Nawrat-Szołtysik et al., 2022; Lam et al., 2018; Goudarzian et al., 2017; Bogaerts et al., 2011; Zhang et al., 2014; Pollock et al., 2012; Asahina et al., 2023; Kang and Park, 2024; Zarzeczny et al., 2024; Jang and Park, 2021; Mesquita et al., 2015; Acheche et al., 2020; Yuzlu et al., 2022; Rossi et al., 2014; An et al., 2024; Lee et al., 2012; Hirase et al., 2015; El-Khoury et al., 2015; Mikó et al., 2018; Bao et al., 2018; Sörlén et al., 2021) involving a total of 1,770 older participants were included in the TUGT outcome analysis.

**FIGURE 3:**
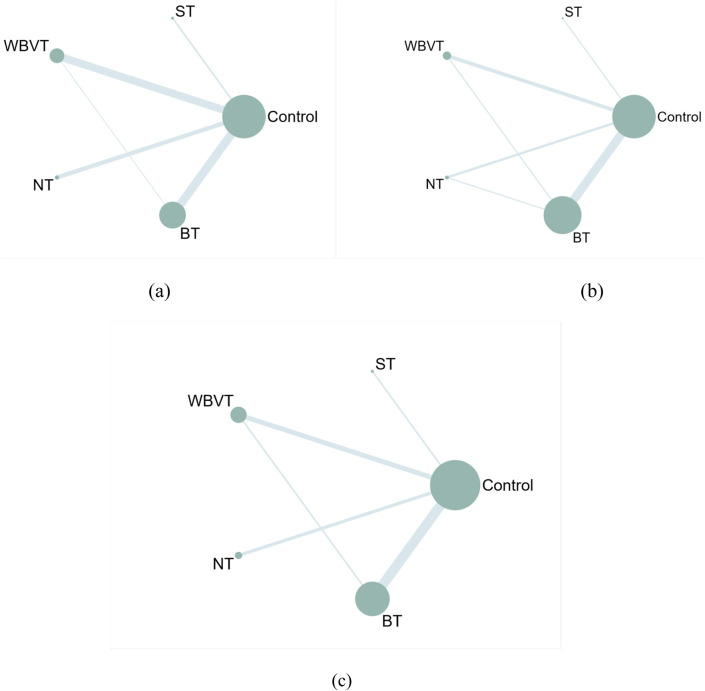
Network evidence diagram for **(a)** TUGT, **(b)** WT, **(c)** BBS.

As shown in Figure 3a, the network plot illustrates the direct comparisons between intervention modalities. The largest number of direct comparisons was observed between BT and WBVT, whereas ST had fewer direct comparisons with other modalities. A closed-loop structure was formed among BT, WBVT, and the control condition. According to the inconsistency analysis (Supplementary Table S2), the 95% confidence interval for the inconsistency factor (IF) within this loop contained zero, indicating that there was no significant inconsistency between direct and indirect evidence.

The results of the network meta-analysis, as presented in the Netleague table (Table 2) and the predictive interval plot (Supplementary Figure S2), showed that compared to the control group, all four interventions significantly improved dynamic balance performance.

**TABLE 2:** Netleague table for NMA.

TUGT				
ST	0.57 (−0.25, 1.38)	−0.00 (−0.91, 0.91)	0.59 (−0.22, 1.40)	0.92 (0.18, 1.66)
−0.57 (−1.38, 0.25)	WBVT	−0.57 (−1.19, 0.06)	0.02 (−0.42, 0.47)	0.35 (0.02, 0.69)
0.00 (−0.91, 0.91)	0.57 (−0.06, 1.19)	NT	0.59 (−0.02, 1.20)	0.92 (0.40, 1.44)
−0.59 (−1.40, 0.22)	−0.02 (−0.47, 0.42)	−0.59 (−1.20, 0.02)	BT	0.33 (0.01, 0.64)
**−0.92 (−1.66, −0.18)**	**−0.35 (−0.69, −0.02)**	**−0.92 (−1.44, −0.40)**	**−0.33 (−0.64,** −**0.01)**	Control

WT				
ST	0.26 (−0.69, 1.21)	0.57 (−0.48, 1.62)	0.29 (−0.61, 1.19)	0.19 (−0.68, 1.06)
−0.26 (−1.21, 0.69)	WBVT	0.31 (−0.38, 1.00)	0.03 (−0.38, 0.44)	−0.07 (−0.45, 0.31)
−0.57 (−1.62, 0.48)	−0.31 (−1.00, 0.38)	NT	−0.28 (−0.87, 0.31)	−0.38 (−0.96, 0.19)
−0.29 (−1.19, 0.61)	−0.03 (−0.44, 0.38)	0.28 (−0.31, 0.87)	BT	−0.10(−0.32, 0.12)
−0.19 (−1.06, 0.68)	0.07 (−0.31, 0.45)	0.38 (−0.19, 0.96)	0.10 (−0.12, 0.32)	Control

BBS				
ST	0.62 (−0.77, 2.00)	−0.01 (−1.50, 1.47)	0.40 (−0.91, 1.72)	0.05 (−1.19, 1.29)
−0.62 (−2.00, 0.77)	WBVT	−0.63 (−1.65, 0.40)	−0.21 (−0.92, 0.49)	−0.57 (−1.18, 0.05)
0.01 (−1.47, 1.50)	0.63 (−0.40, 1.65)	NT	0.41 (−0.52, 1.35)	0.06 (−0.75, 0.88)
−0.40 (−1.72, 0.91)	0.21 (−0.49, 0.92)	−0.41 (−1.35, 0.52)	BT	−0.35 (−0.80, 0.10)
−0.05 (−1.29, 1.19)	0.57 (−0.05, 1.18)	−0.06 (−0.88, 0.75)	0.35 (−0.10, 0.80)	Control

Note: Values in the table represent standardized mean differences (SMDs) with 95% confidence intervals for TUGT, WT, and BBS outcomes. SMDs are unitless indices used to compare intervention effects across studies with different measurement scales. Effect sizes were interpreted as negligible (<0.2), small (0.2–0.5), moderate (0.5–0.8), or large (≥0.8). Confidence intervals crossing zero indicate non-significant results. Corresponding raw values (mean ± SD) are provided in Supplementary File 1 for reference. Bolded values indicate statistically significant between-group differences.

Specifically, ST (SMD = −0.92; 95% CI: −1.66 to −0.18), WBVT (SMD = −0.35; 95% CI: −0.69 to −0.02), NT (SMD = −0.92; 95% CI: −1.44 to −0.40), and BT (SMD = −0.33; 95% CI: −0.64 to −0.01) all demonstrated statistically significant effects.

Although the WBVT group exhibited a slightly greater reduction in TUGT times compared to the BT group (SMD = −0.02; 95% CI: −0.47 to 0.42), this difference was not statistically significant. Similarly, while both NT and ST showed significant improvements over the control group, their direct comparison (SMD = 0.00; 95% CI: −0.91 to 0.91) revealed no significant difference, suggesting comparable effectiveness between the two interventions in enhancing dynamic balance among older adults. While SUCRA rankings aid in visualizing relative efficacy, they reflect probability, not effect magnitude or significance. For example, although ST ranked highest for WT (SUCRA = 73.7%), its effect was nonsignificant (SMD = 0.13; 95% CI: −0.10 to 0.36). Thus, SUCRA should be interpreted alongside effect sizes and confidence intervals to avoid overgeneralization.

**FIGURE 4:**
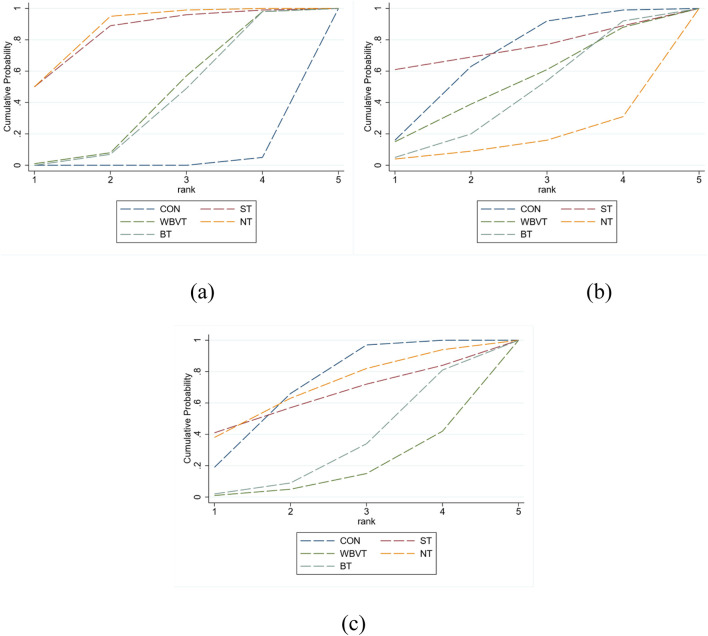
Cumulative ranking probability plots (SUCRA) for **(a)** TUGT, **(b)** WT, and **(c)** BBS.

As illustrated by the SUCRA distribution curves (Figure 4a), the cumulative ranking probabilities for improving dynamic balance were as follows: NT (85.9%), ST (83.4%), WBVT (41.0%), BT (38.5%), and control (1.2%). These findings suggest that NT and ST are potentially the most effective NMT modalities for improving dynamic balance in older populations.

From a clinical perspective, both ST and NT demonstrated large effect sizes on TUGT performance (SMD = −0.92), exceeding the conventional threshold for a large effect (|SMD| ≥ 0.8). This indicates not only statistical significance but also meaningful functional improvement that may be observable in real-world settings.

#### 3.4.2 Walk test

Among the 13 studies reporting WT outcomes, two studies (Pollock et al., 2012; Sadeghi et al., 2021) were excluded from the final analysis due to concerns related to methodological quality based on RoB 2.0 assessments and notable discrepancies in outcome measurement units, which may have compromised the consistency and reliability of pooled estimates (Supplementary Figure S8). Consequently, 11 high-quality randomized controlled trials (Karaca and Kılınç, 2024; Lam et al., 2018; Goudarzian et al., 2017; Bogaerts et al., 2011; Zarzeczny et al., 2024; Concha-Cisternas et al., 2024; Acheche et al., 2020; Yuzlu et al., 2022; An et al., 2024; Steadman et al., 2003; El-Khoury et al., 2015), involving a total of 1,186 older adults, were included in the WT analysis.

As illustrated in Figure 3b, the network plot displays the direct comparisons among the intervention modalities. The most frequent direct comparisons were between BT and WBVT, while ST had comparatively fewer direct links with other interventions. Notably, two closed-loop structures were observed in the network: BT–WBVT–Control and BT–NT–Control. The loop inconsistency test results in Supplementary Table S2 indicate that the 95% confidence intervals of the inconsistency factors (IFs) included zero in all cases, suggesting a high level of consistency between direct and indirect evidence within the network.

The Netleague table (Table 2) and the corresponding predictive interval plot (Supplementary Figure S3) showed the following effect estimates [SMD (95% CI)] for each intervention compared to the control group: ST (SMD = −0.19; 95% CI: −1.06 to 0.68), WBVT (SMD = 0.07; 95% CI: −0.31 to 0.45), NT (SMD = 0.38; 95% CI: −0.19 to 0.96), and BT (SMD = 0.10; 95% CI: −0.12 to 0.32). All confidence intervals crossed the null value, indicating that none of the interventions demonstrated a statistically significant improvement in dynamic balance, as measured by WT, relative to the control group.

The SUCRA-based cumulative ranking for WT (Figure 4b) indicated that ST (73.7%) ranked highest among all interventions, followed by Control (67.7%), WBVT (50.6%), BT (42.7%), and NT (15.3%). However, this ranking should be interpreted with caution.Although ST achieved the highest SUCRA score, the corresponding effect was small and failed to reach statistical significance, indicating limited clinical relevance. Notably, the Control group ranked second, suggesting that none of the interventions produced consistently superior outcomes in walking speed compared to usual care or minimal activity.

These findings underscore that SUCRA values reflect only the relative probability of an intervention being ranked as the best, rather than the actual magnitude or statistical significance of its effect. Therefore, conclusions drawn solely from SUCRA rankings may be misleading. Clinical recommendations should be based on a comprehensive interpretation that integrates SUCRA rankings with effect sizes, confidence intervals, and statistical significance.

#### 3.4.3 Berg balance scale

A total of 12 high-quality randomized controlled trials were included in the analysis of the BBS outcome (Karaca and Kılınç, 2024; Lam et al., 2018; Pollock et al., 2012; Yang et al., 2023; Mesquita et al., 2015; Acheche et al., 2020; Yuzlu et al., 2022; Steadman et al., 2003; Hernández-Guillén et al., 2020; Mikó et al., 2018; Madureira et al., 2007; Madureira et al., 2010), involving 676 older adult participants. Sensitivity analysis (Supplementary Figure S12) using a leave-one-out approach revealed no individual study exerted an undue influence on the pooled effect estimate or SUCRA ranking. This indicates that the overall results for the BBS outcome were robust and not disproportionately driven by any single trial. As illustrated in Figure 3c, the network plot revealed the structure of direct comparisons among interventions. The most frequent comparisons were between BT and WBVT, whereas ST had relatively fewer direct comparisons with other training modalities. Additionally, a closed-loop structure was observed involving BT–WBVT–Control.

According to the loop inconsistency analysis presented in Supplementary Table S2, the 95% confidence intervals for the inconsistency factors (IFs) all included zero, indicating good consistency across the network with no statistically significant discrepancies between direct and indirect comparisons.

The network meta-analysis results are summarized in the Netleague table (Table 2) and visualized in the predictive interval plot (Supplementary Figure S4). None of the interventions showed statistically significant improvements in BBS scores compared to the control group. The standardized mean differences (SMD) and 95% confidence intervals were as follows: ST (SMD = −0.05; 95% CI: −1.29 to 1.19), WBVT (SMD = 0.57; 95% CI: −0.05 to 1.18), NT (SMD = −0.06; 95% CI: −0.88 to 0.75), and BT (SMD = 0.35; 95% CI: −0.10 to 0.80). All confidence intervals crossed the null value, and no statistically significant differences were detected in any of the direct pairwise comparisons between interventions.

The SUCRA-based cumulative probability rankings (Figure 4c) indicated the likelihood of each intervention being the most effective as follows: Control (70.3%), ST (63.3%), BT (31.6%), NT (69.3%), and WBVT (15.6%). While ST and NT appeared to rank higher than the other interventions, these findings should be interpreted with caution, as none of the estimated effect sizes demonstrated statistical significance when compared with the control group.

Although certain interventions showed higher SUCRA rankings, their effects on BBS scores were small and not statistically significant. This underscores that SUCRA reflects relative ranking rather than actual efficacy, and should be interpreted alongside effect sizes and clinical relevance. Current evidence does not support any NMT modality as a preferred intervention for improving static balance.

### 3.5 Heterogeneity assessment

To quantify between-study variability, we calculated the Q statistic, I^2^, and τ^2^ for each outcome and used Galbraith (radial) plots to visualise outliers and dispersion patterns.

**FIGURE 5:**
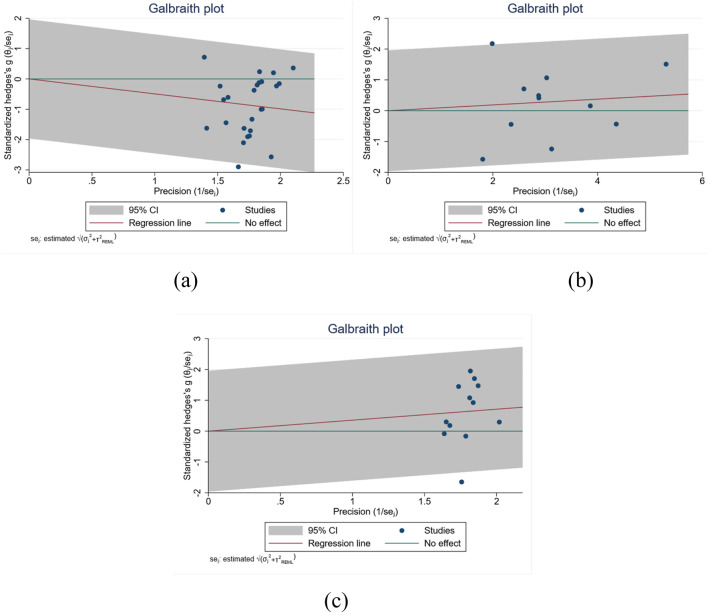
Galbraith plots for heterogeneity assessment in **(a)** TUGT, **(b)** WT, and **(c)** BBS.

Timed Up and Go Test (TUGT) (Figure 5a). Heterogeneity was pronounced (I^2^ = 76.98%, τ^2^ = 0.22, Q = 115.40, p < 0.001). Although most points clustered in the high-precision zone of the Galbraith plot, several high-weight studies lay outside the 95% confidence envelope, indicating influential outliers that largely account for the elevated I^2^.

Berg Balance Scale (BBS) (Figure 5b). Substantial heterogeneity was likewise observed (I^2^ = 73.83%, τ^2^ = 0.204, Q = 48.27, p < 0.001). The corresponding radial plot showed a mild “fan-shaped” spread in the high-precision region, suggesting that differences in sample characteristics, intervention protocols, or study design contributed to directional and magnitude discrepancies.

Walk Test (WT) (Figure 5c). By contrast, heterogeneity was modest (I^2^ = 36.46%, τ^2^ = 0.03, Q = 18.81, p = 0.04). Most studies fell within the confidence bounds of the Galbraith plot, indicating that between-study variance exerted only a limited influence on the pooled effect.

Given these patterns, we conducted prespecified subgroup analyses to identify potential sources of heterogeneity and to clarify the contexts and populations in which each neuromuscular training modality is most effective.

### 3.6 Subgroup analyses

To elucidate the potential sources of effect size variation, subgroup analyses were performed according to intervention modality, age range, and participants’ health conditions.

In the Timed Up and Go Test (TUGT) (Supplementary Figure S7), studies were stratified into four categories based on the intervention applied: ST, WBVT, NT, and BT. The ST and NT subgroups exhibited relatively stable outcomes, each producing a large effect size (Hedges’s g = −0.91) with low to moderate heterogeneity (I^2^ = 0% and 55.32%, respectively). In contrast, WBVT demonstrated a moderate impact (Hedges’s g = −0.30, I^2^ = 52.56%), while BT showed substantial inconsistency in treatment effects (Hedges’s g = −0.37, I^2^ = 84.27%). Notably, the between-group difference reached statistical significance (Q = 8.20, p = 0.04), highlighting the type of intervention as a key contributor to heterogeneity.

For the Walk Test (WT), subgrouping by age revealed that the 70–80-year cohort benefited most from the interventions (Hedges’s g = 0.27, I^2^ = 0%), while the <70 years (Hedges’s g = −0.01, I^2^ = 0%) and >80 years (Hedges’s g = 0.08, I^2^ = 58.51%) groups exhibited limited or inconsistent improvements (Supplementary Figure S10). When stratified by health status, a significant effect was observed only in participants with other conditions (Hedges’s g = 0.27, I^2^ = 0%). In contrast, both the stroke group (Hedges’s g = 0.04, I^2^ = 0%) and the healthy older adults (Hedges’s g = 0.03, I^2^ = 47.51%) did not show statistically meaningful outcomes. Although the test for subgroup differences did not reach statistical significance (Q = 2.67, p = 0.26) (Supplementary Figure S11), these stratified patterns offer valuable clinical insight into differential responsiveness.

With regard to the Berg Balance Scale (BBS), the 70–80-year subgroup again exhibited the most robust response to interventions (Hedges’s g = 0.64, I^2^ = 18.03%). In contrast, the <70 years group experienced no meaningful benefit (Hedges’s g = −0.37, I^2^ = 56.79%), while the >80 years subgroup showed a significant but less consistent effect (Hedges’s g = 0.54, I^2^ = 63.94%) (Supplementary Figure S13). Health-based subgrouping further revealed that participants with osteoporosis achieved the greatest gains (Hedges’s g = 0.72, I^2^ = 0%), followed by the healthy group with moderate effects (Hedges’s g = 0.44, I^2^ = 56.43%). In sharp contrast, the stroke subgroup showed not only a non-significant effect but also a reversed direction of change (Hedges’s g = −0.52, I^2^ = 67.68%). The between-subgroup difference was statistically significant (Q = 7.32, p = 0.03) (Supplementary Figure S14), suggesting that both age distribution and clinical condition may underlie much of the observed heterogeneity.

In summary, heterogeneity in TUGT outcomes appears predominantly driven by intervention modality, while variability in WT and BBS is more closely tied to participant age and health status. These findings underscore the importance of accounting for individual characteristics when designing and evaluating neuromuscular training protocols, and they reinforce the need for tailored intervention approaches in aging populations.

### 3.7 Publication bias and sensitivity analyses

To evaluate potential publication bias and the robustness of pooled estimates, we employed a combination of graphical and statistical methods, including funnel plot visualization, Egger’s regression intercept test, and leave-one-out sensitivity analyses.

**FIGURE 6:**
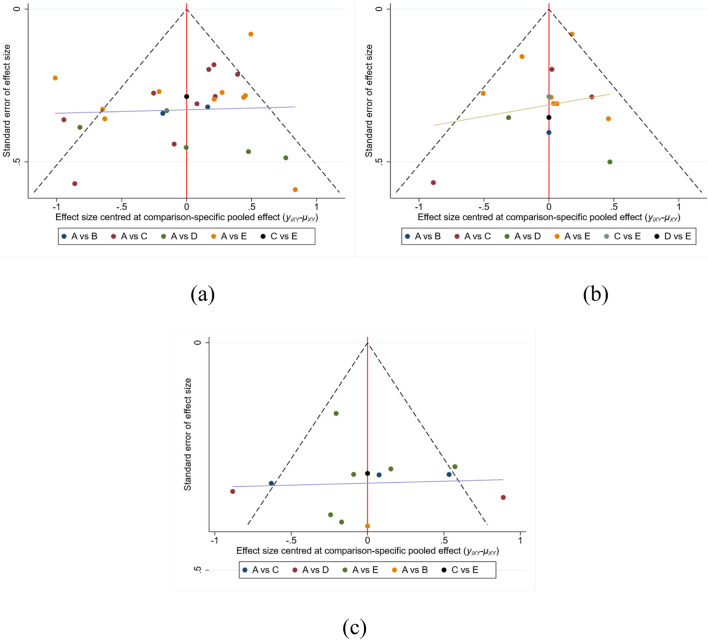
Funnel plots assessing publication bias for **(a)** TUGT, **(b)** WT, and **(c)** BBS.

Visual inspection of the comparison-adjusted funnel plots for the three outcome measures—TUGT, WT, and BBS (Figure 6a–c)—revealed largely symmetrical distributions, suggesting a low risk of publication bias. This was further substantiated by Egger’s test results (Supplementary Table S3), with non-significant intercepts for all outcomes (TUGT: p = 0.074; WT: p = 0.4761; BBS: p = 0.6908), indicating no evidence of small-study effects.

To assess the influence of individual studies on the overall effect estimates, leave-one-out sensitivity analyses were conducted for each outcome (Supplementary Figures S6, S9). The exclusion of any single study did not substantially alter the pooled effect sizes or their confidence intervals. No reversal of effect direction or significant widening of confidence bounds was observed, implying the overall estimates were robust to the omission of individual data points.

Collectively, these findings reinforce the credibility and methodological soundness of the synthesized evidence, with minimal susceptibility to publication bias or undue influence from any single trial.

## 4 Discussion

### 4.1 Main findings

This study presents a systematic synthesis of the comparative effectiveness of four NMT modalities, specifically ST, NT, WBVT, and BT, in enhancing balance performance among older adults. Based on evidence drawn from 49 randomized controlled trials, the findings not only reveal the relative strengths of each intervention but also clarify a structural asymmetry in the current body of research, particularly in the differential impacts on dynamic and static balance. These results provide an empirical foundation for future optimization of intervention protocols and offer theoretical insight for designing more targeted and individualized strategies to promote balance function in aging populations.

Among all interventions evaluated, ST (SMD = −0.92; 95% CI: −1.66, −0.18) and NT (SMD = −0.92; 95% CI: −1.44, −0.40) exhibited the most significant improvements in dynamic balance. Their effectiveness was especially pronounced in the TUGT, where both showed large effect sizes and ranked highest in SUCRA analyses. These findings suggest that ST and NT are particularly beneficial for enhancing gait initiation and postural adjustment, which are critical components of functional mobility. In contrast, although WBVT and BT achieved statistical significance in certain comparisons, their effect sizes were relatively small, and the potential for clinical translation appears more limited.

When comparing outcome measures, TUGT and the BBS were more effective in differentiating between intervention effects. ST and NT consistently demonstrated superior performance across both indicators, indicating a stable capacity to improve postural control and dynamic task performance. On the other hand, results from the WT were more dispersed. The number of studies available for certain comparison pathways was limited, and moderate heterogeneity was observed (I^2^ = 36.46%, p = 0.26), which warrants cautious interpretation in light of variability in intervention designs and sample characteristics.

Heterogeneity tests and subgroup analyses identified key sources of variability in intervention effects. Specifically, TUGT-related differences were primarily driven by intervention type, whereas variations in WT and BBS outcomes were more closely influenced by age and baseline health status. Stratifying by intervention modality and participant characteristics substantially reduced within-group variance and enhanced the interpretability and robustness of the findings.

It is important to note that SUCRA reflects relative ranking probabilities rather than absolute effect size or clinical value. For instance, although ST yielded the highest SUCRA for WT (73.7%), its effect size was not statistically significant (SMD = 0.13, 95% CI: −0.10 to 0.36), suggesting that a favorable ranking does not necessarily translate into meaningful clinical benefit. Thus, interpretation of SUCRA rankings should be contextualized by considering the magnitude of effect, width of the confidence interval, and whether the estimate meets thresholds for minimal clinically important difference (MCID), to avoid overgeneralization based solely on rank ordering.

Taken together, ST and NT demonstrated the most robust effects on dynamic balance, particularly in improving postural control during transitional and ambulatory tasks such as TUGT. WBVT may serve as a supplementary modality for enhancing specific neuromuscular functions, while BT appears more suited for maintaining static postural stability. These findings support a stratified, individualized approach to balance training, emphasizing the alignment of intervention modality with its underlying physiological mechanism, the target population’s characteristics, and the specific functional goals. This differentiation is especially critical in fall prevention, where distinct strategies are required for dynamic *versus* static balance improvement.

### 4.2 Comparison with previous studies

Compared to earlier studies, the present research expands both the scope of interventions and the methodological approach used to assess their comparative effectiveness. Prior investigations have typically focused on a single type of NMT or relied solely on conventional pairwise comparisons, which limited their ability to comprehensively evaluate the relative efficacy of multiple interventions within a unified framework (Zhang and Xiao, 2020; Abdelbasset et al., 2021; Aman et al., 2014; Sañudo et al., 2019). By utilizing a network meta-analysis (NMA) approach, this study represents the first comprehensive attempt to compare four major types of NMT interventions: ST, NT, WBVT, and BT. In addition to evaluating their individual effects, the study also establishes a relative ranking of efficacy, offering a more cohesive and evidence-driven foundation for selecting appropriate neuromuscular strategies in older adult populations.

The favorable performance of ST in enhancing dynamic balance aligns well with previous findings. ST improves an individual’s responsiveness to postural changes by reinforcing multisensory input and promoting neural integration. Numerous intervention studies in recent years have reported that ST produces greater improvements than conventional training programs (Zhang and Xiao, 2020; Abdelbasset et al., 2021; Aman et al., 2014). The present findings, particularly those derived from the TUGT, further consolidate this pattern of superiority.

In contrast, research on NT in older adults remains relatively scarce, with existing evidence largely drawn from rehabilitation or athletic populations. The findings across these populations have been inconsistent (Aman et al., 2014; Sañudo et al., 2019; Müller et al., 2023). By aggregating data from multiple RCTs, our study demonstrates that NT exerts a robust effect on TUGT performance, suggesting a potentially higher responsiveness to training that targets rapid neuromuscular adjustments and lower-limb coordination.

As for WBVT, although some studies have reported positive effects on lower-limb strength and bone mineral density, its influence on balance control has been less consistent and often inconclusive (Wang et al., 2022). The findings of the current study did not indicate significant improvements in balance-related outcomes associated with WBVT, suggesting that it may be more appropriately positioned as a complementary rather than a primary intervention.

Regarding BT, this modality represents a more traditional form of training and is primarily designed to support static postural control (Lesinski et al., 2015). In the present analysis, BT showed only modest improvements in dynamic tasks and consistently ranked lower across multiple outcome domains. These results point to its limited adaptability in addressing the more complex demands of dynamic balance training in aging populations.

Taken together, the findings of this study not only reinforce the theoretical underpinnings of certain established interventions but also provide clearer positioning for training modalities where prior evidence was insufficient or contradictory. This contributes to the development of a more precise and stratified framework for selecting and tailoring balance enhancement strategies in older adults.

### 4.3 Mechanism explanations

The observed effectiveness of NMT in improving balance performance among older adults appears to be underpinned by complex neurophysiological adaptations that occur across multiple levels of the sensorimotor system. Central to these adaptations are improvements in sensory integration, cortical activation, and peripheral muscular responsiveness, all of which contribute to enhanced postural control.

ST exerts its effects by facilitating the integration of multimodal sensory information, including visual, vestibular, and proprioceptive inputs. This process is thought to stimulate activity within the primary somatosensory cortex (S1) and premotor cortex (PMC), enhancing the brain’s capacity to detect postural disturbances and implement corrective motor strategies (Lephart et al., 1997; Zhang and Xiao, 2020; Shabir et al., 2021). As these sensorimotor pathways become more efficient, individuals demonstrate more automated and stable postural responses—particularly under dynamic conditions where anticipatory control is essential.

NT, by contrast, places older adults in cognitively or physically demanding task environments, which appear to activate deeper layers of neuromuscular function. This type of training has been associated with improved recruitment of spinal motor units and greater efficiency in corticospinal conduction. Of particular importance is its role in enhancing the activation of fast-twitch (Type II) muscle fibers, which are critical for rapid postural adjustments such as initiating gait or performing directional changes (Arumugam et al., 2021; Smaili et al., 2018). These adaptations likely account for the large effect sizes observed in TUGT performance, a finding that aligns with the high SUCRA rankings for both NT and ST.

In comparison, WBVT primarily elicits short-term neuromuscular responses via vibration-induced stretch reflexes. Although this can temporarily improve muscle strength and joint stability, the intervention may not sufficiently engage central integrative processes necessary for complex balance tasks. Consequently, its performance in gait-related or dual-task scenarios tends to be more limited (Bogaerts et al., 2007).

BT, a more traditional approach, relies predominantly on low-intensity static postural exercises. While such training may support foundational stability, it provides relatively weak stimuli to the central nervous system and exerts minimal influence on neuromuscular remodeling. This mechanistic limitation is consistent with BT’s lower effectiveness rankings observed in the SUCRA analysis and with its weaker impact on dynamic outcome measures (Sherrington et al., 2019).

Moreover, age and health status appear to modulate these neurophysiological pathways in distinct ways. Adults aged 70–80 years retain a meaningful reserve of neural plasticity and muscular strength, enabling more consistent adaptation to NMT and yielding sizeable, low-heterogeneity gains across several balance outcomes. By contrast, individuals with non-central-nervous-system conditions such as osteoporosis exhibit only mild functional compromise and intact neural circuits; their training responses are correspondingly uniform, resulting in large effect sizes with minimal heterogeneity. However, stroke survivors exhibit considerable inter-individual variability, which is influenced by factors such as lesion location, severity, rehabilitation history, and baseline functional status. This variability leads to substantial within-group dispersion and diminishes the magnitude of average treatment benefits. These observations underscore the need to consider both physiological reserves and pathological context when interpreting NMT responsiveness and tailoring mechanism-based interventions.

### 4.4 Theoretical and practical implications

Building upon the underlying neurophysiological mechanisms discussed above, this study offers a more nuanced understanding of how NMT improves balance in older adults. ST appears to enhance postural control primarily by facilitating the central integration of visual, proprioceptive, and vestibular inputs. This multisensory reinforcement improves the brain’s capacity to detect postural disturbances and to execute timely corrective actions. In contrast, NT emphasizes neuromuscular coordination by accelerating motor signal transmission and promoting the activation of fast-twitch muscle fibers responsible for rapid movement responses. These two training modalities function through distinct but complementary mechanisms. ST primarily enhances sensory integration, while NT strengthens reactive motor control. When combined, they provide compelling neurophysiological evidence supporting the concept of a sensorimotor synergy model in balance regulation.

From a practical perspective, the findings provide a rationale for tailoring intervention strategies to meet the specific functional deficits of older adults. ST may be especially beneficial for individuals experiencing sensory degradation, such as reduced proprioception or vestibular sensitivity. NT, on the other hand, is better suited for those with delayed reaction times or unstable gait patterns. When implemented in combination, these two approaches may form a more holistic intervention framework that addresses both sensory input processing and motor execution deficits.

It is noteworthy that, although both ST and NT consistently improve dynamic-balance metrics such as the TUGT, their effects on static balance (BBS) remain inconclusive. Accordingly, intervention plans should differentiate between dynamic and static deficits rather than apply a one-size-fits-all approach. For adults aged 70–80 years or those diagnosed with osteoporosis, who tend to exhibit homogeneous and substantial responses, ST and/or NT may be prioritized. In contrast, stroke survivors and very old adults may benefit more from extended intervention programs that integrate dynamic activities with targeted postural control exercises to achieve meaningful improvements across various balance domains.

It is recommended that training be conducted two to three times per week, with each session lasting between 40 and 60 min. The program content should be flexibly adjusted based on individual health status and mobility capacity. Such interventions hold strong potential for integration into community-based health promotion programs and may play a meaningful role in delaying functional decline, enhancing movement confidence, and reducing fall risk among older adults.

### 4.5 Limitations and future research

While this review offers valuable insights, several limitations must be considered when interpreting the findings. A major concern lies in the marked heterogeneity across intervention protocols. Studies differed substantially in training frequency, session duration, and overall program length, with little alignment in structural progression. This lack of standardization likely introduced variability in effect estimates and compromised cross-study comparability. Notably, training volume and delivery context (e.g., clinical vs. community-based settings) were seldom stratified or reported in sufficient detail, impeding dose–response analyses and limiting contextual interpretation.

Participant characteristics further constrain generalizability. Approximately 35% of participants presented with clinical conditions, potentially skewing results toward populations at elevated fall risk. In addition, nearly 68% of participants were women, creating a gender imbalance that may obscure effects among older men or individuals with multimorbidity. Variability in baseline functional capacity, age ranges, and health status also likely contributed to outcome heterogeneity and reduced the applicability of findings to more narrowly defined subgroups.

Another important limitation concerns the inconsistency of control group conditions. Control protocols ranged from no intervention to active comparators such as Tai Chi or resistance training. This variation may have confounded effect estimates and contributed to within-group variance, particularly in trials using active controls. Although such diversity mirrors real-world practice, it limits internal validity and complicates interpretation. Future studies should consider explicitly categorizing or stratifying control interventions to enable more rigorous synthesis and enhance comparability.

The strength of the evidence was further constrained by the limited number of trials informing certain outcomes, especially the WT and BBS. In these cases, network connectivity was weak and confidence intervals broad, diminishing both statistical precision and interpretive clarity. Moreover, methodological heterogeneity in balance assessment—stemming from the use of diverse instruments with varying scales and scoring systems—posed challenges for consistent synthesis and analytic coherence.

To advance the field, future research should emphasize methodological harmonization. Establishing consensus on optimal training frequency, session length, and content is essential, as is adopting validated, standardized outcome measures to minimize measurement error. Multicenter trials with extended follow-up are needed to assess sustained effects and to explore integrative models of neuromuscular adaptation. Economic evaluations should also be incorporated to determine cost-effectiveness, particularly for informing scalable interventions in aging societies.

Importantly, future studies should prioritize greater demographic diversity. Recruiting more male participants, the oldest-old, and individuals with complex health profiles will enhance external validity and better reflect the heterogeneity of the aging population. Through such improvements, future research can yield a more robust, inclusive evidence base for developing tailored, effective balance interventions that address the multifaceted needs of older adults.

## 5 Conclusion

This study offers a comparative synthesis of four primary neuromuscular training modalities, underscoring their differential effects on balance performance in older adults. Among them, ST and NT consistently yielded the most robust improvements in dynamic balance, likely attributable to their distinct neuromechanical activation pathways and their capacity to enhance postural adaptability. These findings underscore the value of a precision-based approach in selecting NMT strategies, emphasizing the need to align intervention modality with individual functional profiles and therapeutic goals in clinical and community settings.

## Data Availability

The original contributions presented in the study are included in the article/Supplementary Material, further inquiries can be directed to the corresponding author.
