# Supplementary material for: Effects of different neuromuscular training modalities on balance performance in older adults: a systematic review and network meta-analysis
Source: Front Physiol. 2025 Aug 8;16:1623908. doi: 10.3389/fphys.2025.1623908 (PMC12370742; doi:10.3389/fphys.2025.1623908)
Supplement: Supplementary file 1 [file DataSheet1.zip › Supplementary Materials/Figure S1 Risk of bias summary Methodological quality of each item for the each included study..pdf]

Intention-to-treat

Unique ID	Study ID	Experimental	Comparator	Outcome	Weight	D1	D2	D3	D4	D5	Overall
Karaca 2024	Karaca 2024	ST	Trunk-centered Bobath	BBS 2m-WT	1	+	!	+	+	+	!
Shabir 2021	Shabir 2021	ST	Conventional	FRT TUG OLST	1	+	+	+	+	+	+
Jimenez-Mazu	Jimenez-Mazuelas	ST	Conventional	FSST TUG FES	1	+	+	!	+	+	!
Espejo-Antun	Espejo-Antunez	ST	NA	TUG CPT TS OLST	1	+	+	+	+	+	+
Sinaki 2002	Sinaki 2002	ST	Exercise therapy	CDP	1	!	-	+	+	-	-
Stolzenberg	Stolzenberg 201	WBVT	BT	RST STST TST OLST	1	!	+	+	+	+	!
Tseng 2016a	Tseng 2016a	WBVT	No training program	LOST SRT	1	+	+	+	+	+	+
Tseng 2016b	Tseng 2016b	WBVT	No training program	LOST	1	+	+	+	+	+	+
Sievänen 202	Sievänen 2024	WBVT	Wellness	SPPB TUG 5t-CST GT	1	+	+	+	+	+	+
Bautmans 200	Bautmans 2005	WBVT	BT	TUG GT 30s-CST	1	+	+	+	+	+	+
Bogaerts 200	Bogaerts 2007	WBVT	Fitness	SOT	1	!	+	+	+	!	!
Nawrat-Szołty	Nawrat-Szołtysik	WBVT	No training program	TUG 6m-WT 30s-CST F	1	+	+	+	+	+	+
Lam 2018	Lam 2018	IG1: WBVT IG2: BT	Conventional	TUG TUG FTSST 6m-WT	1	+	+	+	+	+	+
Goudarzian 2	Goudarzian 2017	WBV + placebo	No training program	FBT TUG GT 30m-WT	1	+	+	+	+	+	+
Bogaerts 201	Bogaerts 2011	WBVT	No training program	SOT 10m-WT TUG	1	+	+	+	+	-	-
Zhang 2014	Zhang 2014	WBVT	Conventional exercise	TUG 30s-CST ABC	1	+	+	+	+	-	-
Pollock 2012	Pollock 2012	WBVT + Exercise	Combined exercise	TUG 6m-WT BBS FES-I	1	+	+	!	+	+	!
Asahina 2023	Asahina 2023	WBVT	No training program	TUG OLST OLST	1	+	+	+	+	+	+
Ko 2017	Ko 2017	WBVT	No training program	LOST STS	1	+	+	+	+	!	!
Yang 2023	Yang 2023	WBVT	WBVT	BBS CRT	1	+	-	+	+	!	-
Kang 2024	Kang 2024	NMT	Traditional training	TUG YBT RST	1	+	+	+	+	!	!
Zarzeczny 20	Zarzeczny 2024	NMT	Traditional training	TUG 30s-CST 6m-WT	1	+	+	+	+	+	+
Jang 2021	Jang 2021	NMT	No training program	FTSST TUG TUG YBT	1	+	+	+	+	+	+
Mesquita LSA	Mesquita LSA 201	NMT	No training program	TUG FRT BBS	1	+	+	+	+	+	+
Concha-Ciste	Concha-Cisternas	IG1: NMT IG2: BT	No training program	SPPB 6m-WT GT FTSS	1	+	+	+	+	+	+
Smaili 2018	Smaili 2018	NMT	RT	5m-WT GT	1	+	!	!	+	+	!
Acheche 2020	Acheche 2020	NMT	RT	TUG 6m-WT BBS	1	+	!	+	+	+	!
Yuzlu 2022	Yuzlu 2022	BT	Conventional exercise	BBS TUG 10m-WT FES	1	+	+	+	+	+	+
Rossi 2014	Rossi 2014	BT	No training program	TUG	1	+	+	+	+	+	+
Halvarsson 2	Halvarsson 2015	BT	No training program	FES GT OLST	1	+	+	+	+	+	+
An 2024	An 2024	BT	Conventional exercise	TUG 10m-WT	1	+	+	+	+	+	+
Steadman 200	Steadman 2003	BT	Conventional exercise	BBS 10m-WT	1	+	+	+	+	+	+
Hernández-Gu	Hernández-Guill	BT	Exercises	BBS	1	+	+	+	+	+	+
Lee 2012	Lee 2012	BT	No training program	TUG FRT OLST	1	!	-	+	+	+	-
Hirase 2015	Hirase 2015	BT	No training program	OLST CST TUG TST FE	1	+	+	+	+	!	!
El-Khoury 20	El-Khoury 2015	BT	No training program	TUG 6m-WT FTSST OLS	1	+	+	+	+	+	+
Miko 2018	Miko 2018	BT	Regular walking	TUG BBS RBT	1	+	+	+	+	+	+
Madureira 20	Madureira 2007	BT	No training program	BBS TUG	1	+	!	+	+	!	!
Madureira 20	Madureira 2010	BT	No Training Program	BBS	1	+	!	+	+	!	!
Markovic 201	Markovic 2015	BT	PT	SB-COP	1	+	+	+	+	+	+
Wallén 2018	Wallén 2018	BT	Conventional care	GT Mini-BESTest	1	+	+	+	+	+	+
Bao 2018	Bao 2018	BT	No training program	ABC SOT Mini-BESTes	1	+	+	+	+	+	+
BS 2012	BS 2012	BT	BT	8UG OLST FRT COP	1	+	+	+	+	+	+
SaNtos 2017	SaNtos 2017	BT	RT	RBT OLST BESTest	1	+	+	+	+	+	+
Carter ND 20	Carter ND 2001	BT	No training program	SB DB TFE Run	1	+	+	+	+	+	+
Sadeghi H 20	Sadeghi H 2021	BT	Daily activities	OLST TST TUG 10MWT	1	+	+	+	+	!	!
Sörlén 2021	Sörlén 2021	BT	No training program	TUG FES FES-I	1	+	+	+	+	+	+
Donath 2016	Donath 2016	BT	Regular daily activit	OLST YBT	1	+	!	+	+	!	!
Allin 2016	Allin 2016	BT	No training program	GT	1	+	+	+	+	+	+

+ Low risk
! Some concerns
- High risk

D1 Randomisation process
D2 Deviations from the intended interventions
D3 Missing outcome data
D4 Measurement of the outcome
D5 Selection of the reported result
