# Supplementary material for: Effects of different neuromuscular training modalities on balance performance in older adults: a systematic review and network meta-analysis
Source: Front Physiol. 2025 Aug 8;16:1623908. doi: 10.3389/fphys.2025.1623908 (PMC12370742; doi:10.3389/fphys.2025.1623908)
Supplement: Supplementary file 1 [file DataSheet1.zip › Supplementary Materials/Figure S3 Predictive interval plot of WT.pdf]

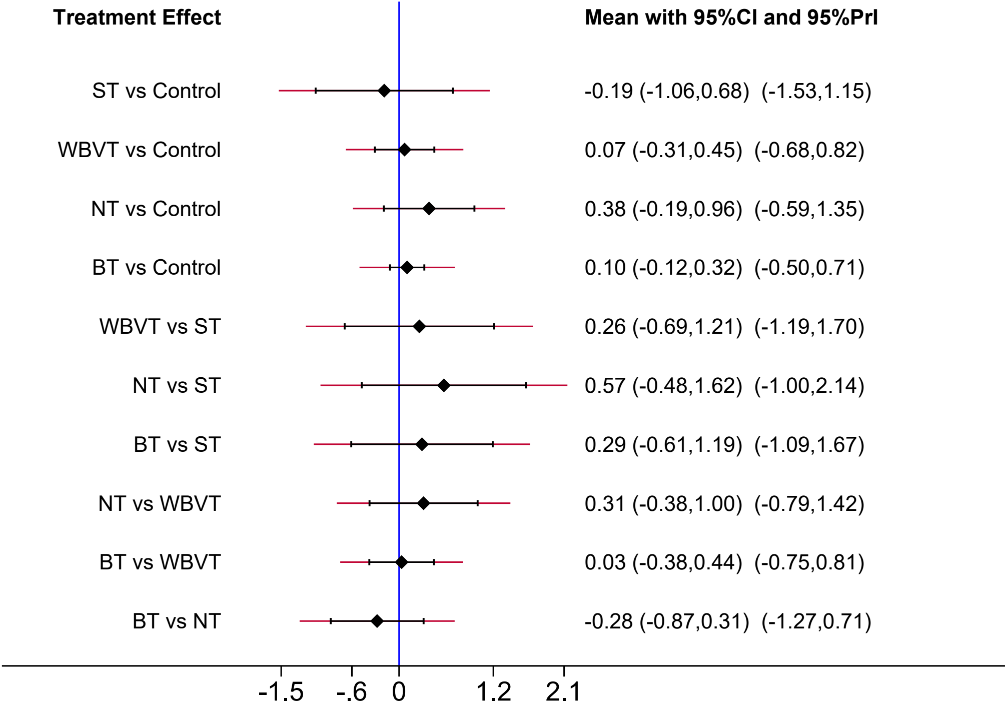
Treatment Effect
Mean with 95%CI and 95%PrI
ST vs Control
-0.19 (-1.06,0.68) (-1.53,1.15)
WBVT vs Control
0.07 (-0.31,0.45) (-0.68,0.82)
NT vs Control
0.38 (-0.19,0.96) (-0.59,1.35)
BT vs Control
0.10 (-0.12,0.32) (-0.50,0.71)
WBVT vs ST
0.26 (-0.69,1.21) (-1.19,1.70)
NT vs ST
0.57 (-0.48,1.62) (-1.00,2.14)
BT vs ST
0.29 (-0.61,1.19) (-1.09,1.67)
NT vs WBVT
0.31 (-0.38,1.00) (-0.79,1.42)
BT vs WBVT
0.03 (-0.38,0.44) (-0.75,0.81)
BT vs NT
-0.28 (-0.87,0.31) (-1.27,0.71)
-1.5
-.6
0
1.2
2.1
