# Supplementary material for: Effects of different neuromuscular training modalities on balance performance in older adults: a systematic review and network meta-analysis
Source: Front Physiol. 2025 Aug 8;16:1623908. doi: 10.3389/fphys.2025.1623908 (PMC12370742; doi:10.3389/fphys.2025.1623908)
Supplement: Supplementary file 1 [file DataSheet1.zip › Supplementary Materials/File S1 STATA Analysis Code and Dataset for NMA.docx]

**Network Meta-Analysis STATA CODE**

**TUGT Network Meta-Analysis DATA**

| **Study** | **Type** | **Mean** | **SD** | **N** |
| --- | --- | --- | --- | --- |
| **Shabir 2021** | **2** | **9** | **1.52** | **20** |
| **Shabir 2021** | **1** | **10.75** | **1.61** | **20** |
| **Espejo-Antunez 2020** | **2** | **15.74** | **6.21** | **21** |
| **Espejo-Antunez 2020** | **1** | **22.5** | **10.86** | **21** |
| **Sievänen 2024** | **3** | **12.6** | **25.59** | **63** |
| **Sievänen 2024** | **1** | **14.7** | **28.04** | **58** |
| **Bautmans 2005** | **3** | **12** | **3.7** | **10** |
| **Bautmans 2005** | **1** | **14.3** | **7.1** | **11** |
| **Nawrat-Szołtysik 2022** | **3** | **10.08** | **2.76** | **22** |
| **Nawrat-Szołtysik 2022** | **1** | **10.93** | **5.02** | **20** |
| **Lam 2017** | **3** | **41.5** | **35.9** | **25** |
| **Lam 2017** | **5** | **47.7** | **32.6** | **24** |
| **Lam 2017** | **1** | **43.8** | **31.5** | **24** |
| **Goudarzian2017** | **3** | **5.11** | **0.6** | **8** |
| **Goudarzian2017** | **1** | **6.12** | **1.03** | **7** |
| **Zhang 2014** | **3** | **21.34** | **4.42** | **19** |
| **Zhang 2014** | **1** | **30.39** | **9.24** | **18** |
| **Bogaerts 2010** | **3** | **10.95** | **3.46** | **50** |
| **Bogaerts 2010** | **1** | **11.41** | **4.22** | **53** |
| **Pollock 2012** | **3** | **18.83** | **9.36** | **24** |
| **Pollock 2012** | **1** | **24.47** | **10.86** | **32** |
| **Asahina 2023** | **3** | **10.9** | **5.4** | **42** |
| **Asahina 2023** | **1** | **10.4** | **4.1** | **46** |
| **Kang 2024** | **4** | **9.88** | **2.03** | **11** |
| **Kang 2024** | **1** | **11.98** | **2.34** | **11** |
| **Zarzeczny 2024** | **4** | **18.47** | **8.188** | **10** |
| **Zarzeczny 2024** | **1** | **22.9** | **10.887** | **9** |
| **Jang 2021** | **4** | **9.88** | **1.5** | **8** |
| **Jang 2021** | **1** | **10.1** | **1.18** | **9** |
| **Acheche 2020** | **4** | **10.06** | **0.48** | **22** |
| **Acheche 2020** | **1** | **10.71** | **0.7** | **20** |
| **Mesquita 2015** | **4** | **8.1** | **1.9** | **20** |
| **Mesquita 2015** | **1** | **13.9** | **4.3** | **18** |
| **Rossi 2014** | **5** | **10.12** | **1.36** | **23** |
| **Rossi 2014** | **1** | **10.3** | **1.8** | **23** |
| **Yuzlu 2021** | **5** | **10.72** | **2.25** | **25** |
| **Yuzlu 2021** | **1** | **10.88** | **3.768** | **29** |
| **An 2024** | **5** | **10.67** | **1.63** | **21** |
| **An 2024** | **1** | **12.67** | **2.35** | **21** |
| **Lee 2012** | **5** | **7.62** | **0.85** | **17** |
| **Lee 2012** | **1** | **9.41** | **2.42** | **18** |
| **Hirase 2015** | **5** | **12.1** | **4.2** | **29** |
| **Hirase 2015** | **1** | **14.6** | **5** | **28** |
| **El-Khoury 2015** | **5** | **12.85** | **4.27** | **306** |
| **El-Khoury 2015** | **1** | **12.1** | **4.45** | **294** |
| **Miko 2018** | **5** | **6.74** | **0.9** | **49** |
| **Miko 2018** | **1** | **10.64** | **4.02** | **48** |
| **Bao 2018** | **5** | **10.5** | **1.4** | **6** |
| **Bao 2018** | **1** | **9.8** | **1.1** | **6** |
| **Sörlén 2021** | **5** | **10** | **4.33** | **22** |
| **Sörlén 2021** | **1** | **9.5** | **3.36** | **29** |

**WT Network Meta-Analysis DATA**

| **Study** | **Type** | **Mean** | **SD** | **N** |
| --- | --- | --- | --- | --- |
| **Karaca 2024** | **2** | **38.87** | **36.15** | **11** |
| **Karaca 2024** | **1** | **45.3** | **30.22** | **14** |
| **Lam 2017** | **3** | **150.7** | **100.5** | **25** |
| **Lam 2017** | **5** | **128.6** | **101.9** | **24** |
| **Lam 2017** | **1** | **116.1** | **92.2** | **24** |
| **Goudarzian 2017** | **3** | **16.85** | **1.96** | **7** |
| **Goudarzian 2017** | **1** | **20.01** | **4.38** | **7** |
| **Bogaerts 2010** | **3** | **8.12** | **2.54** | **50** |
| **Bogaerts 2010** | **1** | **8.03** | **1.82** | **53** |
| **Zarzeczny 2024** | **4** | **205.25** | **92.339** | **10** |
| **Zarzeczny 2024** | **1** | **118.222** | **51.603** | **9** |
| **Concha-Cisternas 2024** | **4** | **3.77** | **0.596** | **16** |
| **Concha-Cisternas 2024** | **5** | **3.93** | **0.388** | **16** |
| **Concha-Cisternas 2024** | **1** | **3.57** | **0.817** | **16** |
| **Acheche 2020** | **5** | **542** | **136** | **22** |
| **Acheche 2020** | **1** | **523** | **68** | **20** |
| **Yuzlu 2021** | **5** | **1.14** | **0.249** | **25** |
| **Yuzlu 2021** | **1** | **1.25** | **0.291** | **29** |
| **An 2024** | **5** | **1** | **0.89** | **21** |
| **An 2024** | **1** | **0.88** | **0.74** | **21** |
| **Steadman 2003** | **5** | **16.3** | **6.3** | **82** |
| **Steadman 2003** | **1** | **17.3** | **12.6** | **84** |
| **El-Khoury 2015** | **5** | **14.85** | **1.68** | **306** |
| **El-Khoury 2015** | **1** | **14.37** | **1.69** | **294** |

**BBS Network Meta-Analysis DATA**

| **Study** | **Type** | **Mean** | **SD** | **N** |
| --- | --- | --- | --- | --- |
| **Karaca 2024** | **2** | **39.5** | **17.78** | **11** |
| **Karaca 2024** | **1** | **40.18** | **7.19** | **14** |
| **Lam 2017** | **3** | **39.5** | **12.5** | **25** |
| **Lam 2017** | **5** | **34.6** | **12.9** | **24** |
| **Lam 2017** | **1** | **31** | **15.5** | **24** |
| **Yang 2023** | **3** | **55.3** | **3.19** | **22** |
| **Yang 2023** | **1** | **55.65** | **4.34** | **20** |
| **Pollock 2012** | **3** | **48.5** | **5.49** | **24** |
| **Pollock 2012** | **1** | **42.67** | **5.26** | **32** |
| **Mesquita 2015** | **4** | **55.8** | **6.58** | **20** |
| **Mesquita 2015** | **1** | **51** | **4.34** | **18** |
| **Acheche 2020** | **4** | **53.68** | **1.78** | **22** |
| **Acheche 2020** | **1** | **55.1** | **1.07** | **20** |
| Yuzlu 2021 | **5** | **47.23** | **2.89** | **25** |
| Yuzlu 2021 | **1** | **45.69** | **3.11** | **29** |
| **Steadman 2003** | **5** | **41.2** | **9.3** | **82** |
| **Steadman 2003** | **1** | **39.8** | **9.8** | **84** |
| **Hernández-Guillén 2019** | **5** | **52.2** | **5.94** | **14** |
| **Hernández-Guillén 2019** | **1** | **51.5** | **6.45** | **14** |
| **Miko 2018** | **5** | **49.23** | **1.33** | **12** |
| **Miko 2018** | **1** | **48.52** | **5** | **14** |
| **Madureira** 2007 | **5** | **52.84** | **6.73** | **34** |
| **Madureira**  2007 | **1** | **47.6** | **6.42** | **32** |
| **Madureira 2010** | **5** | **54.3** | **7** | **30** |
| **Madureira 2010** | **1** | **47.63** | **7.25** | **30** |

**Installing network meta-analysis packages in Stata**

ssc install

search network

search mvmeta

ssc install metareg

**1. Data Standardization and Preprocessing**

**Code:** network setup mean sd n,studyvar(study) trtvar(type) ref(1) smd

**2. The data format was further transformed into study-level data.**

**Code:** network convert pairs

**3. Construction of the network evidence plot**

**Code:** gen total=n1+n2

networkplot _t1 _t2, lab(Control ST WBVT NT BT) nodew(total sum)

**4. Construction of the contribution plot**

**Code:** netweight _y _stderr _t1 _t2, color(navy) symbol(circle)

**5. Construction of funnel plots**

**Code:** netfunnel _y _stderr _t1 _t2, random bycomp add(lfit _stderr _ES_CEN)

**6. IF plot generation (loop inconsistency assessment)**

**Code:** ifplot _y _stderr _t1 _t2 study, tau2(loop) lab(Control ST WBVT NT BT)

**7. Fitting inconsistency models (global inconsistency assessment)**

**Code:** network convert augment

network meta i

**8. Node-splitting method**

**Code:** network sidesplit all

**9. Forest plot**

**Code:** network forest

**10. Fitting a consistency model**

**Code:** network meta c

**11. Net league table**

**Code:** netleague, lab(Control ST WBVT NT BT) sort(ST WBVT NT BT Control) nokeep export("C:\Users\XXXX\Desktop\XXXX.xlsx")

**12. Prediction interval plot**

**Code:** intervalplot, pred null(0) lab(Control ST WBVT NT BT)

**13. . SUCRA ranking probability plot**

**Code:** network rank min, zero all reps(10000) gen(prob)

sucra prob*, labels(Control ST WBVT NT BT) lcol(red)

**Meta-Analysis STATA CODE**

**TUGT Subgroup Analyses DATA**

| **Study** | **Invention** | **Expmean** | **Expsd** | **Expn** | **Cotrmean** | **Cotrsd** | **Cotrn** |
| --- | --- | --- | --- | --- | --- | --- | --- |
| **Shabir 2021** | 1 | **9** | **1.52** | **20** | **10.75** | **1.61** | **20** |
| **Espejo-Antunez 2020** | 1 | **15.74** | **6.21** | **21** | **22.5** | **10.86** | **21** |
| **Sievänen 2024** | 2 | **12.6** | **25.59** | **63** | **14.7** | **28.04** | **58** |
| **Bautmans 2005** | 2 | **12** | **3.7** | **10** | **14.3** | **7.1** | **11** |
| **Nawrat-Szołtysik 2022** | 2 | **10.08** | **2.76** | **22** | **10.93** | **5.02** | **20** |
| **Lam 2017** | 2 | **41.5** | **35.9** | **25** | **43.8** | **31.5** | **24** |
| **Goudarzian2017** | 2 | **5.11** | **0.6** | **8** | **6.12** | **1.03** | **7** |
| **Zhang 2014** | 2 | **21.34** | **4.42** | **19** | **30.39** | **9.24** | **18** |
| **Bogaerts 2010** | 2 | **10.95** | **3.46** | **50** | **11.41** | **4.22** | **53** |
| **Pollock 2012** | 2 | **18.83** | **9.36** | **24** | **24.47** | **10.86** | **32** |
| **Asahina 2023** | 2 | **10.9** | **5.4** | **42** | **10.4** | **4.1** | **46** |
| **Kang 2024** | 3 | **9.88** | **2.03** | **11** | **11.98** | **2.34** | **11** |
| **Zarzeczny 2024** | 3 | **18.47** | **8.188** | **10** | **22.9** | **10.887** | **9** |
| **Jang 2021** | 3 | **9.88** | **1.5** | **8** | **10.1** | **1.18** | **9** |
| **Acheche 2020** | 3 | **10.06** | **0.48** | **22** | **10.71** | **0.7** | **20** |
| **Mesquita 2015** | 3 | **8.1** | **1.9** | **20** | **13.9** | **4.3** | **18** |
| **Rossi 2014** | 4 | **10.12** | **1.36** | **23** | **10.3** | **1.8** | **23** |
| **Yuzlu 2021** | 4 | **10.72** | **2.25** | **25** | **10.88** | **3.768** | **29** |
| **An 2024** | 4 | **10.67** | **1.63** | **21** | **12.67** | **2.35** | **21** |
| **Lee 2012** | 4 | **7.62** | **0.85** | **17** | **9.41** | **2.42** | **18** |
| **Hirase 2015** | 4 | **12.1** | **4.2** | **29** | **14.6** | **5** | **28** |
| **El-Khoury 2015** | 4 | **12.85** | **4.27** | **306** | **12.1** | **4.45** | **294** |
| **Miko 2018** | 4 | **6.74** | **0.9** | **49** | **10.64** | **4.02** | **48** |
| **Bao 2018** | 4 | **10.5** | **1.4** | **6** | **9.8** | **1.1** | **6** |
| **Sörlén 2021** | 4 | **10** | **4.33** | **22** | **9.5** | **3.36** | **29** |

**WT Subgroup Analyses DATA (Age)**

| **Study** | **Age** | **Expmean** | **Expsd** | **Expn** | **Cotrmean** | **Cotrsd** | **Cotrn** |
| --- | --- | --- | --- | --- | --- | --- | --- |
| **Karaca 2024** | **1** | **38.87** | **36.15** | **11** | **45.3** | **30.22** | **14** |
| **Lam 2017** | **3** | **150.7** | **100.5** | **25** | **116.1** | **92.2** | **24** |
| **Goudarzian 2017** | **1** | **16.85** | **1.96** | **7** | **20.01** | **4.38** | **7** |
| **Bogaerts 2010** | **3** | **8.12** | **2.54** | **50** | **8.03** | **1.82** | **53** |
| **Zarzeczny 2024** | **3** | **205.25** | **92.339** | **10** | **118.222** | **51.603** | **9** |
| **Concha-Cisternas 2024** | **1** | **3.77** | **0.596** | **16** | **3.57** | **0.817** | **16** |
| **Acheche 2020** | **1** | **542** | **136** | **22** | **523** | **68** | **20** |
| **Yuzlu 2021** | **3** | **1.14** | **0.249** | **25** | **1.25** | **0.291** | **29** |
| **An 2024** | **2** | **1** | **0.89** | **21** | **0.88** | **0.74** | **21** |
| **Steadman 2003** | **3** | **16.3** | **6.3** | **82** | **17.3** | **12.6** | **84** |
| **El-Khoury 2015** | **2** | **14.85** | **1.68** | **306** | **14.37** | **1.69** | **294** |

**WT Subgroup Analyses DATA (Health)**

| **Study** | **Health** | **Expmean** | **Expsd** | **Expn** | **Cotrmean** | **Cotrsd** | **Cotrn** |
| --- | --- | --- | --- | --- | --- | --- | --- |
| **Karaca 2024** | **2** | **38.87** | **36.15** | **11** | **45.3** | **30.22** | **14** |
| **Lam 2017** | **1** | **150.7** | **100.5** | **25** | **116.1** | **92.2** | **24** |
| **Goudarzian 2017** | **1** | **16.85** | **1.96** | **7** | **20.01** | **4.38** | **7** |
| **Bogaerts 2010** | **1** | **8.12** | **2.54** | **50** | **8.03** | **1.82** | **53** |
| **Zarzeczny 2024** | **1** | **205.25** | **92.339** | **10** | **118.222** | **51.603** | **9** |
| **Concha-Cisternas 2024** | **1** | **3.77** | **0.596** | **16** | **3.57** | **0.817** | **16** |
| **Acheche 2020** | **2** | **542** | **136** | **22** | **523** | **68** | **20** |
| **Yuzlu 2021** | **1** | **1.14** | **0.249** | **25** | **1.25** | **0.291** | **29** |
| **An 2024** | **3** | **1** | **0.89** | **21** | **0.88** | **0.74** | **21** |
| **Steadman 2003** | **1** | **16.3** | **6.3** | **82** | **17.3** | **12.6** | **84** |
| **El-Khoury 2015** | **3** | **14.85** | **1.68** | **306** | **14.37** | **1.69** | **294** |

**BBS Subgroup Analyses DATA (Age)**

| **Study** | **Age** | **Expmean** | **Expsd** | **Expn** | **Cotrmean** | **Cotrsd** | **Cotrn** |
| --- | --- | --- | --- | --- | --- | --- | --- |
| **Karaca 2024** | **1** | **39.5** | **17.78** | **11** | **40.18** | **7.19** | **14** |
| **Lam 2017** | **3** | **39.5** | **12.5** | **25** | **31** | **15.5** | **24** |
| **Yang 2023** | **1** | **55.3** | **3.19** | **22** | **55.65** | **4.34** | **20** |
| **Pollock 2012** | **3** | **48.5** | **5.49** | **24** | **42.67** | **5.26** | **32** |
| **Mesquita 2015** | **2** | **55.8** | **6.58** | **20** | **51** | **4.34** | **18** |
| **Acheche 2020** | **1** | **53.68** | **1.78** | **22** | **55.1** | **1.07** | **20** |
| Yuzlu 2021 | **3** | **47.23** | **2.89** | **25** | **45.69** | **3.11** | **29** |
| **Steadman 2003** | **3** | **41.2** | **9.3** | **82** | **39.8** | **9.8** | **84** |
| **Hernández-Guillén 2019** | **2** | **52.2** | **5.94** | **14** | **51.5** | **6.45** | **14** |
| **Miko 2018** | **2** | **49.23** | **1.33** | **12** | **48.52** | **5** | **14** |
| **Madureira** 2007 | **2** | **52.84** | **6.73** | **34** | **47.6** | **6.42** | **32** |
| **Madureira 2010** | **2** | **54.3** | **7** | **30** | **47.63** | **7.25** | **30** |

**BBS Subgroup Analyses DATA (Health)**

| **Study** | **Health** | **Expmean** | **Expsd** | **Expn** | **Cotrmean** | **Cotrsd** | **Cotrn** |
| --- | --- | --- | --- | --- | --- | --- | --- |
| **Karaca 2024** | **2** | **39.5** | **17.78** | **11** | **40.18** | **7.19** | **14** |
| **Lam 2017** | **1** | **39.5** | **12.5** | **25** | **31** | **15.5** | **24** |
| **Yang 2023** | **1** | **55.3** | **3.19** | **22** | **55.65** | **4.34** | **20** |
| **Pollock 2012** | **1** | **48.5** | **5.49** | **24** | **42.67** | **5.26** | **32** |
| **Mesquita 2015** | **1** | **55.8** | **6.58** | **20** | **51** | **4.34** | **18** |
| **Acheche 2020** | **2** | **53.68** | **1.78** | **22** | **55.1** | **1.07** | **20** |
| Yuzlu 2021 | **1** | **47.23** | **2.89** | **25** | **45.69** | **3.11** | **29** |
| **Steadman 2003** | **1** | **41.2** | **9.3** | **82** | **39.8** | **9.8** | **84** |
| **Hernández-Guillén 2019** | **1** | **52.2** | **5.94** | **14** | **51.5** | **6.45** | **14** |
| **Miko 2018** | **3** | **49.23** | **1.33** | **12** | **48.52** | **5** | **14** |
| **Madureira** 2007 | **3** | **52.84** | **6.73** | **34** | **47.6** | **6.42** | **32** |
| **Madureira 2010** | **3** | **54.3** | **7** | **30** | **47.63** | **7.25** | **30** |

**Installing network meta-analysis packages in Stata**

ssc install metan

ssc install metareg

ssc install metafunnel

ssc install metaninf

ssc install metabias,replace

**1. Data Standardization and Preprocessing**

**Code:** metan expn expmean expsd cotrn cotrmean cotrsd, fixed label(namevar= study)

**2. Significance Test for Random-Effects Model**

**Code:** metareg _ES,wsse( _seES ) eform

**3. Sensitivity Analyses**

**Code:** metaninf _ES _seES,id( study )

**4. To ensure methodological rigor and transparency, heterogeneity assessments, subgroup analyses, and Egger’s test were manually executed in STATA, allowing for greater control over model specification and analytical accuracy.**
