# Supplementary material for: Effects of different neuromuscular training modalities on balance performance in older adults: a systematic review and network meta-analysis
Source: Front Physiol. 2025 Aug 8;16:1623908. doi: 10.3389/fphys.2025.1623908 (PMC12370742; doi:10.3389/fphys.2025.1623908)
Supplement: Supplementary file 1 [file DataSheet1.zip › Supplementary Materials/Supplementary Materials A RoB2 .pdf]

**Unique ID**	Karaca 2024	**Study ID**	Karaca 2024	**Assessor**	Chen
**Ref or Label**		**Aim**	assignment to intervention (the 'intention-to-treat' effect)		
**Experimental**	ST	**Comparator**	Trunk-centered Bobath exercises	**Source**	Journal article(s); Trial protocol; Non-commercial trial registry record (e.g. ClinicalTrials.gov record)
**Outcome**	BBS 2m-WT	**Results**		**Weight**	1

Domain	Signalling question	Response	Comments
**Bias arising from the randomization process**	1.1 Was the allocation sequence random?	Y	This was a single-blinded randomized controlled study using a parallel group design. Patients who par ticipated in the present study were randomized by a computer
	1.2 Was the allocation sequence concealed until participants were enrolled and assigned to interventions?	Y	
	1.3 Did baseline differences between intervention groups suggest a problem with the randomization process?	N	For the study, 68 patients were interviewed and 27 patients participated in randomization. [illegible]
	**Risk of bias judgement**	**Low**	
**Bias due to deviations from intended interventions**	2.1.Were participants aware of their assigned intervention during the trial?	PN	This was a single-blinded randomized controlled study using a parallel group design. Patients who par ticipated in the present study were randomized by a computer
	2.2.Were carers and people delivering the interventions aware of participants' assigned intervention during the trial?	N	
	2.3. If Y/PY/NI to 2.1 or 2.2: Were there deviations from the intended intervention that arose because of the experimental context?	NA	
	2.4 If Y/PY to 2.3: Were these deviations likely to have affected the outcome?	NA	
	2.5. If Y/PY/NI to 2.4: Were these deviations from intended intervention balanced between groups?	NA	
	2.6 Was an appropriate analysis used to estimate the effect of assignment to intervention?	PN	Statistical analyses of the data were conducted with [illegible]
	2.7 If N/PN/NI to 2.6: Was there potential for a substantial impact (on the result) of the failure to analyse participants in the group to which they were randomized?	PN	
	**Risk of bias judgement**	**Some concerns**	
**Bias due to missing outcome data**	3.1 Were data for this outcome available for all, or nearly all, participants randomized?	PY	This was a single-blinded randomized controlled [illegible]
	3.2 If N/PN/NI to 3.1: Is there evidence that result was not biased by missing outcome data?	NA	
	3.3 If N/PN to 3.2: Could missingness in the outcome depend on its true value?	NA	
	3.4 If Y/PY/NI to 3.3: Is it likely that missingness in the outcome depended on its true value?	NA	
	**Risk of bias judgement**	**Low**	
**Bias in measurement of the outcome**	4.1 Was the method of measuring the outcome inappropriate?	N	The assessments were performed twice, at baseline and [illegible]
	4.2 Could measurement or ascertainment of the outcome have differed between intervention groups?	N	The duration of exercise applied in addition to con ventional treatment was equal in both [illegible]
	4.3 Were outcome assessors aware of the intervention received by study participants?	N	This was a single-blinded randomized controlled study using a parallel group design. [illegible]
	4.4 If Y/PY/NI to 4.3: Could assessment of the outcome have been influenced by knowledge of intervention received?	NA	
	4.5 If Y/PY/NI to 4.4: Is it likely that assessment of the outcome was influenced by knowledge of intervention received?	NA	
	**Risk of bias judgement**	**Low**	
**Bias in selection of the reported result**	5.1 Were the data that produced this result analysed in accordance with a pre-specified analysis plan that was finalized before unblinded outcome data were available for analysis?	PY	The protocol for this trial is available (ClinicalTrials.gov Identifier: NCT04753931)
	5.2 ... multiple eligible outcome measurements (e.g. scales, definitions, time points) within the outcome domain?	N	Only one standard measurement tool is used in each area(TIS, TRE, BBS, 2MWT, BI).
	5.3 ... multiple eligible analyses of the data?	N	
	**Risk of bias judgement**	**Low**	
**Overall bias**	**Risk of bias judgement**	**Some concerns**	

**Unique ID**	Shabir 2021	**Study ID**	Shabir 2021	**Assessor**	Chen
**Ref or Label**		**Aim**	assignment to intervention (the 'intention-to-treat' effect)		
**Experimental**	ST	**Comparator**	Conventional	**Source**	Journal article(s); Trial protocol; Non-commercial trial registry record (e.g. ClinicalTrials.gov record)
**Outcome**	FRT TUG OLST	**Results**		**Weight**	1

Domain	Signalling question	Response	Comments
**Bias arising from the randomization process**	1.1 Was the allocation sequence random?	Y	Women were equally allocated (each group=22) in experimental (A) and controlled (B) groups via computerized method. Randomization sequence computer-generated
	1.2 Was the allocation sequence concealed until participants were enrolled and assigned to interventions?	Y	
	1.3 Did baseline differences between intervention groups suggest a problem with the randomization process?	N	Baseline values of age, BMI, and outcome variables (FRT, TUG, OLS) in Table 2 [illegible]
	**Risk of bias judgement**	**Low**	
**Bias due to deviations from intended interventions**	2.1.Were participants aware of their assigned intervention during the trial?	Y	The study was not blinded, and interventions were different in content and form (e.g., sensorimotor training vs. conventional therapy).
	2.2.Were carers and people delivering the interventions aware of participants' assigned intervention during the trial?	Y	
	2.3. If Y/PY/NI to 2.1 or 2.2: Were there deviations from the intended intervention that arose because of the experimental context?	N	
	2.4 If Y/PY to 2.3: Were these deviations likely to have affected the outcome?	NA	
	2.5. If Y/PY/NI to 2.4: Were these deviations from intended intervention balanced between groups?	NA	
	2.6 Was an appropriate analysis used to estimate the effect of assignment to intervention?	Y	By following CONSORT guidelines final analysis done with 40 patients, 20 in group A [illegible]
	2.7 If N/PN/NI to 2.6: Was there potential for a substantial impact (on the result) of the failure to analyse participants in the group to which they were randomized?	NA	
	**Risk of bias judgement**	**Low**	
**Bias due to missing outcome data**	3.1 Were data for this outcome available for all, or nearly all, participants randomized?	Y	Final analysis done with 40 patients, 20 in group A and 20 in group B." [illegible]
	3.2 If N/PN/NI to 3.1: Is there evidence that result was not biased by missing outcome data?	NA	
	3.3 If N/PN to 3.2: Could missingness in the outcome depend on its true value?	NA	
	3.4 If Y/PY/NI to 3.3: Is it likely that missingness in the outcome depended on its true value?	NA	
	**Risk of bias judgement**	**Low**	
**Bias in measurement of the outcome**	4.1 Was the method of measuring the outcome inappropriate?	N	Used validated measures: Functional Reach Test (FRT) [illegible]
	4.2 Could measurement or ascertainment of the outcome have differed between intervention groups?	N	Outcomes were standardized and applied equally at baseline and 6 weeks in both [illegible]
	4.3 Were outcome assessors aware of the intervention received by study participants?	PY	No mention of blinded assessors
	4.4 If Y/PY/NI to 4.3: Could assessment of the outcome have been influenced by knowledge of intervention received?	PN	
	4.5 If Y/PY/NI to 4.4: Is it likely that assessment of the outcome was influenced by knowledge of intervention received?	NA	
	**Risk of bias judgement**	**Low**	
**Bias in selection of the reported result**	5.1 Were the data that produced this result analysed in accordance with a pre-specified analysis plan that was finalized before unblinded outcome data were available for analysis?	PY	Clinical Trials.gov Identifier: NCT04820738 The study was preregistered, but the analysis [illegible]
	5.2 ... multiple eligible outcome measurements (e.g. scales, definitions, time points) within the outcome domain?	N	Each outcome domain had a single test (e.g., FRT for dynamic balance, TUG for mobility, [illegible]
	5.3 ... multiple eligible analyses of the data?	N	Only independent t-tests and pre-post comparisons were used and transparently [illegible]
	**Risk of bias judgement**	**Low**	
**Overall bias**	**Risk of bias judgement**	**Low**	

**Unique ID**	Jimenez-Mazuelas 2024	**Study ID**	Jimenez-Mazuelas 2024	**Assessor**	Chen
**Ref or Label**		**Aim**	assignment to intervention (the 'intention-to-treat' effect)		
**Experimental**	ST	**Comparator**	Conventional	**Source**	Journal article(s); Trial protocol; Non-commercial trial registry record (e.g. ClinicalTrials.gov record)
**Outcome**	FSST TUG FES	**Results**		**Weight**	1

Domain	Signalling question	Response	Comments
**Bias arising from the randomization process**	1.1 Was the allocation sequence random?	Y	Randomization was possible through computer-generated random number tables created by the Statistical Department. Participants randomly took an opaque [illegible]
	1.2 Was the allocation sequence concealed until participants were enrolled and assigned to interventions?	Y	
	1.3 Did baseline differences between intervention groups suggest a problem with the randomization process?	N	The baseline comparison between groups did not reveal any significant differences, as [illegible]
	**Risk of bias judgement**	**Low**	
**Bias due to deviations from intended interventions**	2.1.Were participants aware of their assigned intervention during the trial?	Y	The training instructor was not blinded. All the examinations and outcome measurements were performed by blinded collaborators.
	2.2.Were carers and people delivering the interventions aware of participants' assigned intervention during the trial?	Y	
	2.3. If Y/PY/NI to 2.1 or 2.2: Were there deviations from the intended intervention that arose because of the experimental context?	N	No adverse events were reported. The study was completed as scheduled." [illegible]
	2.4 If Y/PY to 2.3: Were these deviations likely to have affected the outcome?	NA	
	2.5. If Y/PY/NI to 2.4: Were these deviations from intended intervention balanced between groups?	NA	
	2.6 Was an appropriate analysis used to estimate the effect of assignment to intervention?	Y	The statistical analysis was performed on an intention-to-treat basis.
	2.7 If N/PN/NI to 2.6: Was there potential for a substantial impact (on the result) of the failure to analyse participants in the group to which they were randomized?	NA	
	**Risk of bias judgement**	**Low**	
**Bias due to missing outcome data**	3.1 Were data for this outcome available for all, or nearly all, participants randomized?	PN	The initial sample comprised 44 participants, and the final sample included 27 participants.
	3.2 If N/PN/NI to 3.1: Is there evidence that result was not biased by missing outcome data?	Y	
	3.3 If N/PN to 3.2: Could missingness in the outcome depend on its true value?	NA	
	3.4 If Y/PY/NI to 3.3: Is it likely that missingness in the outcome depended on its true value?	NA	
	**Risk of bias judgement**	**Some concerns**	Through the data offered in the reference study , and the statistic program Nquiry [illegible]
**Bias in measurement of the outcome**	4.1 Was the method of measuring the outcome inappropriate?	N	CSII、POMA、TUG、FES
	4.2 Could measurement or ascertainment of the outcome have differed between intervention groups?	N	All the examinations and outcome measurements were performed by blinded [illegible]
	4.3 Were outcome assessors aware of the intervention received by study participants?	N	All the examinations and outcome measurements were performed by blinded [illegible]
	4.4 If Y/PY/NI to 4.3: Could assessment of the outcome have been influenced by knowledge of intervention received?	NA	
	4.5 If Y/PY/NI to 4.4: Is it likely that assessment of the outcome was influenced by knowledge of intervention received?	NA	
	**Risk of bias judgement**	**Low**	
**Bias in selection of the reported result**	5.1 Were the data that produced this result analysed in accordance with a pre-specified analysis plan that was finalized before unblinded outcome data were available for analysis?	Y	eated three times until all the data were collected in December 2019. The study was [illegible]
	5.2 ... multiple eligible outcome measurements (e.g. scales, definitions, time points) within the outcome domain?	N	SF-36,POMA-B and POMA-G
	5.3 ... multiple eligible analyses of the data?	N	
	**Risk of bias judgement**	**Low**	
**Overall bias**	**Risk of bias judgement**	**Some concerns**	

**Unique ID**	Espejo-Antunez 2020	**Study ID**	Espejo-Antunez 2020	**Assessor**	Chen
**Ref or Label**		**Aim**	assignment to intervention (the 'intention-to-treat' effect)		
**Experimental**	ST	**Comparator**		**Source**	Journal article(s); Trial protocol; Non-commercial trial registry record (e.g. ClinicalTrials.gov record)
**Outcome**	TUG CPT TS OLST	**Results**	Conventional	**Weight**	1

Domain	Signalling question	Response	Comments
**Bias arising from the randomization process**	1.1 Was the allocation sequence random?	Y	Participants were randomly allocated to the experimental or control groups. The randomization was performed by asking the participant to pick a number out of an [illegible]
	1.2 Was the allocation sequence concealed until participants were enrolled and assigned to interventions?	PY	
	1.3 Did baseline differences between intervention groups suggest a problem with the randomization process?	N	There were no significant baseline differences between the treatment [illegible]
	**Risk of bias judgement**	**Low**	
**Bias due to deviations from intended interventions**	2.1.Were participants aware of their assigned intervention during the trial?	Y	It was not possible to conceal the group assignment from the researchers involved in the intervention.
	2.2.Were carers and people delivering the interventions aware of participants' assigned intervention during the trial?	Y	
	2.3. If Y/PY/NI to 2.1 or 2.2: Were there deviations from the intended intervention that arose because of the experimental context?	N	The interventions were supervised... adherence rate of 75% was established... [illegible]
	2.4 If Y/PY to 2.3: Were these deviations likely to have affected the outcome?	NA	
	2.5. If Y/PY/NI to 2.4: Were these deviations from intended intervention balanced between groups?	NA	
	2.6 Was an appropriate analysis used to estimate the effect of assignment to intervention?	Y	
	2.7 If N/PN/NI to 2.6: Was there potential for a substantial impact (on the result) of the failure to analyse participants in the group to which they were randomized?	NA	
	**Risk of bias judgement**	**Low**	
**Bias due to missing outcome data**	3.1 Were data for this outcome available for all, or nearly all, participants randomized?	Y	The final sample (N242) was randomly divided into control and experimental [illegible]
	3.2 If N/PN/NI to 3.1: Is there evidence that result was not biased by missing outcome data?	NA	
	3.3 If N/PN to 3.2: Could missingness in the outcome depend on its true value?	NA	
	3.4 If Y/PY/NI to 3.3: Is it likely that missingness in the outcome depended on its true value?	NA	
	**Risk of bias judgement**	**Low**	
**Bias in measurement of the outcome**	4.1 Was the method of measuring the outcome inappropriate?	N	TUG、OLS、Tinetti、MFS
	4.2 Could measurement or ascertainment of the outcome have differed between intervention groups?	N	The rater was blinded to group allocation.
	4.3 Were outcome assessors aware of the intervention received by study participants?	N	The rater was blinded to group allocation.
	4.4 If Y/PY/NI to 4.3: Could assessment of the outcome have been influenced by knowledge of intervention received?	NA	
	4.5 If Y/PY/NI to 4.4: Is it likely that assessment of the outcome was influenced by knowledge of intervention received?	NA	
	**Risk of bias judgement**	**Low**	
**Bias in selection of the reported result**	5.1 Were the data that produced this result analysed in accordance with a pre-specified analysis plan that was finalized before unblinded outcome data were available for analysis?	PY	This study was a randomized, single-blind, controlled trial. This [illegible]
	5.2 ... multiple eligible outcome measurements (e.g. scales, definitions, time points) within the outcome domain?	N	TUG, Cooper, Tinetti, OLS, MFS
	5.3 ... multiple eligible analyses of the data?	N	
	**Risk of bias judgement**	**Low**	
**Overall bias**	**Risk of bias judgement**	**Low**	

**Unique ID**	Sinaki 2002	**Study ID**	Sinaki 2002	**Assessor**	Chen
**Ref or Label**		**Aim**	assignment to intervention (the 'intention-to-treat' effect)		
**Experimental**	ST	**Comparator**	Exercise therapy	**Source**	Journal article(s); Trial protocol; Non-commercial trial registry record (e.g. ClinicalTrials.gov record)
**Outcome**	CDP	**Results**		**Weight**	1

Domain	Signalling question	Response	Comments
**Bias arising from the randomization process**	1.1 Was the allocation sequence random?	Y	The subjects were randomly assigned to one of two groups.
	1.2 Was the allocation sequence concealed until participants were enrolled and assigned to interventions?	NI	
	1.3 Did baseline differences between intervention groups suggest a problem with the randomization process?	N	
	**Risk of bias judgement**	**Some concerns**	
**Bias due to deviations from intended interventions**	2.1.Were participants aware of their assigned intervention during the trial?	Y	The other group received the same exer cise program plus PDP training with the use of a 2-pound weighted kypho orthosis
	2.2.Were carers and people delivering the interventions aware of participants' assigned intervention during the trial?	Y	
	2.3. If Y/PY/NI to 2.1 or 2.2: Were there deviations from the intended intervention that arose because of the experimental context?	N	
	2.4 If Y/PY to 2.3: Were these deviations likely to have affected the outcome?	NA	
	2.5. If Y/PY/NI to 2.4: Were these deviations from intended intervention balanced between groups?	NA	
	2.6 Was an appropriate analysis used to estimate the effect of assignment to intervention?	N	
	2.7 If N/PN/NI to 2.6: Was there potential for a substantial impact (on the result) of the failure to analyse participants in the group to which they were randomized?	Y	
	**Risk of bias judgement**	**High**	
**Bias due to missing outcome data**	3.1 Were data for this outcome available for all, or nearly all, participants randomized?	Y	subtests 1-7
	3.2 If N/PN/NI to 3.1: Is there evidence that result was not biased by missing outcome data?	NA	
	3.3 If N/PN to 3.2: Could missingness in the outcome depend on its true value?	NA	
	3.4 If Y/PY/NI to 3.3: Is it likely that missingness in the outcome depended on its true value?	NA	
	**Risk of bias judgement**	**Low**	
**Bias in measurement of the outcome**	4.1 Was the method of measuring the outcome inappropriate?	N	CDP
	4.2 Could measurement or ascertainment of the outcome have differed between intervention groups?	N	The clinician performing CDP was blind to the study group assignment of the subjects
	4.3 Were outcome assessors aware of the intervention received by study participants?	N	The clinician performing CDP was blind to the study group assignment of the subjects
	4.4 If Y/PY/NI to 4.3: Could assessment of the outcome have been influenced by knowledge of intervention received?	NA	
	4.5 If Y/PY/NI to 4.4: Is it likely that assessment of the outcome was influenced by knowledge of intervention received?	NA	
	**Risk of bias judgement**	**Low**	
**Bias in selection of the reported result**	5.1 Were the data that produced this result analysed in accordance with a pre-specified analysis plan that was finalized before unblinded outcome data were available for analysis?	NI	
	5.2 ... multiple eligible outcome measurements (e.g. scales, definitions, time points) within the outcome domain?	Y	
	5.3 ... multiple eligible analyses of the data?	Y	
	**Risk of bias judgement**	**High**	
**Overall bias**	**Risk of bias judgement**	**High**	

**Unique ID**	Stolzenberg 2013	**Study ID**	Stolzenberg 2013	**Assessor**	Chen
**Ref or Label**		**Aim**	assignment to intervention (the 'intention-to-treat' effect)		
**Experimental**	WBVT	**Comparator**	BT	**Source**	Journal article(s); Trial protocol; Non-commercial trial registry record (e.g. ClinicalTrials.gov record)
**Outcome**	RST STST TST OLST	**Results**		**Weight**	1

Domain	Signalling question	Response	Comments
**Bias arising from the randomization process**	1.1 Was the allocation sequence random?	Y	Sixty-eight otherwise healthy post-menopausal women with reduced bone density took part in a nine month intervention randomized into either a whole-body vibration
	1.2 Was the allocation sequence concealed until participants were enrolled and assigned to interventions?	NI	
	1.3 Did baseline differences between intervention groups suggest a problem with the randomization process?	N	There were neither significant differences between groups at baseline nor within or [illegible]
	**Risk of bias judgement**	**Some concerns**	
**Bias due to deviations from intended interventions**	2.1.Were participants aware of their assigned intervention during the trial?	Y	The lead author performed all exercise sessions with the subjects
	2.2.Were carers and people delivering the interventions aware of participants' assigned intervention during the trial?	Y	
	2.3. If Y/PY/NI to 2.1 or 2.2: Were there deviations from the intended intervention that arose because of the experimental context?	N	VIB 91.1%、BAL 90.5%
	2.4 If Y/PY to 2.3: Were these deviations likely to have affected the outcome?	NA	
	2.5. If Y/PY/NI to 2.4: Were these deviations from intended intervention balanced between groups?	NA	
	2.6 Was an appropriate analysis used to estimate the effect of assignment to intervention?	Y	Linear mixed-effects models [16] were used to model time-point (Posturomed: [illegible]
	2.7 If N/PN/NI to 2.6: Was there potential for a substantial impact (on the result) of the failure to analyse participants in the group to which they were randomized?	NA	
	**Risk of bias judgement**	**Low**	
**Bias due to missing outcome data**	3.1 Were data for this outcome available for all, or nearly all, participants randomized?	Y	57 subjects finished the study .Subjects were required to complete each training session [illegible]
	3.2 If N/PN/NI to 3.1: Is there evidence that result was not biased by missing outcome data?	NA	
	3.3 If N/PN to 3.2: Could missingness in the outcome depend on its true value?	NA	
	3.4 If Y/PY/NI to 3.3: Is it likely that missingness in the outcome depended on its true value?	NA	
	**Risk of bias judgement**	**Low**	
**Bias in measurement of the outcome**	4.1 Was the method of measuring the outcome inappropriate?	N	The Posturomed 202 (60 [illegible] 60 cm, Haider Bioswing, Pullenreuth, Germany, [illegible]
	4.2 Could measurement or ascertainment of the outcome have differed between intervention groups?	N	The Posturomed 202 (60 [illegible] 60 cm, Haider Bioswing, Pullenreuth, Germany, [illegible]
	4.3 Were outcome assessors aware of the intervention received by study participants?	NI	
	4.4 If Y/PY/NI to 4.3: Could assessment of the outcome have been influenced by knowledge of intervention received?	PN	
	4.5 If Y/PY/NI to 4.4: Is it likely that assessment of the outcome was influenced by knowledge of intervention received?	NA	
	**Risk of bias judgement**	**Low**	
**Bias in selection of the reported result**	5.1 Were the data that produced this result analysed in accordance with a pre-specified analysis plan that was finalized before unblinded outcome data were available for analysis?	PY	
	5.2 ... multiple eligible outcome measurements (e.g. scales, definitions, time points) within the outcome domain?	PN	RST STST TST OLST
	5.3 ... multiple eligible analyses of the data?	PN	LMM
	**Risk of bias judgement**	**Low**	
**Overall bias**	**Risk of bias judgement**	**Some concerns**	

**Unique ID**	Tseng 2016a	**Study ID**	Tseng 2016a	**Assessor**	Chen
**Ref or Label**		**Aim**	assignment to intervention (the 'intention-to-treat' effect)		
**Experimental**	WBVT	**Comparator**	No training program	**Source**	Journal article(s); Trial protocol; Non-commercial trial registry record (e.g. ClinicalTrials.gov record)
**Outcome**	LOST SRT	**Results**		**Weight**	1

Domain	Signalling question	Response	Comments
**Bias arising from the randomization process**	1.1 Was the allocation sequence random?	Y	The subjects were randomly divided into three groups.
	1.2 Was the allocation sequence concealed until participants were enrolled and assigned to interventions?	Y	We prepared 45 sealed envelopes containing randomized team number in advance.
	1.3 Did baseline differences between intervention groups suggest a problem with the randomization process?	N	There were no statistically significant differences among the three groups in age, [illegible]
	**Risk of bias judgement**	**Low**	
**Bias due to deviations from intended interventions**	2.1.Were participants aware of their assigned intervention during the trial?	Y	The subjects were randomly divided into three groups: a high-frequency vibration group (40 Hz, n = 15), a low frequency vibration group (20 Hz, n
	2.2.Were carers and people delivering the interventions aware of participants' assigned intervention during the trial?	Y	
	2.3. If Y/PY/NI to 2.1 or 2.2: Were there deviations from the intended intervention that arose because of the experimental context?	N	All of the subjects maintained their regular living habits at other times and did not receive [illegible]
	2.4 If Y/PY to 2.3: Were these deviations likely to have affected the outcome?	NA	
	2.5. If Y/PY/NI to 2.4: Were these deviations from intended intervention balanced between groups?	NA	
	2.6 Was an appropriate analysis used to estimate the effect of assignment to intervention?	Y	Repeated measures ANOVA was used Tukey's HSD was used for post hoc analysis.
	2.7 If N/PN/NI to 2.6: Was there potential for a substantial impact (on the result) of the failure to analyse participants in the group to which they were randomized?	NA	
	**Risk of bias judgement**	**Low**	
**Bias due to missing outcome data**	3.1 Were data for this outcome available for all, or nearly all, participants randomized?	Y	The subjects were randomly divided into three groups: a [illegible]
	3.2 If N/PN/NI to 3.1: Is there evidence that result was not biased by missing outcome data?	NA	
	3.3 If N/PN to 3.2: Could missingness in the outcome depend on its true value?	NA	
	3.4 If Y/PY/NI to 3.3: Is it likely that missingness in the outcome depended on its true value?	NA	
	**Risk of bias judgement**	**Low**	
**Bias in measurement of the outcome**	4.1 Was the method of measuring the outcome inappropriate?	N	To measure balance, this study used the limits of stability test of the Balance system [illegible]
	4.2 Could measurement or ascertainment of the outcome have differed between intervention groups?	N	To measure balance, this study used the limits of stability test of the Balance system [illegible]
	4.3 Were outcome assessors aware of the intervention received by study participants?	NI	Researchers responsible for performing assessments had no idea of the randomized [illegible]
	4.4 If Y/PY/NI to 4.3: Could assessment of the outcome have been influenced by knowledge of intervention received?	N	
	4.5 If Y/PY/NI to 4.4: Is it likely that assessment of the outcome was influenced by knowledge of intervention received?	NA	
	**Risk of bias judgement**	**Low**	
**Bias in selection of the reported result**	5.1 Were the data that produced this result analysed in accordance with a pre-specified analysis plan that was finalized before unblinded outcome data were available for analysis?	PY	The complete date range for par ticipant recruitment and follow-up was from Jan-05-[illegible]
	5.2 ... multiple eligible outcome measurements (e.g. scales, definitions, time points) within the outcome domain?	N	Limits of Stability Test ; Sit and Reach Test
	5.3 ... multiple eligible analyses of the data?	N	ANOVA and Tukey analysis
	**Risk of bias judgement**	**Low**	
**Overall bias**	**Risk of bias judgement**	**Low**	

**Unique ID**	Tseng 2016b	**Study ID**	Tseng 2016b	**Assessor**	Chen
**Ref or Label**		**Aim**	assignment to intervention (the 'intention-to-treat' effect)		
**Experimental**	WBVT	**Comparator**	No training program	**Source**	Journal article(s); Trial protocol; Non-commercial trial registry record (e.g. ClinicalTrials.gov record)
**Outcome**	LOST	**Results**		**Weight**	1

Domain	Signalling question	Response	Comments
**Bias arising from the randomization process**	1.1 Was the allocation sequence random?	Y	Thisstudy, a single-blind randomized trial, used the block randomization method, and the evaluator did not know which subjects were randomized to which group.
	1.2 Was the allocation sequence concealed until participants were enrolled and assigned to interventions?	PY	
	1.3 Did baseline differences between intervention groups suggest a problem with the randomization process?	PN	
	**Risk of bias judgement**	**Low**	
**Bias due to deviations from intended interventions**	2.1.Were participants aware of their assigned intervention during the trial?	Y	. Subjects were randomly divided into a WBV with eyes open group, a visual feedback-deprived plus WBV (VFDWBV) group, and a
	2.2.Were carers and people delivering the interventions aware of participants' assigned intervention during the trial?	Y	
	2.3. If Y/PY/NI to 2.1 or 2.2: Were there deviations from the intended intervention that arose because of the experimental context?	N	The 3 groups maintained their original lifestyles and did not participate in other [illegible]
	2.4 If Y/PY to 2.3: Were these deviations likely to have affected the outcome?	NA	
	2.5. If Y/PY/NI to 2.4: Were these deviations from intended intervention balanced between groups?	NA	
	2.6 Was an appropriate analysis used to estimate the effect of assignment to intervention?	Y	Repeated measures ANOVA were used... Scheffe post hoc analysis
	2.7 If N/PN/NI to 2.6: Was there potential for a substantial impact (on the result) of the failure to analyse participants in the group to which they were randomized?	NA	
	**Risk of bias judgement**	**Low**	
**Bias due to missing outcome data**	3.1 Were data for this outcome available for all, or nearly all, participants randomized?	Y	A total of 51 elderly individuals who did not exercise regularly enrolled in the study. After [illegible]
	3.2 If N/PN/NI to 3.1: Is there evidence that result was not biased by missing outcome data?	NA	
	3.3 If N/PN to 3.2: Could missingness in the outcome depend on its true value?	NA	
	3.4 If Y/PY/NI to 3.3: Is it likely that missingness in the outcome depended on its true value?	NA	
	**Risk of bias judgement**	**Low**	
**Bias in measurement of the outcome**	4.1 Was the method of measuring the outcome inappropriate?	N	This study used the Biodex Balance system (Biodex Medical Systems, Inc., Shirley, NY) to [illegible]
	4.2 Could measurement or ascertainment of the outcome have differed between intervention groups?	N	
	4.3 Were outcome assessors aware of the intervention received by study participants?	PN	Evaluator did not know which subjects were randomized to which group.
	4.4 If Y/PY/NI to 4.3: Could assessment of the outcome have been influenced by knowledge of intervention received?	NA	
	4.5 If Y/PY/NI to 4.4: Is it likely that assessment of the outcome was influenced by knowledge of intervention received?	NA	
	**Risk of bias judgement**	**Low**	
**Bias in selection of the reported result**	5.1 Were the data that produced this result analysed in accordance with a pre-specified analysis plan that was finalized before unblinded outcome data were available for analysis?	PY	The complete date range for participant [illegible]
	5.2 ... multiple eligible outcome measurements (e.g. scales, definitions, time points) within the outcome domain?	N	LOST
	5.3 ... multiple eligible analyses of the data?	N	ANOVA and Scheffe Analysis
	**Risk of bias judgement**	**Low**	
**Overall bias**	**Risk of bias judgement**	**Low**	

**Unique ID**	Sievänen 2024	**Study ID**	Sievänen 2024	**Assessor**	Chen
**Ref or Label**		**Aim**	assignment to intervention (the 'intention-to-treat' effect)		
**Experimental**	WBVT	**Comparator**	Wellness	**Source**	Journal article(s); Trial protocol; Non-commercial trial registry record (e.g. ClinicalTrials.gov record)
**Outcome**	SPPB TUG 5t-CST GT	**Results**		**Weight**	1

Domain	Signalling question	Response	Comments
**Bias arising from the randomization process**	1.1 Was the allocation sequence random?	Y	Participants were randomly allocated into WBV training (n = 68) or wellness training (n = 62) groups in stratified blocks. Performed the stratified randomization for
	1.2 Was the allocation sequence concealed until participants were enrolled and assigned to interventions?	Y	
	1.3 Did baseline differences between intervention groups suggest a problem with the randomization process?	N	
	**Risk of bias judgement**	**Low**	
**Bias due to deviations from intended interventions**	2.1.Were participants aware of their assigned intervention during the trial?	N	Participants were informed that two different training programs were compared while they were kept unaware of the primary study hypothesis.
	2.2.Were carers and people delivering the interventions aware of participants' assigned intervention during the trial?	Y	
	2.3. If Y/PY/NI to 2.1 or 2.2: Were there deviations from the intended intervention that arose because of the experimental context?	N	The supervisors recorded both the attendance and deviations from the intended training [illegible]
	2.4 If Y/PY to 2.3: Were these deviations likely to have affected the outcome?	NA	
	2.5. If Y/PY/NI to 2.4: Were these deviations from intended intervention balanced between groups?	NA	
	2.6 Was an appropriate analysis used to estimate the effect of assignment to intervention?	Y	All prospectively collected data were analyzed on an intention-to-treat basis.Generalized [illegible]
	2.7 If N/PN/NI to 2.6: Was there potential for a substantial impact (on the result) of the failure to analyse participants in the group to which they were randomized?	NA	
	**Risk of bias judgement**	**Low**	
**Bias due to missing outcome data**	3.1 Were data for this outcome available for all, or nearly all, participants randomized?	Y	Overall retention rate was 93%. Among those 120 participants who attended [illegible]
	3.2 If N/PN/NI to 3.1: Is there evidence that result was not biased by missing outcome data?	NA	
	3.3 If N/PN to 3.2: Could missingness in the outcome depend on its true value?	NA	
	3.4 If Y/PY/NI to 3.3: Is it likely that missingness in the outcome depended on its true value?	NA	
	**Risk of bias judgement**	**Low**	
**Bias in measurement of the outcome**	4.1 Was the method of measuring the outcome inappropriate?	N	Falls were prospectively collected using fall diaries verified by a telephone call. [illegible]
	4.2 Could measurement or ascertainment of the outcome have differed between intervention groups?	N	
	4.3 Were outcome assessors aware of the intervention received by study participants?	N	The assessors of the outcome variables were blinded to the group allocation, and they were [illegible]
	4.4 If Y/PY/NI to 4.3: Could assessment of the outcome have been influenced by knowledge of intervention received?	NA	
	4.5 If Y/PY/NI to 4.4: Is it likely that assessment of the outcome was influenced by knowledge of intervention received?	NA	
	**Risk of bias judgement**	**Low**	
**Bias in selection of the reported result**	5.1 Were the data that produced this result analysed in accordance with a pre-specified analysis plan that was finalized before unblinded outcome data were available for analysis?	PY	The study protocol has been registered on ClinicalTrials.gov (NCT01523600)This report [illegible]
	5.2 ... multiple eligible outcome measurements (e.g. scales, definitions, time points) within the outcome domain?	N	SPPB;TUG;5t-CST ;5t-CST
	5.3 ... multiple eligible analyses of the data?	N	Intention-to-treat generalized linear mixed models Negative binomial regression
	**Risk of bias judgement**	**Low**	
**Overall bias**	**Risk of bias judgement**	**Low**	

**Unique ID**	Bautmans 2005	**Study ID**	Bautmans 2005	**Assessor**	Chen
**Ref or Label**		**Aim**	assignment to intervention (the 'intention-to-treat' effect)		
**Experimental**	WBVT	**Comparator**	BT	**Source**	
**Outcome**	TUG GT 30s-CST	**Results**		**Weight**	1

Domain	Signalling question	Response	Comments
**Bias arising from the randomization process**	1.1 Was the allocation sequence random?	Y	Randomisation was done for all 24 participants together at the same moment by lottery. From each basket separately, alternatively
	1.2 Was the allocation sequence concealed until participants were enrolled and assigned to interventions?	Y	
	1.3 Did baseline differences between intervention groups suggest a problem with the randomization process?	N	At baseline, WBV+ and control groups were similar for all outcome variables.
	**Risk of bias judgement**	**Low**	
**Bias due to deviations from intended interventions**	2.1.Were participants aware of their assigned intervention during the trial?	N	The sound of the motor was reproduced by a tape recorder thus all subjects were convinced the vibration platform was functioning.
	2.2.Were carers and people delivering the interventions aware of participants' assigned intervention during the trial?	Y	
	2.3. If Y/PY/NI to 2.1 or 2.2: Were there deviations from the intended intervention that arose because of the experimental context?	N	
	2.4 If Y/PY to 2.3: Were these deviations likely to have affected the outcome?	NA	
	2.5. If Y/PY/NI to 2.4: Were these deviations from intended intervention balanced between groups?	NA	
	2.6 Was an appropriate analysis used to estimate the effect of assignment to intervention?	Y	Wilcoxon Signed Ranks Test and Mann-Whitney U Test Worst Rank Score & Last [illegible]
	2.7 If N/PN/NI to 2.6: Was there potential for a substantial impact (on the result) of the failure to analyse participants in the group to which they were randomized?	NA	
	**Risk of bias judgement**	**Low**	
**Bias due to missing outcome data**	3.1 Were data for this outcome available for all, or nearly all, participants randomized?	Y	1 of 24 participants completed the 6-week program attended respectively 96% and 86% [illegible]
	3.2 If N/PN/NI to 3.1: Is there evidence that result was not biased by missing outcome data?	NA	
	3.3 If N/PN to 3.2: Could missingness in the outcome depend on its true value?	NA	
	3.4 If Y/PY/NI to 3.3: Is it likely that missingness in the outcome depended on its true value?	NA	
	**Risk of bias judgement**	**Low**	
**Bias in measurement of the outcome**	4.1 Was the method of measuring the outcome inappropriate?	N	Tinetti test、Timed Up and Go;Grip strength;Leg extension
	4.2 Could measurement or ascertainment of the outcome have differed between intervention groups?	N	Functional performance assessment was done by a physical therapist who was [illegible]
	4.3 Were outcome assessors aware of the intervention received by study participants?	N	The sound of the motor was reproduced by a tape recorder thus all subjects were convinced [illegible]
	4.4 If Y/PY/NI to 4.3: Could assessment of the outcome have been influenced by knowledge of intervention received?	NA	
	4.5 If Y/PY/NI to 4.4: Is it likely that assessment of the outcome was influenced by knowledge of intervention received?	NA	
	**Risk of bias judgement**	**Low**	
**Bias in selection of the reported result**	5.1 Were the data that produced this result analysed in accordance with a pre-specified analysis plan that was finalized before unblinded outcome data were available for analysis?	PY	
	5.2 ... multiple eligible outcome measurements (e.g. scales, definitions, time points) within the outcome domain?	N	Only Measurements
	5.3 ... multiple eligible analyses of the data?	N	Wilcoxon Signed Ranks Test and Mann-Whitney U Test
	**Risk of bias judgement**	**Low**	
**Overall bias**	**Risk of bias judgement**	**Low**	

**Unique ID**	Bogaerts 2007	**Study ID**	Bogaerts 2007	**Assessor**	Chen
**Ref or Label**		**Aim**	assignment to intervention (the 'intention-to-treat' effect)		
**Experimental**	WBVT	**Comparator**	Fitness	**Source**	Journal article(s); Trial protocol; Non-commercial trial registry record (e.g. ClinicalTrials.gov record)
**Outcome**	SOT	**Results**		**Weight**	1

Domain	Signalling question	Response	Comments
**Bias arising from the randomization process**	1.1 Was the allocation sequence random?	Y	220 men and women were randomly assigned to one of three groups
	1.2 Was the allocation sequence concealed until participants were enrolled and assigned to interventions?	NI	
	1.3 Did baseline differences between intervention groups suggest a problem with the randomization process?	N	no significant differences at baseline were found between the groups in age, body mass, [illegible]
	**Risk of bias judgement**	**Some concerns**	
**Bias due to deviations from intended interventions**	2.1.Were participants aware of their assigned intervention during the trial?	PY	220 men and women were randomly assigned to one of three groups: two training groups, the WBV group (n = 94; 46 women, 48 men; 66.8 0.5 years) or the fitness (FIT) group (n
	2.2.Were carers and people delivering the interventions aware of participants' assigned intervention during the trial?	Y	
	2.3. If Y/PY/NI to 2.1 or 2.2: Were there deviations from the intended intervention that arose because of the experimental context?	N	only data of participants with ≥80% compliance were analyzed
	2.4 If Y/PY to 2.3: Were these deviations likely to have affected the outcome?	NA	
	2.5. If Y/PY/NI to 2.4: Were these deviations from intended intervention balanced between groups?	NA	
	2.6 Was an appropriate analysis used to estimate the effect of assignment to intervention?	PY	One-way analysis of variance (ANOVA) was used to testfor differences between the [illegible]
	2.7 If N/PN/NI to 2.6: Was there potential for a substantial impact (on the result) of the failure to analyse participants in the group to which they were randomized?	NA	
	**Risk of bias judgement**	**Low**	
**Bias due to missing outcome data**	3.1 Were data for this outcome available for all, or nearly all, participants randomized?	Y	220 men and women were randomly assigned to one of three groups: two training groups, [illegible]
	3.2 If N/PN/NI to 3.1: Is there evidence that result was not biased by missing outcome data?	NA	
	3.3 If N/PN to 3.2: Could missingness in the outcome depend on its true value?	NA	
	3.4 If Y/PY/NI to 3.3: Is it likely that missingness in the outcome depended on its true value?	NA	
	**Risk of bias judgement**	**Low**	
**Bias in measurement of the outcome**	4.1 Was the method of measuring the outcome inappropriate?	N	SOT、MCT、ADT
	4.2 Could measurement or ascertainment of the outcome have differed between intervention groups?	N	
	4.3 Were outcome assessors aware of the intervention received by study participants?	NI	
	4.4 If Y/PY/NI to 4.3: Could assessment of the outcome have been influenced by knowledge of intervention received?	PN	
	4.5 If Y/PY/NI to 4.4: Is it likely that assessment of the outcome was influenced by knowledge of intervention received?	NA	
	**Risk of bias judgement**	**Low**	
**Bias in selection of the reported result**	5.1 Were the data that produced this result analysed in accordance with a pre-specified analysis plan that was finalized before unblinded outcome data were available for analysis?	NI	
	5.2 ... multiple eligible outcome measurements (e.g. scales, definitions, time points) within the outcome domain?	N	SOT、MCT、ADT
	5.3 ... multiple eligible analyses of the data?	N	Chi-square Test; ANOVA analysis;
	**Risk of bias judgement**	**Some concerns**	
**Overall bias**	**Risk of bias judgement**	**Some concerns**	

**Unique ID**	Nawrat-Szołtysik 2022	**Study ID**	Nawrat-Szołtysik 2022	**Assessor**	Chen
**Ref or Label**		**Aim**	assignment to intervention (the 'intention-to-treat' effect)		
**Experimental**	WBVT	**Comparator**	No training program	**Source**	Journal article(s); Trial protocol; Non-commercial trial registry record (e.g. ClinicalTrials.gov record)
**Outcome**	TUG 6m-WT 30s-CST FES-I	**Results**		**Weight**	1

Domain	Signalling question	Response	Comments
**Bias arising from the randomization process**	1.1 Was the allocation sequence random?	Y	The women were randomly allocated to two groups. 46 opaque envelopes... marked with letters A (control) or B (experimental)placed in
	1.2 Was the allocation sequence concealed until participants were enrolled and assigned to interventions?	Y	
	1.3 Did baseline differences between intervention groups suggest a problem with the randomization process?	N	All tests showed that none of the variables significantly differentiated patients in the [illegible]
	**Risk of bias judgement**	**Low**	
**Bias due to deviations from intended interventions**	2.1.Were participants aware of their assigned intervention during the trial?	Y	All sessions were supervised by the same physiotherapist.
	2.2.Were carers and people delivering the interventions aware of participants' assigned intervention during the trial?	PY	
	2.3. If Y/PY/NI to 2.1 or 2.2: Were there deviations from the intended intervention that arose because of the experimental context?	PN	
	2.4 If Y/PY to 2.3: Were these deviations likely to have affected the outcome?	NA	
	2.5. If Y/PY/NI to 2.4: Were these deviations from intended intervention balanced between groups?	NA	
	2.6 Was an appropriate analysis used to estimate the effect of assignment to intervention?	Y	Statistical analysis... Wilcoxon test, Mann–Whitney U test... for non-parametric data
	2.7 If N/PN/NI to 2.6: Was there potential for a substantial impact (on the result) of the failure to analyse participants in the group to which they were randomized?	NA	
	**Risk of bias judgement**	**Low**	
**Bias due to missing outcome data**	3.1 Were data for this outcome available for all, or nearly all, participants randomized?	Y	Of those, four women (8.7%) did not complete the study Statistical analysis was performed [illegible]
	3.2 If N/PN/NI to 3.1: Is there evidence that result was not biased by missing outcome data?	NA	
	3.3 If N/PN to 3.2: Could missingness in the outcome depend on its true value?	NA	
	3.4 If Y/PY/NI to 3.3: Is it likely that missingness in the outcome depended on its true value?	NA	
	**Risk of bias judgement**	**Low**	
**Bias in measurement of the outcome**	4.1 Was the method of measuring the outcome inappropriate?	N	TUG、6MWT、30sCST
	4.2 Could measurement or ascertainment of the outcome have differed between intervention groups?	N	
	4.3 Were outcome assessors aware of the intervention received by study participants?	N	Blinding was applied to the person in charge of functional tests, and the statistician.
	4.4 If Y/PY/NI to 4.3: Could assessment of the outcome have been influenced by knowledge of intervention received?	NA	
	4.5 If Y/PY/NI to 4.4: Is it likely that assessment of the outcome was influenced by knowledge of intervention received?	NA	
	**Risk of bias judgement**	**Low**	
**Bias in selection of the reported result**	5.1 Were the data that produced this result analysed in accordance with a pre-specified analysis plan that was finalized before unblinded outcome data were available for analysis?	PY	It was designed as a prospective, randomized, controlled clinical trial to compare [illegible]
	5.2 ... multiple eligible outcome measurements (e.g. scales, definitions, time points) within the outcome domain?	N	
	5.3 ... multiple eligible analyses of the data?	N	
	**Risk of bias judgement**	**Low**	
**Overall bias**	**Risk of bias judgement**	**Low**	

**Unique ID**	Lam 2018	**Study ID**	Lam 2018	**Assessor**	Chen
**Ref or Label**		**Aim**	assignment to intervention (the 'intention-to-treat' effect)		
**Experimental**	IG1: WBVT IG2: BT	**Comparator**	Conventional	**Source**	Journal article(s); Trial protocol; Non-commercial trial registry record (e.g. ClinicalTrials.gov record)
**Outcome**	TUG TUG FTSST 6m-WT	**Results**		**Weight**	1

Domain	Signalling question	Response	Comments
**Bias arising from the randomization process**	1.1 Was the allocation sequence random?	Y	The allocation was completed by an off-site researcher who was not involved in other aspects of the trial, using an online randomization program with a ratio of 1:1:1.
	1.2 Was the allocation sequence concealed until participants were enrolled and assigned to interventions?	Y	
	1.3 Did baseline differences between intervention groups suggest a problem with the randomization process?	N	No between-group differences identified.
	**Risk of bias judgement**	**Low**	
**Bias due to deviations from intended interventions**	2.1.Were participants aware of their assigned intervention during the trial?	Y	Study participants and exercise instructors were not blinded to group assignment.
	2.2.Were carers and people delivering the interventions aware of participants' assigned intervention during the trial?	Y	
	2.3. If Y/PY/NI to 2.1 or 2.2: Were there deviations from the intended intervention that arose because of the experimental context?	N	
	2.4 If Y/PY to 2.3: Were these deviations likely to have affected the outcome?	NA	
	2.5. If Y/PY/NI to 2.4: Were these deviations from intended intervention balanced between groups?	NA	
	2.6 Was an appropriate analysis used to estimate the effect of assignment to intervention?	Y	Intention-to-treat analysis was conducted last observation carried forward method was used.
	2.7 If N/PN/NI to 2.6: Was there potential for a substantial impact (on the result) of the failure to analyse participants in the group to which they were randomized?	NA	
	**Risk of bias judgement**	**Low**	
**Bias due to missing outcome data**	3.1 Were data for this outcome available for all, or nearly all, participants randomized?	Y	Of these, 62 participants completed all assessments
	3.2 If N/PN/NI to 3.1: Is there evidence that result was not biased by missing outcome data?	NA	
	3.3 If N/PN to 3.2: Could missingness in the outcome depend on its true value?	NA	
	3.4 If Y/PY/NI to 3.3: Is it likely that missingness in the outcome depended on its true value?	NA	
	**Risk of bias judgement**	**Low**	
**Bias in measurement of the outcome**	4.1 Was the method of measuring the outcome inappropriate?	N	TUG、BBS、5xSTS、6MWT、ABC
	4.2 Could measurement or ascertainment of the outcome have differed between intervention groups?	N	
	4.3 Were outcome assessors aware of the intervention received by study participants?	N	Two blinded assessors performed outcome assessments
	4.4 If Y/PY/NI to 4.3: Could assessment of the outcome have been influenced by knowledge of intervention received?	NA	
	4.5 If Y/PY/NI to 4.4: Is it likely that assessment of the outcome was influenced by knowledge of intervention received?	NA	
	**Risk of bias judgement**	**Low**	
**Bias in selection of the reported result**	5.1 Were the data that produced this result analysed in accordance with a pre-specified analysis plan that was finalized before unblinded outcome data were available for analysis?	PY	This study is registered on ClinicalTrials.gov (identifier: NCT01735682).
	5.2 ... multiple eligible outcome measurements (e.g. scales, definitions, time points) within the outcome domain?	PN	TUG、BBS、PPA、ABC、6MWT
	5.3 ... multiple eligible analyses of the data?	N	MANOVA
	**Risk of bias judgement**	**Low**	
**Overall bias**	**Risk of bias judgement**	**Low**	

**Unique ID**	Goudarzian 2017	**Study ID**	Goudarzian 2017	**Assessor**	Chen
**Ref or Label**		**Aim**	assignment to intervention (the 'intention-to-treat' effect)		
**Experimental**	WBV + placebo	**Comparator**	No training program	**Source**	Journal article(s); Trial protocol; Non-commercial trial registry record (e.g. ClinicalTrials.gov record)
**Outcome**	FBT TUG GT 30m-WT	**Results**		**Weight**	1

Domain	Signalling question	Response	Comments
**Bias arising from the randomization process**	1.1 Was the allocation sequence random?	Y	The participants were randomly assigned into one of the following groups
	1.2 Was the allocation sequence concealed until participants were enrolled and assigned to interventions?	PY	
	1.3 Did baseline differences between intervention groups suggest a problem with the randomization process?	N	There were no significant differences (P > 0.05) between the groups among any [illegible]
	**Risk of bias judgement**	**Low**	
**Bias due to deviations from intended interventions**	2.1.Were participants aware of their assigned intervention during the trial?	Y	Physical fitness professionals supervised
	2.2.Were carers and people delivering the interventions aware of participants' assigned intervention during the trial?	Y	
	2.3. If Y/PY/NI to 2.1 or 2.2: Were there deviations from the intended intervention that arose because of the experimental context?	N	The overall average allegiance was 97.8%
	2.4 If Y/PY to 2.3: Were these deviations likely to have affected the outcome?	NA	
	2.5. If Y/PY/NI to 2.4: Were these deviations from intended intervention balanced between groups?	NA	
	2.6 Was an appropriate analysis used to estimate the effect of assignment to intervention?	Y	ANCOVA, one-way ANOVA,Tukey and Sidak post hoc test using SPSS.
	2.7 If N/PN/NI to 2.6: Was there potential for a substantial impact (on the result) of the failure to analyse participants in the group to which they were randomized?	NA	
	**Risk of bias judgement**	**Low**	
**Bias due to missing outcome data**	3.1 Were data for this outcome available for all, or nearly all, participants randomized?	Y	The participants were randomly assigned into 1 of the [illegible]
	3.2 If N/PN/NI to 3.1: Is there evidence that result was not biased by missing outcome data?	NA	
	3.3 If N/PN to 3.2: Could missingness in the outcome depend on its true value?	NA	
	3.4 If Y/PY/NI to 3.3: Is it likely that missingness in the outcome depended on its true value?	NA	
	**Risk of bias judgement**	**Low**	
**Bias in measurement of the outcome**	4.1 Was the method of measuring the outcome inappropriate?	N	TUG、Flamingo Balance Test、30-meter walking、tandem gait
	4.2 Could measurement or ascertainment of the outcome have differed between intervention groups?	N	same research assistant, who was blinded to grouping
	4.3 Were outcome assessors aware of the intervention received by study participants?	N	who was blinded to grouping of the participants and the aims of the study.
	4.4 If Y/PY/NI to 4.3: Could assessment of the outcome have been influenced by knowledge of intervention received?	NA	
	4.5 If Y/PY/NI to 4.4: Is it likely that assessment of the outcome was influenced by knowledge of intervention received?	NA	
	**Risk of bias judgement**	**Low**	
**Bias in selection of the reported result**	5.1 Were the data that produced this result analysed in accordance with a pre-specified analysis plan that was finalized before unblinded outcome data were available for analysis?	PY	
	5.2 ... multiple eligible outcome measurements (e.g. scales, definitions, time points) within the outcome domain?	PN	
	5.3 ... multiple eligible analyses of the data?	N	ANOVA/ANCOVA
	**Risk of bias judgement**	**Low**	
**Overall bias**	**Risk of bias judgement**	**Low**	

Unique ID	Bogaerts 2011	Study ID	Bogaerts 2011	Assessor	Chen
Ref or Label		Aim	assignment to intervention (the 'intention-to-treat' effect)		
Experimental	WBVT	Comparator	No training program	Source	Journal article(s); Trial protocol; Non-commercial trial registry record (e.g. ClinicalTrials.gov record)
Outcome	SOT 10m-WT TUG	Results		Weight	1

Domain	Signalling question	Response	Comments
Bias arising from the randomization process	1.1 Was the allocation sequence random?	Y	113 subjects were randomly assigned according to a computer-generated randomisation list. a blinded envelope-system performed by a
	1.2 Was the allocation sequence concealed until participants were enrolled and assigned to interventions?	Y	
	1.3 Did baseline differences between intervention groups suggest a problem with the randomization process?	N	No baseline differences were found between the WBV and CON groups (all p > 0.05)
	**Risk of bias judgement**	**Low**	
Bias due to deviations from intended interventions	2.1.Were participants aware of their assigned intervention during the trial?	Y	No baseline differences were found between the WBV and CON groups (all p > 0.05)
	2.2.Were carers and people delivering the interventions aware of participants' assigned intervention during the trial?	Y	
	2.3. If Y/PY/NI to 2.1 or 2.2: Were there deviations from the intended intervention that arose because of the experimental context?	N	
	2.4 If Y/PY to 2.3: Were these deviations likely to have affected the outcome?	NA	
	2.5. If Y/PY/NI to 2.4: Were these deviations from intended intervention balanced between groups?	NA	
	2.6 Was an appropriate analysis used to estimate the effect of assignment to intervention?	Y	Data were analysed by an intention-to-treat analysis.
	2.7 If N/PN/NI to 2.6: Was there potential for a substantial impact (on the result) of the failure to analyse participants in the group to which they were randomized?	NA	
	**Risk of bias judgement**	**Low**	
Bias due to missing outcome data	3.1 Were data for this outcome available for all, or nearly all, participants randomized?	Y	A total of 111 individuals were included in the intention-to-treat analysis... only eight dropped
	3.2 If N/PN/NI to 3.1: Is there evidence that result was not biased by missing outcome data?	NA	
	3.3 If N/PN to 3.2: Could missingness in the outcome depend on its true value?	NA	
	3.4 If Y/PY/NI to 3.3: Is it likely that missingness in the outcome depended on its true value?	NA	
	**Risk of bias judgement**	**Low**	
Bias in measurement of the outcome	4.1 Was the method of measuring the outcome inappropriate?	N	TUG、10MWT、Shuttle Walk、SOT
	4.2 Could measurement or ascertainment of the outcome have differed between intervention groups?	N	
	4.3 Were outcome assessors aware of the intervention received by study participants?	PN	Patients were allocated by a blinded envelope-system
	4.4 If Y/PY/NI to 4.3: Could assessment of the outcome have been influenced by knowledge of intervention received?	NA	
	4.5 If Y/PY/NI to 4.4: Is it likely that assessment of the outcome was influenced by knowledge of intervention received?	NA	
	**Risk of bias judgement**	**Low**	
Bias in selection of the reported result	5.1 Were the data that produced this result analysed in accordance with a pre-specified analysis plan that was finalized before unblinded outcome data were available for analysis?	NI	
	5.2 ... multiple eligible outcome measurements (e.g. scales, definitions, time points) within the outcome domain?	PY	TUG、10MWT、SOT、Shuttle Walk
	5.3 ... multiple eligible analyses of the data?	PN	
	**Risk of bias judgement**	**High**	
Overall bias	**Risk of bias judgement**	**High**	

Unique ID	Zhang 2014	Study ID	Zhang 2014	Assessor	Chen
Ref or Label		Aim	assignment to intervention (the 'intention-to-treat' effect)		
Experimental	WBVT	Comparator	Conventional exercises	Source	Journal article(s); Trial protocol; Non-commercial trial registry record (e.g. ClinicalTrials.gov record)
Outcome	TUG 30s-CST ABC	Results		Weight	1

Domain	Signalling question	Response	Comments
Bias arising from the randomization process	1.1 Was the allocation sequence random?	Y	Randomization was carried out by an independent investigator... using a standard table of random digits produced by SPSS software according to the patients' inclusion
	1.2 Was the allocation sequence concealed until participants were enrolled and assigned to interventions?	Y	
	1.3 Did baseline differences between intervention groups suggest a problem with the randomization process?	N	There was no significant difference between the whole-body vibration group and the control
	**Risk of bias judgement**	**Low**	
Bias due to deviations from intended interventions	2.1.Were participants aware of their assigned intervention during the trial?	Y	An experienced physical therapist supervised all the whole-body vibration administrations.
	2.2.Were carers and people delivering the interventions aware of participants' assigned intervention during the trial?	Y	
	2.3. If Y/PY/NI to 2.1 or 2.2: Were there deviations from the intended intervention that arose because of the experimental context?	N	The training schedule was well accepted 19 of 22 completed.
	2.4 If Y/PY to 2.3: Were these deviations likely to have affected the outcome?	NA	
	2.5. If Y/PY/NI to 2.4: Were these deviations from intended intervention balanced between groups?	NA	
	2.6 Was an appropriate analysis used to estimate the effect of assignment to intervention?	Y	both the intention-to-treat analysis and on-protocol analysis were performed and
	2.7 If N/PN/NI to 2.6: Was there potential for a substantial impact (on the result) of the failure to analyse participants in the group to which they were randomized?	NA	
	**Risk of bias judgement**	**Low**	
Bias due to missing outcome data	3.1 Were data for this outcome available for all, or nearly all, participants randomized?	Y	44 patients were randomly assigned 37 completed all procedures.
	3.2 If N/PN/NI to 3.1: Is there evidence that result was not biased by missing outcome data?	NA	
	3.3 If N/PN to 3.2: Could missingness in the outcome depend on its true value?	NA	
	3.4 If Y/PY/NI to 3.3: Is it likely that missingness in the outcome depended on its true value?	NA	
	**Risk of bias judgement**	**Low**	
Bias in measurement of the outcome	4.1 Was the method of measuring the outcome inappropriate?	N	TUG、30s Chair Stand、ABC
	4.2 Could measurement or ascertainment of the outcome have differed between intervention groups?	N	
	4.3 Were outcome assessors aware of the intervention received by study participants?	N	By the researcher who had been blinded to the group allocation.
	4.4 If Y/PY/NI to 4.3: Could assessment of the outcome have been influenced by knowledge of intervention received?	NA	
	4.5 If Y/PY/NI to 4.4: Is it likely that assessment of the outcome was influenced by knowledge of intervention received?	NA	
	**Risk of bias judgement**	**Low**	
Bias in selection of the reported result	5.1 Were the data that produced this result analysed in accordance with a pre-specified analysis plan that was finalized before unblinded outcome data were available for analysis?	NI	
	5.2 ... multiple eligible outcome measurements (e.g. scales, definitions, time points) within the outcome domain?	PN	TUG、Chair Stand、ABC
	5.3 ... multiple eligible analyses of the data?	PY	ITT and Per-protocol analyses
	**Risk of bias judgement**	**High**	
Overall bias	**Risk of bias judgement**	**High**	

**Unique ID**	Pollock 2012	**Study ID**	Pollock 2012	**Assessor**	Chen
**Ref or Label**		**Aim**	assignment to intervention (the 'intention-to-treat' effect)		
**Experimental**	WBVT + Exercise	**Comparator**	Combined exercise	**Source**	Journal article(s); Trial protocol; Non-commercial trial registry record (e.g. ClinicalTrials.gov record)
**Outcome**	TUG 6m-WT BBS FES-I	**Results**		**Weight**	1

**Domain**	**Signalling question**	**Response**	**Comments**
**Bias arising from the randomization process**	1.1 Was the allocation sequence random?	Y	Each participant was randomized by a senior administrator unrelated to the study, in blocks of 6 using a unique study number. Allocation was communicated to the
	1.2 Was the allocation sequence concealed until participants were enrolled and assigned to interventions?	Y	
	1.3 Did baseline differences between intervention groups suggest a problem with the randomization process?	N	There were no significant differences (P > 0.05) in group characteristics for age, sex or
	**Risk of bias judgement**	**Low**	
**Bias due to deviations from intended interventions**	2.1.Were participants aware of their assigned intervention during the trial?	Y	Whereas participants and physical therapists were inevitably aware of group allocation
	2.2.Were carers and people delivering the interventions aware of participants' assigned intervention during the trial?	Y	
	2.3. If Y/PY/NI to 2.1 or 2.2: Were there deviations from the intended intervention that arose because of the experimental context?	N	
	2.4 If Y/PY to 2.3: Were these deviations likely to have affected the outcome?	NA	
	2.5. If Y/PY/NI to 2.4: Were these deviations from intended intervention balanced between groups?	NA	
	2.6 Was an appropriate analysis used to estimate the effect of assignment to intervention?	Y	The primary endpoint was at eight weeks Mann–Whitney tests, T-tests, ANOVA
	2.7 If N/PN/NI to 2.6: Was there potential for a substantial impact (on the result) of the failure to analyse participants in the group to which they were randomized?	NA	
	**Risk of bias judgement**	**Low**	
**Bias due to missing outcome data**	3.1 Were data for this outcome available for all, or nearly all, participants randomized?	PN	Seventy-seven randomized, 56 (73%) completed 8 weeks assessment; 55%
	3.2 If N/PN/NI to 3.1: Is there evidence that result was not biased by missing outcome data?	N	We choose not to employ a numerical imputation strategy... this was a proof-of-
	3.3 If N/PN to 3.2: Could missingness in the outcome depend on its true value?	PY	
	3.4 If Y/PY/NI to 3.3: Is it likely that missingness in the outcome depended on its true value?	PN	
	**Risk of bias judgement**	**Some concerns**	
**Bias in measurement of the outcome**	4.1 Was the method of measuring the outcome inappropriate?	N	TUG 6m-WT BBS FES-I
	4.2 Could measurement or ascertainment of the outcome have differed between intervention groups?	N	
	4.3 Were outcome assessors aware of the intervention received by study participants?	N	The research team performing assessments and analysis were kept blinded.
	4.4 If Y/PY/NI to 4.3: Could assessment of the outcome have been influenced by knowledge of intervention received?	NA	
	4.5 If Y/PY/NI to 4.4: Is it likely that assessment of the outcome was influenced by knowledge of intervention received?	NA	
	**Risk of bias judgement**	**Low**	
**Bias in selection of the reported result**	5.1 Were the data that produced this result analysed in accordance with a pre-specified analysis plan that was finalized before unblinded outcome data were available for analysis?	Y	This study was registered on the ISRCTN Register (ISRCTN19205068)
	5.2 ... multiple eligible outcome measurements (e.g. scales, definitions, time points) within the outcome domain?	PN	
	5.3 ... multiple eligible analyses of the data?	N	
	**Risk of bias judgement**	**Low**	
**Overall bias**	**Risk of bias judgement**	**Some concerns**	

**Unique ID**	Asahina 2023	**Study ID**	Asahina 2023	**Assessor**	Chen
**Ref or Label**		**Aim**	assignment to intervention (the 'intention-to-treat' effect)		
**Experimental**	WBVT	**Comparator**	No training program	**Source**	Journal article(s); Trial protocol; Non-commercial trial registry record (e.g. ClinicalTrials.gov record)
**Outcome**	TUG OLST OLST	**Results**		**Weight**	1

**Domain**	**Signalling question**	**Response**	**Comments**
**Bias arising from the randomization process**	1.1 Was the allocation sequence random?	Y	Randomization was performed centrally using a computer-generated random number list with a permuted block size of four and was appropriately concealed from
	1.2 Was the allocation sequence concealed until participants were enrolled and assigned to interventions?	Y	
	1.3 Did baseline differences between intervention groups suggest a problem with the randomization process?	N	There were no substantial differences between the two study groups in any baseline
	**Risk of bias judgement**	**Low**	
**Bias due to deviations from intended interventions**	2.1.Were participants aware of their assigned intervention during the trial?	Y	This was a 12-week, open-label, multicenter, randomized controlled trial
	2.2.Were carers and people delivering the interventions aware of participants' assigned intervention during the trial?	Y	
	2.3. If Y/PY/NI to 2.1 or 2.2: Were there deviations from the intended intervention that arose because of the experimental context?	N	42 (86%) completed all training sessions during the 12-week study period as per the
	2.4 If Y/PY to 2.3: Were these deviations likely to have affected the outcome?	NA	
	2.5. If Y/PY/NI to 2.4: Were these deviations from intended intervention balanced between groups?	NA	
	2.6 Was an appropriate analysis used to estimate the effect of assignment to intervention?	Y	The primary analysis was performed based on the intention-to-treat principle
	2.7 If N/PN/NI to 2.6: Was there potential for a substantial impact (on the result) of the failure to analyse participants in the group to which they were randomized?	NA	
	**Risk of bias judgement**	**Low**	
**Bias due to missing outcome data**	3.1 Were data for this outcome available for all, or nearly all, participants randomized?	Y	Six (12%) of 49 patients in WBV group and 3 (6%) in control group dropped out.
	3.2 If N/PN/NI to 3.1: Is there evidence that result was not biased by missing outcome data?	NA	
	3.3 If N/PN to 3.2: Could missingness in the outcome depend on its true value?	NA	
	3.4 If Y/PY/NI to 3.3: Is it likely that missingness in the outcome depended on its true value?	NA	
	**Risk of bias judgement**	**Low**	
**Bias in measurement of the outcome**	4.1 Was the method of measuring the outcome inappropriate?	N	TUG OLST OLST
	4.2 Could measurement or ascertainment of the outcome have differed between intervention groups?	N	We used a cloud-based wearable application, Moff Band
	4.3 Were outcome assessors aware of the intervention received by study participants?	N	researchers who were blinded to the study allocation instructed the participants
	4.4 If Y/PY/NI to 4.3: Could assessment of the outcome have been influenced by knowledge of intervention received?	NA	
	4.5 If Y/PY/NI to 4.4: Is it likely that assessment of the outcome was influenced by knowledge of intervention received?	NA	
	**Risk of bias judgement**	**Low**	
**Bias in selection of the reported result**	5.1 Were the data that produced this result analysed in accordance with a pre-specified analysis plan that was finalized before unblinded outcome data were available for analysis?	Y	This study was registered at ClinicalTrials.gov (NCT04774731)
	5.2 ... multiple eligible outcome measurements (e.g. scales, definitions, time points) within the outcome domain?	N	
	5.3 ... multiple eligible analyses of the data?	N	ITT and on-treatment analyses
	**Risk of bias judgement**	**Low**	
**Overall bias**	**Risk of bias judgement**	**Low**	

**Unique ID**	Ko 2017	**Study ID**	Ko 2017	**Assessor**	Chen
**Ref or Label**		**Aim**	assignment to intervention (the 'intention-to-treat' effect)		
**Experimental**	WBVT	**Comparator**	No training program	**Source**	
**Outcome**	LOST STS	**Results**		**Weight**	1

Domain	Signalling question	Response	Comments
**Bias arising from the randomization process**	1.1 Was the allocation sequence random?	Y	Participants were randomly assigned into the following three groups. a blinded research assistant…draw pieces of paper inscribed with participants' names out of
	1.2 Was the allocation sequence concealed until participants were enrolled and assigned to interventions?	Y	
	1.3 Did baseline differences between intervention groups suggest a problem with the randomization process?	N	No
	**Risk of bias judgement**	**Low**	
**Bias due to deviations from intended interventions**	2.1.Were participants aware of their assigned intervention during the trial?	N	Participants were blinded regarding their group assignment
	2.2.Were carers and people delivering the interventions aware of participants' assigned intervention during the trial?	PY	
	2.3. If Y/PY/NI to 2.1 or 2.2: Were there deviations from the intended intervention that arose because of the experimental context?	N	excellent adherence rate of 85%
	2.4 If Y/PY to 2.3: Were these deviations likely to have affected the outcome?	NA	
	2.5. If Y/PY/NI to 2.4: Were these deviations from intended intervention balanced between groups?	NA	
	2.6 Was an appropriate analysis used to estimate the effect of assignment to intervention?	Y	All analyses were performed using... multivariate analysis of variance
	2.7 If N/PN/NI to 2.6: Was there potential for a substantial impact (on the result) of the failure to analyse participants in the group to which they were randomized?	NA	
	**Risk of bias judgement**	**Low**	
**Bias due to missing outcome data**	3.1 Were data for this outcome available for all, or nearly all, participants randomized?	Y	A total of 29 participants completed all of the follow-up measurements
	3.2 If N/PN/NI to 3.1: Is there evidence that result was not biased by missing outcome data?	NA	
	3.3 If N/PN to 3.2: Could missingness in the outcome depend on its true value?	NA	
	3.4 If Y/PY/NI to 3.3: Is it likely that missingness in the outcome depended on its true value?	NA	
	**Risk of bias judgement**	**Low**	
**Bias in measurement of the outcome**	4.1 Was the method of measuring the outcome inappropriate?	N	SMART Balance Master and Biodex System
	4.2 Could measurement or ascertainment of the outcome have differed between intervention groups?	N	
	4.3 Were outcome assessors aware of the intervention received by study participants?	NI	
	4.4 If Y/PY/NI to 4.3: Could assessment of the outcome have been influenced by knowledge of intervention received?	N	
	4.5 If Y/PY/NI to 4.4: Is it likely that assessment of the outcome was influenced by knowledge of intervention received?	NA	
	**Risk of bias judgement**	**Low**	
**Bias in selection of the reported result**	5.1 Were the data that produced this result analysed in accordance with a pre-specified analysis plan that was finalized before unblinded outcome data were available for analysis?	NI	
	5.2 ... multiple eligible outcome measurements (e.g. scales, definitions, time points) within the outcome domain?	PN	LOST STS
	5.3 ... multiple eligible analyses of the data?	N	
	**Risk of bias judgement**	**Some concerns**	
**Overall bias**	**Risk of bias judgement**	**Some concerns**	

**Unique ID**	Yang 2023	**Study ID**	Yang 2023	**Assessor**	Chen
**Ref or Label**		**Aim**	assignment to intervention (the 'intention-to-treat' effect)		
**Experimental**	WBVT	**Comparator**	WBVT	**Source**	Journal article(s); Trial protocol; Non-commercial trial registry record (e.g. ClinicalTrials.gov record)
**Outcome**	BBS CRT	**Results**		**Weight**	1

Domain	Signalling question	Response	Comments
**Bias arising from the randomization process**	1.1 Was the allocation sequence random?	Y	This study used a two-arm, randomized-controlled, and longitudinal design to examine the effects of vibration training
	1.2 Was the allocation sequence concealed until participants were enrolled and assigned to interventions?	PY	
	1.3 Did baseline differences between intervention groups suggest a problem with the randomization process?	N	between group(p > 0.137)
	**Risk of bias judgement**	**Low**	
**Bias due to deviations from intended interventions**	2.1.Were participants aware of their assigned intervention during the trial?	Y	Participants in the training group underwent an 8-week vibration training course while the control group maintained their regular lifestyle
	2.2.Were carers and people delivering the interventions aware of participants' assigned intervention during the trial?	Y	
	2.3. If Y/PY/NI to 2.1 or 2.2: Were there deviations from the intended intervention that arose because of the experimental context?	N	Participants were closely monitored… no major adverse events were reported
	2.4 If Y/PY to 2.3: Were these deviations likely to have affected the outcome?	NA	
	2.5. If Y/PY/NI to 2.4: Were these deviations from intended intervention balanced between groups?	NA	
	2.6 Was an appropriate analysis used to estimate the effect of assignment to intervention?	N	Due to the pilot nature of this study, the per-protocol analysis was used.
	2.7 If N/PN/NI to 2.6: Was there potential for a substantial impact (on the result) of the failure to analyse participants in the group to which they were randomized?	Y	n=42
	**Risk of bias judgement**	**High**	
**Bias due to missing outcome data**	3.1 Were data for this outcome available for all, or nearly all, participants randomized?	Y	22 participants in the training group and 20 in the control group completed the entire [illegible]
	3.2 If N/PN/NI to 3.1: Is there evidence that result was not biased by missing outcome data?	NA	
	3.3 If N/PN to 3.2: Could missingness in the outcome depend on its true value?	NA	
	3.4 If Y/PY/NI to 3.3: Is it likely that missingness in the outcome depended on its true value?	NA	
	**Risk of bias judgement**	**Low**	
**Bias in measurement of the outcome**	4.1 Was the method of measuring the outcome inappropriate?	N	BBS CRT
	4.2 Could measurement or ascertainment of the outcome have differed between intervention groups?	N	The assessors of fall risk factors were blinded to the group allocation of each participant
	4.3 Were outcome assessors aware of the intervention received by study participants?	N	
	4.4 If Y/PY/NI to 4.3: Could assessment of the outcome have been influenced by knowledge of intervention received?	NA	
	4.5 If Y/PY/NI to 4.4: Is it likely that assessment of the outcome was influenced by knowledge of intervention received?	NA	
	**Risk of bias judgement**	**Low**	
**Bias in selection of the reported result**	5.1 Were the data that produced this result analysed in accordance with a pre-specified analysis plan that was finalized before unblinded outcome data were available for analysis?	NI	
	5.2 ... multiple eligible outcome measurements (e.g. scales, definitions, time points) within the outcome domain?	N	
	5.3 ... multiple eligible analyses of the data?	PN	
	**Risk of bias judgement**	**Some concerns**	
**Overall bias**	**Risk of bias judgement**	**High**	

**Unique ID**	Kang 2024	**Study ID**	Kang 2024	**Assessor**	Chen
**Ref or Label**		**Aim**	assignment to intervention (the 'intention-to-treat' effect)		
**Experimental**	NMT	**Comparator**	Traditional training	**Source**	Journal article(s); Trial protocol; Non-commercial trial registry record (e.g. ClinicalTrials.gov record)
**Outcome**	TUG YBT RST	**Results**		**Weight**	1

Domain	Signalling question	Response	Comments
**Bias arising from the randomization process**	1.1 Was the allocation sequence random?	Y	All participants were selected as those who could attend all 24 sessions without absence, and indeed, all participants attended every session.The recruited participants were
	1.2 Was the allocation sequence concealed until participants were enrolled and assigned to interventions?	PY	
	1.3 Did baseline differences between intervention groups suggest a problem with the randomization process?	N	between group p > 0.05
	**Risk of bias judgement**	**Low**	
**Bias due to deviations from intended interventions**	2.1.Were participants aware of their assigned intervention during the trial?	Y	
	2.2.Were carers and people delivering the interventions aware of participants' assigned intervention during the trial?	Y	
	2.3. If Y/PY/NI to 2.1 or 2.2: Were there deviations from the intended intervention that arose because of the experimental context?	N	
	2.4 If Y/PY to 2.3: Were these deviations likely to have affected the outcome?	NA	
	2.5. If Y/PY/NI to 2.4: Were these deviations from intended intervention balanced between groups?	NA	
	2.6 Was an appropriate analysis used to estimate the effect of assignment to intervention?	Y	
	2.7 If N/PN/NI to 2.6: Was there potential for a substantial impact (on the result) of the failure to analyse participants in the group to which they were randomized?	NA	
	**Risk of bias judgement**	**Low**	
**Bias due to missing outcome data**	3.1 Were data for this outcome available for all, or nearly all, participants randomized?	Y	Had not engaged in any specific physical activity in the six months prior to the study. [illegible]
	3.2 If N/PN/NI to 3.1: Is there evidence that result was not biased by missing outcome data?	NA	
	3.3 If N/PN to 3.2: Could missingness in the outcome depend on its true value?	NA	
	3.4 If Y/PY/NI to 3.3: Is it likely that missingness in the outcome depended on its true value?	NA	
	**Risk of bias judgement**	**Low**	
**Bias in measurement of the outcome**	4.1 Was the method of measuring the outcome inappropriate?	N	TUG YBT RST
	4.2 Could measurement or ascertainment of the outcome have differed between intervention groups?	PN	
	4.3 Were outcome assessors aware of the intervention received by study participants?	NI	
	4.4 If Y/PY/NI to 4.3: Could assessment of the outcome have been influenced by knowledge of intervention received?	PN	TUG YBT RST
	4.5 If Y/PY/NI to 4.4: Is it likely that assessment of the outcome was influenced by knowledge of intervention received?	NA	
	**Risk of bias judgement**	**Low**	
**Bias in selection of the reported result**	5.1 Were the data that produced this result analysed in accordance with a pre-specified analysis plan that was finalized before unblinded outcome data were available for analysis?	NI	
	5.2 ... multiple eligible outcome measurements (e.g. scales, definitions, time points) within the outcome domain?	N	TUG YBT RST
	5.3 ... multiple eligible analyses of the data?	PN	
	**Risk of bias judgement**	**Some concerns**	
**Overall bias**	**Risk of bias judgement**	**Some concerns**	

**Unique ID**	Zarzeczny 2024	**Study ID**	Zarzeczny 2024	**Assessor**	Chen
**Ref or Label**		**Aim**	assignment to intervention (the 'intention-to-treat' effect)		
**Experimental**	NMT	**Comparator**	Traditional training	**Source**	Journal article(s); Trial protocol; Non-commercial trial registry record (e.g. ClinicalTrials.gov record)
**Outcome**	TUG 30s-CST 6m-WT	**Results**		**Weight**	1

Domain	Signalling question	Response	Comments
**Bias arising from the randomization process**	1.1 Was the allocation sequence random?	Y	Following enrollment, women were randomized into a control group (CON; n =9) and an electrical stimulation group (ES; n=10).
	1.2 Was the allocation sequence concealed until participants were enrolled and assigned to interventions?	PY	
	1.3 Did baseline differences between intervention groups suggest a problem with the randomization process?	N	between group P>0.05
	**Risk of bias judgement**	**Low**	
**Bias due to deviations from intended interventions**	2.1.Were participants aware of their assigned intervention during the trial?	Y	NMES was delivered supervised by physical therapists
	2.2.Were carers and people delivering the interventions aware of participants' assigned intervention during the trial?	Y	
	2.3. If Y/PY/NI to 2.1 or 2.2: Were there deviations from the intended intervention that arose because of the experimental context?	N	
	2.4 If Y/PY to 2.3: Were these deviations likely to have affected the outcome?	NA	
	2.5. If Y/PY/NI to 2.4: Were these deviations from intended intervention balanced between groups?	NA	
	2.6 Was an appropriate analysis used to estimate the effect of assignment to intervention?	Y	two-way repeated-measures ANOVA
	2.7 If N/PN/NI to 2.6: Was there potential for a substantial impact (on the result) of the failure to analyse participants in the group to which they were randomized?	NA	
	**Risk of bias judgement**	**Low**	
**Bias due to missing outcome data**	3.1 Were data for this outcome available for all, or nearly all, participants randomized?	Y	Following enrollment, women were randomized into a control group (CON; n =9) [illegible]
	3.2 If N/PN/NI to 3.1: Is there evidence that result was not biased by missing outcome data?	NA	
	3.3 If N/PN to 3.2: Could missingness in the outcome depend on its true value?	NA	
	3.4 If Y/PY/NI to 3.3: Is it likely that missingness in the outcome depended on its true value?	NA	
	**Risk of bias judgement**	**Low**	
**Bias in measurement of the outcome**	4.1 Was the method of measuring the outcome inappropriate?	N	TUG、30sCST、6MWT
	4.2 Could measurement or ascertainment of the outcome have differed between intervention groups?	N	
	4.3 Were outcome assessors aware of the intervention received by study participants?	PY	
	4.4 If Y/PY/NI to 4.3: Could assessment of the outcome have been influenced by knowledge of intervention received?	PN	
	4.5 If Y/PY/NI to 4.4: Is it likely that assessment of the outcome was influenced by knowledge of intervention received?	NA	
	**Risk of bias judgement**	**Low**	
**Bias in selection of the reported result**	5.1 Were the data that produced this result analysed in accordance with a pre-specified analysis plan that was finalized before unblinded outcome data were available for analysis?	PY	
	5.2 ... multiple eligible outcome measurements (e.g. scales, definitions, time points) within the outcome domain?	PN	
	5.3 ... multiple eligible analyses of the data?	PN	
	**Risk of bias judgement**	**Low**	
**Overall bias**	**Risk of bias judgement**	**Low**	

**Unique ID**	Jang 2021	**Study ID**	Jang 2021	**Assessor**	Chen
**Ref or Label**		**Aim**	assignment to intervention (the 'intention-to-treat' effect)		
**Experimental**	NMT	**Comparator**	No training program	**Source**	Journal article(s); Trial protocol; Non-commercial trial registry record (e.g. ClinicalTrials.gov record)
**Outcome**	FTSST TUG TUG YBT	**Results**		**Weight**	1

Domain	Signalling question	Response	Comments
**Bias arising from the randomization process**	1.1 Was the allocation sequence random?	Y	Thirty elderly women aged 65 or above were randomly categorized into three groups
	1.2 Was the allocation sequence concealed until participants were enrolled and assigned to interventions?	PY	
	1.3 Did baseline differences between intervention groups suggest a problem with the randomization process?	N	General characteristics of the participants(p>0.05)
	**Risk of bias judgement**	**Low**	
**Bias due to deviations from intended interventions**	2.1.Were participants aware of their assigned intervention during the trial?	Y	NMT
	2.2.Were carers and people delivering the interventions aware of participants' assigned intervention during the trial?	Y	
	2.3. If Y/PY/NI to 2.1 or 2.2: Were there deviations from the intended intervention that arose because of the experimental context?	N	
	2.4 If Y/PY to 2.3: Were these deviations likely to have affected the outcome?	NA	
	2.5. If Y/PY/NI to 2.4: Were these deviations from intended intervention balanced between groups?	NA	
	2.6 Was an appropriate analysis used to estimate the effect of assignment to intervention?	Y	one-way ANOVA and paired t-tests
	2.7 If N/PN/NI to 2.6: Was there potential for a substantial impact (on the result) of the failure to analyse participants in the group to which they were randomized?	NA	
	**Risk of bias judgement**	**Low**	
**Bias due to missing outcome data**	3.1 Were data for this outcome available for all, or nearly all, participants randomized?	Y	3 subjects were dropped out of the experiment because the subject herself was [illegible]
	3.2 If N/PN/NI to 3.1: Is there evidence that result was not biased by missing outcome data?	NA	
	3.3 If N/PN to 3.2: Could missingness in the outcome depend on its true value?	NA	
	3.4 If Y/PY/NI to 3.3: Is it likely that missingness in the outcome depended on its true value?	NA	
	**Risk of bias judgement**	**Low**	
**Bias in measurement of the outcome**	4.1 Was the method of measuring the outcome inappropriate?	N	TUG、YBT(ICC)
	4.2 Could measurement or ascertainment of the outcome have differed between intervention groups?	N	
	4.3 Were outcome assessors aware of the intervention received by study participants?	NI	
	4.4 If Y/PY/NI to 4.3: Could assessment of the outcome have been influenced by knowledge of intervention received?	N	
	4.5 If Y/PY/NI to 4.4: Is it likely that assessment of the outcome was influenced by knowledge of intervention received?	NA	
	**Risk of bias judgement**	**Low**	
**Bias in selection of the reported result**	5.1 Were the data that produced this result analysed in accordance with a pre-specified analysis plan that was finalized before unblinded outcome data were available for analysis?	PY	
	5.2 ... multiple eligible outcome measurements (e.g. scales, definitions, time points) within the outcome domain?	N	
	5.3 ... multiple eligible analyses of the data?	PN	
	**Risk of bias judgement**	**Low**	
**Overall bias**	**Risk of bias judgement**	**Low**	

**Unique ID**	Mesquita LSA 2015	**Study ID**	Mesquita LSA 2015	**Assessor**	Chen
**Ref or Label**		**Aim**	assignment to intervention (the 'intention-to-treat' effect)		
**Experimental**	NMT	**Comparator**	No training program	**Source**	
**Outcome**	TUG FRT BBS	**Results**		**Weight**	1

Domain	Signalling question	Response	Comments
**Bias arising from the randomization process**	1.1 Was the allocation sequence random?	Y	Each participant was randomly assigned by an independent researcher
	1.2 Was the allocation sequence concealed until participants were enrolled and assigned to interventions?	PY	
	1.3 Did baseline differences between intervention groups suggest a problem with the randomization process?	N	No differences in age, weight, height and body mass index were found between the three [illegible]
	**Risk of bias judgement**	**Low**	
**Bias due to deviations from intended interventions**	2.1.Were participants aware of their assigned intervention during the trial?	Y	Exercises were performed by two physiotherapy professionals. The PNFG provider was certified in PNF, and the PG provider was certified in Pilates.
	2.2.Were carers and people delivering the interventions aware of participants' assigned intervention during the trial?	Y	
	2.3. If Y/PY/NI to 2.1 or 2.2: Were there deviations from the intended intervention that arose because of the experimental context?	N	
	2.4 If Y/PY to 2.3: Were these deviations likely to have affected the outcome?	NA	
	2.5. If Y/PY/NI to 2.4: Were these deviations from intended intervention balanced between groups?	NA	
	2.6 Was an appropriate analysis used to estimate the effect of assignment to intervention?	PY	A per protocol analysis was used PASW Statistics,ANOVA
	2.7 If N/PN/NI to 2.6: Was there potential for a substantial impact (on the result) of the failure to analyse participants in the group to which they were randomized?	NA	
	**Risk of bias judgement**	**Low**	
**Bias due to missing outcome data**	3.1 Were data for this outcome available for all, or nearly all, participants randomized?	Y	Of the 63 women, 58 completed the program.
	3.2 If N/PN/NI to 3.1: Is there evidence that result was not biased by missing outcome data?	NA	
	3.3 If N/PN to 3.2: Could missingness in the outcome depend on its true value?	NA	
	3.4 If Y/PY/NI to 3.3: Is it likely that missingness in the outcome depended on its true value?	NA	
	**Risk of bias judgement**	**Low**	
**Bias in measurement of the outcome**	4.1 Was the method of measuring the outcome inappropriate?	N	TUG FRT BBS
	4.2 Could measurement or ascertainment of the outcome have differed between intervention groups?	N	
	4.3 Were outcome assessors aware of the intervention received by study participants?	N	Data collection and data entry were conducted by re search assistants who were blinded to [illegible]
	4.4 If Y/PY/NI to 4.3: Could assessment of the outcome have been influenced by knowledge of intervention received?	NA	
	4.5 If Y/PY/NI to 4.4: Is it likely that assessment of the outcome was influenced by knowledge of intervention received?	NA	
	**Risk of bias judgement**	**Low**	
**Bias in selection of the reported result**	5.1 Were the data that produced this result analysed in accordance with a pre-specified analysis plan that was finalized before unblinded outcome data were available for analysis?	PY	ClinicalTrials.gov（NCT02278731）
	5.2 ... multiple eligible outcome measurements (e.g. scales, definitions, time points) within the outcome domain?	N	
	5.3 ... multiple eligible analyses of the data?	N	
	**Risk of bias judgement**	**Low**	
**Overall bias**	**Risk of bias judgement**	**Low**	

**Unique ID**	Concha-Cisternas 2023	**Study ID**	Concha-Cisternas 2023	**Assessor**	Chen
**Ref or Label**		**Aim**	assignment to intervention (the 'intention-to-treat' effect)		
**Experimental**	IG1: NMT IG2: BT	**Comparator**	No training program	**Source**	Journal article(s); Trial protocol; Non-commercial trial registry record (e.g. ClinicalTrials.gov record)
**Outcome**	SPPB 6m-WT GT FTSST	**Results**		**Weight**	1

**Domain**	**Signalling question**	**Response**	**Comments**
**Bias arising from the randomization process**	1.1 Was the allocation sequence random?	Y	A research randomization website was used for randomization.
	1.2 Was the allocation sequence concealed until participants were enrolled and assigned to interventions?	Y	To maintain allocation concealment, sequentially numbered, opaque, sealed, and
	1.3 Did baseline differences between intervention groups suggest a problem with the randomization process?	N	Table 1 presents the baseline characteristics showing no significant differences
	**Risk of bias judgement**	**Low**	
**Bias due to deviations from intended interventions**	2.1.Were participants aware of their assigned intervention during the trial?	N	This study corresponds to a double-blind (participants and physical therapists) randomized controlled trial.
	2.2.Were carers and people delivering the interventions aware of participants' assigned intervention during the trial?	N	double-blind (participants and physical
	2.3. If Y/PY/NI to 2.1 or 2.2: Were there deviations from the intended intervention that arose because of the experimental context?	NA	
	2.4 If Y/PY to 2.3: Were these deviations likely to have affected the outcome?	NA	
	2.5. If Y/PY/NI to 2.4: Were these deviations from intended intervention balanced between groups?	NA	
	2.6 Was an appropriate analysis used to estimate the effect of assignment to intervention?	Y	An intention-to-treat (ITT) analysis was conducted using a single imputation with the [illegible]
	2.7 If N/PN/NI to 2.6: Was there potential for a substantial impact (on the result) of the failure to analyse participants in the group to which they were randomized?	NA	
	**Risk of bias judgement**	**Low**	
**Bias due to missing outcome data**	3.1 Were data for this outcome available for all, or nearly all, participants randomized?	Y	Out of the 48 participants initially assessed, 2 were lost to follow-up.
	3.2 If N/PN/NI to 3.1: Is there evidence that result was not biased by missing outcome data?	NA	
	3.3 If N/PN to 3.2: Could missingness in the outcome depend on its true value?	NA	
	3.4 If Y/PY/NI to 3.3: Is it likely that missingness in the outcome depended on its true value?	NA	
	**Risk of bias judgement**	**Low**	
**Bias in measurement of the outcome**	4.1 Was the method of measuring the outcome inappropriate?	N	SPPB 6m-WT GT FTSST
	4.2 Could measurement or ascertainment of the outcome have differed between intervention groups?	N	
	4.3 Were outcome assessors aware of the intervention received by study participants?	Y	the assessors were not blinded
	4.4 If Y/PY/NI to 4.3: Could assessment of the outcome have been influenced by knowledge of intervention received?	PN	
	4.5 If Y/PY/NI to 4.4: Is it likely that assessment of the outcome was influenced by knowledge of intervention received?	NA	
	**Risk of bias judgement**	**Low**	
**Bias in selection of the reported result**	5.1 Were the data that produced this result analysed in accordance with a pre-specified analysis plan that was finalized before unblinded outcome data were available for analysis?	PY	All participants voluntarily read and signed an informed assent while their legal guardians [illegible]
	5.2 ... multiple eligible outcome measurements (e.g. scales, definitions, time points) within the outcome domain?	N	
	5.3 ... multiple eligible analyses of the data?	N	
	**Risk of bias judgement**	**Low**	
**Overall bias**	**Risk of bias judgement**	**Low**	

**Unique ID**	Smaili 2018	**Study ID**	Smaili 2018	**Assessor**	Chen
**Ref or Label**		**Aim**	assignment to intervention (the 'intention-to-treat' effect)		
**Experimental**	NMT	**Comparator**	RT	**Source**	Journal article(s); Trial protocol; Non-commercial trial registry record (e.g. ClinicalTrials.gov record)
**Outcome**	5m-WT GT	**Results**		**Weight**	1

**Domain**	**Signalling question**	**Response**	**Comments**
**Bias arising from the randomization process**	1.1 Was the allocation sequence random?	Y	Random table of numbers was generated using the random sequence generator procedure (from the www.random.org website).
	1.2 Was the allocation sequence concealed until participants were enrolled and assigned to interventions?	Y	
	1.3 Did baseline differences between intervention groups suggest a problem with the randomization process?	N	The groups were homogeneous in the initial assessment in regard to age, weight, height, [illegible]
	**Risk of bias judgement**	**Low**	
**Bias due to deviations from intended interventions**	2.1.Were participants aware of their assigned intervention during the trial?	Y	The patients with PD were not blind in regard to the two types of training implemented in this study.
	2.2.Were carers and people delivering the interventions aware of participants' assigned intervention during the trial?	Y	
	2.3. If Y/PY/NI to 2.1 or 2.2: Were there deviations from the intended intervention that arose because of the experimental context?	Y	did not receive allocated intervention... more than 3 faults in therapy, surgery, change in [illegible]
	2.4 If Y/PY to 2.3: Were these deviations likely to have affected the outcome?	PY	change in dopaminergic medication and fall
	2.5. If Y/PY/NI to 2.4: Were these deviations from intended intervention balanced between groups?	PY	
	2.6 Was an appropriate analysis used to estimate the effect of assignment to intervention?	Y	Analysed in ITT analysis
	2.7 If N/PN/NI to 2.6: Was there potential for a substantial impact (on the result) of the failure to analyse participants in the group to which they were randomized?	NA	
	**Risk of bias judgement**	**Some concerns**	
**Bias due to missing outcome data**	3.1 Were data for this outcome available for all, or nearly all, participants randomized?	PY	
	3.2 If N/PN/NI to 3.1: Is there evidence that result was not biased by missing outcome data?	NA	
	3.3 If N/PN to 3.2: Could missingness in the outcome depend on its true value?	NA	
	3.4 If Y/PY/NI to 3.3: Is it likely that missingness in the outcome depended on its true value?	NA	
	**Risk of bias judgement**	**Some concerns**	The loss rate is 35%.
**Bias in measurement of the outcome**	4.1 Was the method of measuring the outcome inappropriate?	N	video gait analysis footprint test PDQ-39 PDQL
	4.2 Could measurement or ascertainment of the outcome have differed between intervention groups?	N	
	4.3 Were outcome assessors aware of the intervention received by study participants?	N	No physical therapists engaged in the study... were involved in the assessments of gait and [illegible]
	4.4 If Y/PY/NI to 4.3: Could assessment of the outcome have been influenced by knowledge of intervention received?	NA	
	4.5 If Y/PY/NI to 4.4: Is it likely that assessment of the outcome was influenced by knowledge of intervention received?	NA	
	**Risk of bias judgement**	**Low**	
**Bias in selection of the reported result**	5.1 Were the data that produced this result analysed in accordance with a pre-specified analysis plan that was finalized before unblinded outcome data were available for analysis?	PY	This randomized clinical trial was conducted between February and August 2014 at the [illegible]
	5.2 ... multiple eligible outcome measurements (e.g. scales, definitions, time points) within the outcome domain?	PN	
	5.3 ... multiple eligible analyses of the data?	PN	
	**Risk of bias judgement**	**Low**	
**Overall bias**	**Risk of bias judgement**	**Some concerns**	

**Unique ID**	Acheche 2020	**Study ID**	Acheche 2020	**Assessor**	Chen
**Ref or Label**		**Aim**	assignment to intervention (the 'intention-to-treat' effect)		
**Experimental**	NMT	**Comparator**	RT	**Source**	Journal article(s); Trial protocol; Non-commercial trial registry record (e.g. ClinicalTrials.gov record)
**Outcome**	TUG 6m-WT BBS	**Results**		**Weight**	1

Domain	Signalling question	Response	Comments
**Bias arising from the randomization process**	1.1 Was the allocation sequence random?	Y	The patients were assigned randomly... using a computer-generated randomization list
	1.2 Was the allocation sequence concealed until participants were enrolled and assigned to interventions?	PY	
	1.3 Did baseline differences between intervention groups suggest a problem with the randomization process?	N	between group p > 0.05
	**Risk of bias judgement**	**Low**	
**Bias due to deviations from intended interventions**	2.1.Were participants aware of their assigned intervention during the trial?	Y	
	2.2.Were carers and people delivering the interventions aware of participants' assigned intervention during the trial?	Y	
	2.3. If Y/PY/NI to 2.1 or 2.2: Were there deviations from the intended intervention that arose because of the experimental context?	N	
	2.4 If Y/PY to 2.3: Were these deviations likely to have affected the outcome?	NA	
	2.5. If Y/PY/NI to 2.4: Were these deviations from intended intervention balanced between groups?	NA	
	2.6 Was an appropriate analysis used to estimate the effect of assignment to intervention?	N	n=22 vs. n=22，n=20 vs. n=20
	2.7 If N/PN/NI to 2.6: Was there potential for a substantial impact (on the result) of the failure to analyse participants in the group to which they were randomized?	PN	
	**Risk of bias judgement**	**Some concerns**	
**Bias due to missing outcome data**	3.1 Were data for this outcome available for all, or nearly all, participants randomized?	PY	Did not receive allocated intervention(n=7) Total (n=49)
	3.2 If N/PN/NI to 3.1: Is there evidence that result was not biased by missing outcome data?	NA	
	3.3 If N/PN to 3.2: Could missingness in the outcome depend on its true value?	NA	
	3.4 If Y/PY/NI to 3.3: Is it likely that missingness in the outcome depended on its true value?	NA	
	**Risk of bias judgement**	**Low**	
**Bias in measurement of the outcome**	4.1 Was the method of measuring the outcome inappropriate?	N	TUG 6m-WT BBS
	4.2 Could measurement or ascertainment of the outcome have differed between intervention groups?	N	
	4.3 Were outcome assessors aware of the intervention received by study participants?	NI	
	4.4 If Y/PY/NI to 4.3: Could assessment of the outcome have been influenced by knowledge of intervention received?	PN	
	4.5 If Y/PY/NI to 4.4: Is it likely that assessment of the outcome was influenced by knowledge of intervention received?	NA	
	**Risk of bias judgement**	**Low**	
**Bias in selection of the reported result**	5.1 Were the data that produced this result analysed in accordance with a pre-specified analysis plan that was finalized before unblinded outcome data were available for analysis?	Y	They were recruited from the department of physiology and lung function testing of Farhat [illegible]
	5.2 ... multiple eligible outcome measurements (e.g. scales, definitions, time points) within the outcome domain?	N	
	5.3 ... multiple eligible analyses of the data?	N	
	**Risk of bias judgement**	**Low**	
**Overall bias**	**Risk of bias judgement**	**Some concerns**	

**Unique ID**	Yuzlu 2022	**Study ID**	Yuzlu 2022	**Assessor**	Chen
**Ref or Label**		**Aim**	assignment to intervention (the 'intention-to-treat' effect)		
**Experimental**	BT	**Comparator**	Conventional exercises	**Source**	Journal article(s); Trial protocol; Non-commercial trial registry record (e.g. ClinicalTrials.gov record)
**Outcome**	BBS TUG 10m-WT FES	**Results**		**Weight**	1

Domain	Signalling question	Response	Comments
**Bias arising from the randomization process**	1.1 Was the allocation sequence random?	Y	The randomization scheme from http://www.randomization.com was used. Group randomization was performed by an assessor who was blinded to the evaluation of
	1.2 Was the allocation sequence concealed until participants were enrolled and assigned to interventions?	Y	
	1.3 Did baseline differences between intervention groups suggest a problem with the randomization process?	N	Baseline between group (p>0.05)
	**Risk of bias judgement**	**Low**	
**Bias due to deviations from intended interventions**	2.1.Were participants aware of their assigned intervention during the trial?	Y	IDTT,CDTT
	2.2.Were carers and people delivering the interventions aware of participants' assigned intervention during the trial?	Y	
	2.3. If Y/PY/NI to 2.1 or 2.2: Were there deviations from the intended intervention that arose because of the experimental context?	N	
	2.4 If Y/PY to 2.3: Were these deviations likely to have affected the outcome?	NA	
	2.5. If Y/PY/NI to 2.4: Were these deviations from intended intervention balanced between groups?	NA	
	2.6 Was an appropriate analysis used to estimate the effect of assignment to intervention?	Y	repeated-measures ANOVA,multiple imputations were used to adjust to follow-up [illegible]
	2.7 If N/PN/NI to 2.6: Was there potential for a substantial impact (on the result) of the failure to analyse participants in the group to which they were randomized?	NA	
	**Risk of bias judgement**	**Low**	
**Bias due to missing outcome data**	3.1 Were data for this outcome available for all, or nearly all, participants randomized?	Y	randomized (n=58), analysed(n=54) ,lost(6.9%)
	3.2 If N/PN/NI to 3.1: Is there evidence that result was not biased by missing outcome data?	NA	
	3.3 If N/PN to 3.2: Could missingness in the outcome depend on its true value?	NA	
	3.4 If Y/PY/NI to 3.3: Is it likely that missingness in the outcome depended on its true value?	NA	
	**Risk of bias judgement**	**Low**	
**Bias in measurement of the outcome**	4.1 Was the method of measuring the outcome inappropriate?	N	BBS TUG 10m-WT FES
	4.2 Could measurement or ascertainment of the outcome have differed between intervention groups?	N	
	4.3 Were outcome assessors aware of the intervention received by study participants?	N	Assessments were performed before randomization by an assessor who was [illegible]
	4.4 If Y/PY/NI to 4.3: Could assessment of the outcome have been influenced by knowledge of intervention received?	NA	
	4.5 If Y/PY/NI to 4.4: Is it likely that assessment of the outcome was influenced by knowledge of intervention received?	NA	
	**Risk of bias judgement**	**Low**	
**Bias in selection of the reported result**	5.1 Were the data that produced this result analysed in accordance with a pre-specified analysis plan that was finalized before unblinded outcome data were available for analysis?	Y	The study was conducted in accordance with the Helsinki Declaration, and approval was [illegible]
	5.2 ... multiple eligible outcome measurements (e.g. scales, definitions, time points) within the outcome domain?	N	BBS TUG 10m-WT FES
	5.3 ... multiple eligible analyses of the data?	N	ANOVA
	**Risk of bias judgement**	**Low**	
**Overall bias**	**Risk of bias judgement**	**Low**	

Unique ID	Rossi 2014	Study ID	Rossi 2014	Assessor	Chen
Ref or Label		Aim	assignment to intervention (the 'intention-to-treat' effect)		
Experimental	BT	Comparator	No training program	Source	Journal article(s); Trial protocol; Non-commercial trial registry record (e.g. ClinicalTrials.gov record)
Outcome	TUG	Results		Weight	1

Domain	Signalling question	Response	Comments
Bias arising from the randomization process	1.1 Was the allocation sequence random?	Y	The volunteers were randomly divided into 2 groups.<br>participants of each TUG quartile were randomly assigned to either an exercise or
	1.2 Was the allocation sequence concealed until participants were enrolled and assigned to interventions?	PY	
	1.3 Did baseline differences between intervention groups suggest a problem with the randomization process?	N	here were no differences between groups for age, BMI, and the TUG test（Methods)，p > [illegible]
	**Risk of bias judgement**	**Low**	
Bias due to deviations from intended interventions	2.1.Were participants aware of their assigned intervention during the trial?	Y	trampoline、balance board
	2.2.Were carers and people delivering the interventions aware of participants' assigned intervention during the trial?	Y	
	2.3. If Y/PY/NI to 2.1 or 2.2: Were there deviations from the intended intervention that arose because of the experimental context?	N	
	2.4 If Y/PY to 2.3: Were these deviations likely to have affected the outcome?	NA	
	2.5. If Y/PY/NI to 2.4: Were these deviations from intended intervention balanced between groups?	NA	
	2.6 Was an appropriate analysis used to estimate the effect of assignment to intervention?	PY	A 2-way mixed analysis of variance (ANOVA)
	2.7 If N/PN/NI to 2.6: Was there potential for a substantial impact (on the result) of the failure to analyse participants in the group to which they were randomized?	NA	
	**Risk of bias judgement**	**Low**	
Bias due to missing outcome data	3.1 Were data for this outcome available for all, or nearly all, participants randomized?	Y	Resulting in 3 groups of 23 volunteers each.NOT dropped
	3.2 If N/PN/NI to 3.1: Is there evidence that result was not biased by missing outcome data?	NA	
	3.3 If N/PN to 3.2: Could missingness in the outcome depend on its true value?	NA	
	3.4 If Y/PY/NI to 3.3: Is it likely that missingness in the outcome depended on its true value?	NA	
	**Risk of bias judgement**	**Low**	
Bias in measurement of the outcome	4.1 Was the method of measuring the outcome inappropriate?	N	TUG
	4.2 Could measurement or ascertainment of the outcome have differed between intervention groups?	N	
	4.3 Were outcome assessors aware of the intervention received by study participants?	NI	
	4.4 If Y/PY/NI to 4.3: Could assessment of the outcome have been influenced by knowledge of intervention received?	PN	
	4.5 If Y/PY/NI to 4.4: Is it likely that assessment of the outcome was influenced by knowledge of intervention received?	NA	
	**Risk of bias judgement**	**Low**	
Bias in selection of the reported result	5.1 Were the data that produced this result analysed in accordance with a pre-specified analysis plan that was finalized before unblinded outcome data were available for analysis?	Y	This study was approved by the Ethics Committee of State University of Centro-[illegible]
	5.2 ... multiple eligible outcome measurements (e.g. scales, definitions, time points) within the outcome domain?	N	TUG
	5.3 ... multiple eligible analyses of the data?	N	ANOVA
	**Risk of bias judgement**	**Low**	
Overall bias	**Risk of bias judgement**	**Low**	

Unique ID	Halvarsson 2015	Study ID	Halvarsson 2015	Assessor	Chen
Ref or Label		Aim	assignment to intervention (the 'intention-to-treat' effect)		
Experimental	BT	Comparator	No training program	Source	Journal article(s); Trial protocol; Non-commercial trial registry record (e.g. ClinicalTrials.gov record)
Outcome	FES GT OLST	Results		Weight	1

Domain	Signalling question	Response	Comments
Bias arising from the randomization process	1.1 Was the allocation sequence random?	Y	This randomized controlled study (BETA-study; NCT01417598).
	1.2 Was the allocation sequence concealed until participants were enrolled and assigned to interventions?	PY	
	1.3 Did baseline differences between intervention groups suggest a problem with the randomization process?	PN	Baseline p=0.049
	**Risk of bias judgement**	**Low**	
Bias due to deviations from intended interventions	2.1.Were participants aware of their assigned intervention during the trial?	Y	
	2.2.Were carers and people delivering the interventions aware of participants' assigned intervention during the trial?	Y	
	2.3. If Y/PY/NI to 2.1 or 2.2: Were there deviations from the intended intervention that arose because of the experimental context?	N	
	2.4 If Y/PY to 2.3: Were these deviations likely to have affected the outcome?	NA	
	2.5. If Y/PY/NI to 2.4: Were these deviations from intended intervention balanced between groups?	NA	
	2.6 Was an appropriate analysis used to estimate the effect of assignment to intervention?	Y	Analyses were conducted on an intention-to-treat basis (n=96) using a mixed-model [illegible]
	2.7 If N/PN/NI to 2.6: Was there potential for a substantial impact (on the result) of the failure to analyse participants in the group to which they were randomized?	NA	
	**Risk of bias judgement**	**Low**	
Bias due to missing outcome data	3.1 Were data for this outcome available for all, or nearly all, participants randomized?	PN	dropped (n=27) total(n=96)
	3.2 If N/PN/NI to 3.1: Is there evidence that result was not biased by missing outcome data?	PN	
	3.3 If N/PN to 3.2: Could missingness in the outcome depend on its true value?	PN	
	3.4 If Y/PY/NI to 3.3: Is it likely that missingness in the outcome depended on its true value?	NA	
	**Risk of bias judgement**	**Low**	
Bias in measurement of the outcome	4.1 Was the method of measuring the outcome inappropriate?	N	FES GT OLST
	4.2 Could measurement or ascertainment of the outcome have differed between intervention groups?	N	
	4.3 Were outcome assessors aware of the intervention received by study participants?	Y	lack of blinded assessors was a limitation in our previous article
	4.4 If Y/PY/NI to 4.3: Could assessment of the outcome have been influenced by knowledge of intervention received?	PN	
	4.5 If Y/PY/NI to 4.4: Is it likely that assessment of the outcome was influenced by knowledge of intervention received?	NA	
	**Risk of bias judgement**	**Low**	
Bias in selection of the reported result	5.1 Were the data that produced this result analysed in accordance with a pre-specified analysis plan that was finalized before unblinded outcome data were available for analysis?	Y	This randomized controlled study (BETA-study; NCT01417598, ClinicalTrials.gov) [illegible]
	5.2 ... multiple eligible outcome measurements (e.g. scales, definitions, time points) within the outcome domain?	N	FES GT OLST
	5.3 ... multiple eligible analyses of the data?	N	SIDAK post-hoc,ITT,Per-protocol
	**Risk of bias judgement**	**Low**	
Overall bias	**Risk of bias judgement**	**Low**	

**Unique ID**	An 2024	**Study ID**	An 2024	**Assessor**	Chen
**Ref or Label**		**Aim**	assignment to intervention (the 'intention-to-treat' effect)		
**Experimental**	BT	**Comparator**	Conventional exercises	**Source**	Journal article(s); Trial protocol; Non-commercial trial registry record (e.g. ClinicalTrials.gov record)
**Outcome**	TUG 10m-WT	**Results**		**Weight**	1

**Domain**	**Signalling question**	**Response**	**Comments**
**Bias arising from the randomization process**	1.1 Was the allocation sequence random?	Y	he participants were randomly assigned to two groups using the Research Randomizer program (http://www.randomizer.org/).
	1.2 Was the allocation sequence concealed until participants were enrolled and assigned to interventions?	PY	
	1.3 Did baseline differences between intervention groups suggest a problem with the randomization process?	N	baseline p > 0.05
	**Risk of bias judgement**	**Low**	
**Bias due to deviations from intended interventions**	2.1.Were participants aware of their assigned intervention during the trial?	Y	
	2.2.Were carers and people delivering the interventions aware of participants' assigned intervention during the trial?	Y	
	2.3. If Y/PY/NI to 2.1 or 2.2: Were there deviations from the intended intervention that arose because of the experimental context?	N	Five times a week;<br>30 min
	2.4 If Y/PY to 2.3: Were these deviations likely to have affected the outcome?	NA	
	2.5. If Y/PY/NI to 2.4: Were these deviations from intended intervention balanced between groups?	NA	
	2.6 Was an appropriate analysis used to estimate the effect of assignment to intervention?	Y	A two-way analysis of variance (ANOVA) was used to assess the interaction between group [illegible]
	2.7 If N/PN/NI to 2.6: Was there potential for a substantial impact (on the result) of the failure to analyse participants in the group to which they were randomized?	NA	
	**Risk of bias judgement**	**Low**	
**Bias due to missing outcome data**	3.1 Were data for this outcome available for all, or nearly all, participants randomized?	Y	A total of 38 participants were included in the final analysis(total,42);(dropped,4);
	3.2 If N/PN/NI to 3.1: Is there evidence that result was not biased by missing outcome data?	NA	
	3.3 If N/PN to 3.2: Could missingness in the outcome depend on its true value?	NA	
	3.4 If Y/PY/NI to 3.3: Is it likely that missingness in the outcome depended on its true value?	NA	
	**Risk of bias judgement**	**Low**	
**Bias in measurement of the outcome**	4.1 Was the method of measuring the outcome inappropriate?	N	WOMAC、TUG、10MWT (ICC:0.83-0.99)
	4.2 Could measurement or ascertainment of the outcome have differed between intervention groups?	N	the same assessor performed measurements before and after intervention
	4.3 Were outcome assessors aware of the intervention received by study participants?	N	the assessors were completely blinded to which group the patients belonged to.
	4.4 If Y/PY/NI to 4.3: Could assessment of the outcome have been influenced by knowledge of intervention received?	NA	
	4.5 If Y/PY/NI to 4.4: Is it likely that assessment of the outcome was influenced by knowledge of intervention received?	NA	
	**Risk of bias judgement**	**Low**	
**Bias in selection of the reported result**	5.1 Were the data that produced this result analysed in accordance with a pre-specified analysis plan that was finalized before unblinded outcome data were available for analysis?	Y	This study was approved by the Sahmyook University Institutional Review Board [illegible]
	5.2 ... multiple eligible outcome measurements (e.g. scales, definitions, time points) within the outcome domain?	N	
	5.3 ... multiple eligible analyses of the data?	N	
	**Risk of bias judgement**	**Low**	
**Overall bias**	**Risk of bias judgement**	**Low**	

**Unique ID**	Steadman 2003	**Study ID**	Steadman 2003	**Assessor**	Chen
**Ref or Label**		**Aim**	assignment to intervention (the 'intention-to-treat' effect)		
**Experimental**	BT	**Comparator**	Conventional exercises	**Source**	Journal article(s); Trial protocol; Non-commercial trial registry record (e.g. ClinicalTrials.gov record)
**Outcome**	BBS 10m-WT	**Results**		**Weight**	1

**Domain**	**Signalling question**	**Response**	**Comments**
**Bias arising from the randomization process**	1.1 Was the allocation sequence random?	Y	Block randomization was undertaken in groups of 30 using computer-generated random numbers.<br>name and hospital number
	1.2 Was the allocation sequence concealed until participants were enrolled and assigned to interventions?	Y	
	1.3 Did baseline differences between intervention groups suggest a problem with the randomization process?	N	There were no differences in BBS, BPM scores, 10-meter TWT, and other baseline [illegible]
	**Risk of bias judgement**	**Low**	
**Bias due to deviations from intended interventions**	2.1.Were participants aware of their assigned intervention during the trial?	Y	Although the same therapist could offer both types of therapy, crossover of therapists was kept to a minimum.
	2.2.Were carers and people delivering the interventions aware of participants' assigned intervention during the trial?	Y	
	2.3. If Y/PY/NI to 2.1 or 2.2: Were there deviations from the intended intervention that arose because of the experimental context?	N	
	2.4 If Y/PY to 2.3: Were these deviations likely to have affected the outcome?	NA	
	2.5. If Y/PY/NI to 2.4: Were these deviations from intended intervention balanced between groups?	NA	
	2.6 Was an appropriate analysis used to estimate the effect of assignment to intervention?	Y	Data were analyzed on an intention to treat basis to evaluate the effectiveness
	2.7 If N/PN/NI to 2.6: Was there potential for a substantial impact (on the result) of the failure to analyse participants in the group to which they were randomized?	NA	
	**Risk of bias judgement**	**Low**	
**Bias due to missing outcome data**	3.1 Were data for this outcome available for all, or nearly all, participants randomized?	Y	dropped n=32; total n=198 (16.1%)
	3.2 If N/PN/NI to 3.1: Is there evidence that result was not biased by missing outcome data?	NA	
	3.3 If N/PN to 3.2: Could missingness in the outcome depend on its true value?	NA	
	3.4 If Y/PY/NI to 3.3: Is it likely that missingness in the outcome depended on its true value?	NA	
	**Risk of bias judgement**	**Low**	
**Bias in measurement of the outcome**	4.1 Was the method of measuring the outcome inappropriate?	N	BBS 10m-WT
	4.2 Could measurement or ascertainment of the outcome have differed between intervention groups?	N	JS
	4.3 Were outcome assessors aware of the intervention received by study participants?	N	
	4.4 If Y/PY/NI to 4.3: Could assessment of the outcome have been influenced by knowledge of intervention received?	NA	
	4.5 If Y/PY/NI to 4.4: Is it likely that assessment of the outcome was influenced by knowledge of intervention received?	NA	
	**Risk of bias judgement**	**Low**	
**Bias in selection of the reported result**	5.1 Were the data that produced this result analysed in accordance with a pre-specified analysis plan that was finalized before unblinded outcome data were available for analysis?	PY	The Bromley Local Research Ethics Committee approved the study.
	5.2 ... multiple eligible outcome measurements (e.g. scales, definitions, time points) within the outcome domain?	N	
	5.3 ... multiple eligible analyses of the data?	N	ANOVA
	**Risk of bias judgement**	**Low**	
**Overall bias**	**Risk of bias judgement**	**Low**	

**Unique ID**	Hernández-Guillén 2020	**Study ID**	Hernández-Guillén 2020	**Assessor**	Chen
**Ref or Label**		**Aim**	assignment to intervention (the 'intention-to-treat' effect)		
**Experimental**	BT	**Comparator**	Exercises	**Source**	Journal article(s); Trial protocol; Non-commercial trial registry record (e.g. ClinicalTrials.gov record)
**Outcome**	BBS	**Results**		**Weight**	1

Domain	Signalling question	Response	Comments
**Bias arising from the randomization process**	1.1 Was the allocation sequence random?	Y	Participants were allocated to either the control or experimental group based on the output of a random number generator program (Matlab®).
	1.2 Was the allocation sequence concealed until participants were enrolled and assigned to interventions?	Y	
	1.3 Did baseline differences between intervention groups suggest a problem with the randomization process?	N	No between-group baseline differences were observed (p > 0.05).
	**Risk of bias judgement**	**Low**	
**Bias due to deviations from intended interventions**	2.1.Were participants aware of their assigned intervention during the trial?	Y	Participants were not blinded as they had been provided with an information brochure explaining the possible interventions. a manual therapist with 17 years of
	2.2.Were carers and people delivering the interventions aware of participants' assigned intervention during the trial?	Y	
	2.3. If Y/PY/NI to 2.1 or 2.2: Were there deviations from the intended intervention that arose because of the experimental context?	N	The compliance with the intervention for the joint mobilization sessions in the experimental [illegible]
	2.4 If Y/PY to 2.3: Were these deviations likely to have affected the outcome?	NA	
	2.5. If Y/PY/NI to 2.4: Were these deviations from intended intervention balanced between groups?	NA	
	2.6 Was an appropriate analysis used to estimate the effect of assignment to intervention?	Y	All statistical analyses were carried out in accordance with the intention-to-treat principle [illegible]
	2.7 If N/PN/NI to 2.6: Was there potential for a substantial impact (on the result) of the failure to analyse participants in the group to which they were randomized?	NA	
	**Risk of bias judgement**	**Low**	
**Bias due to missing outcome data**	3.1 Were data for this outcome available for all, or nearly all, participants randomized?	PY	total n=14 ,dropped n=3
	3.2 If N/PN/NI to 3.1: Is there evidence that result was not biased by missing outcome data?	NA	
	3.3 If N/PN to 3.2: Could missingness in the outcome depend on its true value?	NA	
	3.4 If Y/PY/NI to 3.3: Is it likely that missingness in the outcome depended on its true value?	NA	
	**Risk of bias judgement**	**Low**	
**Bias in measurement of the outcome**	4.1 Was the method of measuring the outcome inappropriate?	N	BBS,Lafayette goniometer
	4.2 Could measurement or ascertainment of the outcome have differed between intervention groups?	N	
	4.3 Were outcome assessors aware of the intervention received by study participants?	N	A physiotherapist with 5 years of experience, also blinded, assessed the outcomes.
	4.4 If Y/PY/NI to 4.3: Could assessment of the outcome have been influenced by knowledge of intervention received?	NA	
	4.5 If Y/PY/NI to 4.4: Is it likely that assessment of the outcome was influenced by knowledge of intervention received?	NA	
	**Risk of bias judgement**	**Low**	
**Bias in selection of the reported result**	5.1 Were the data that produced this result analysed in accordance with a pre-specified analysis plan that was finalized before unblinded outcome data were available for analysis?	Y	The study design complied with the Declaration of Helsinki, [illegible]
	5.2 ... multiple eligible outcome measurements (e.g. scales, definitions, time points) within the outcome domain?	N	BBS
	5.3 ... multiple eligible analyses of the data?	N	
	**Risk of bias judgement**	**Low**	
**Overall bias**	**Risk of bias judgement**	**Low**	

**Unique ID**	Lee 2012	**Study ID**	Lee 2012	**Assessor**	Chen
**Ref or Label**		**Aim**	assignment to intervention (the 'intention-to-treat' effect)		
**Experimental**	BT	**Comparator**	No training program	**Source**	Journal article(s); Trial protocol; Non-commercial trial registry record (e.g. ClinicalTrials.gov record)
**Outcome**	TUG FRT OLST	**Results**		**Weight**	1

Domain	Signalling question	Response	Comments
**Bias arising from the randomization process**	1.1 Was the allocation sequence random?	NI	
	1.2 Was the allocation sequence concealed until participants were enrolled and assigned to interventions?	NI	
	1.3 Did baseline differences between intervention groups suggest a problem with the randomization process?	PN	
	**Risk of bias judgement**	**Some concerns**	
**Bias due to deviations from intended interventions**	2.1.Were participants aware of their assigned intervention during the trial?	Y	
	2.2.Were carers and people delivering the interventions aware of participants' assigned intervention during the trial?	Y	
	2.3. If Y/PY/NI to 2.1 or 2.2: Were there deviations from the intended intervention that arose because of the experimental context?	N	
	2.4 If Y/PY to 2.3: Were these deviations likely to have affected the outcome?	NA	
	2.5. If Y/PY/NI to 2.4: Were these deviations from intended intervention balanced between groups?	NA	
	2.6 Was an appropriate analysis used to estimate the effect of assignment to intervention?	PN	intention-to-treat（ITT）
	2.7 If N/PN/NI to 2.6: Was there potential for a substantial impact (on the result) of the failure to analyse participants in the group to which they were randomized?	Y	
	**Risk of bias judgement**	**High**	
**Bias due to missing outcome data**	3.1 Were data for this outcome available for all, or nearly all, participants randomized?	PY	
	3.2 If N/PN/NI to 3.1: Is there evidence that result was not biased by missing outcome data?	NA	
	3.3 If N/PN to 3.2: Could missingness in the outcome depend on its true value?	NA	
	3.4 If Y/PY/NI to 3.3: Is it likely that missingness in the outcome depended on its true value?	NA	
	**Risk of bias judgement**	**Low**	
**Bias in measurement of the outcome**	4.1 Was the method of measuring the outcome inappropriate?	N	TUG FRT OLST
	4.2 Could measurement or ascertainment of the outcome have differed between intervention groups?	PN	
	4.3 Were outcome assessors aware of the intervention received by study participants?	NI	
	4.4 If Y/PY/NI to 4.3: Could assessment of the outcome have been influenced by knowledge of intervention received?	PN	
	4.5 If Y/PY/NI to 4.4: Is it likely that assessment of the outcome was influenced by knowledge of intervention received?	NA	
	**Risk of bias judgement**	**Low**	
**Bias in selection of the reported result**	5.1 Were the data that produced this result analysed in accordance with a pre-specified analysis plan that was finalized before unblinded outcome data were available for analysis?	PY	
	5.2 ... multiple eligible outcome measurements (e.g. scales, definitions, time points) within the outcome domain?	PN	
	5.3 ... multiple eligible analyses of the data?	PN	
	**Risk of bias judgement**	**Low**	
**Overall bias**	**Risk of bias judgement**	**High**	

Unique ID	Hirase 2015	Study ID	Hirase 2015	Assessor	Chen
Ref or Label		Aim	assignment to intervention (the 'intention-to-treat' effect)		
Experimental	BT	Comparator	No training program	Source	Journal article(s); Trial protocol; Non-commercial trial registry record (e.g. ClinicalTrials.gov record)
Outcome	OLST CST TUG TST FES	Results		Weight	1

Domain	Signalling question	Response	Comments
Bias arising from the randomization process	1.1 Was the allocation sequence random?	Y	Participants were randomized into 3 groups using the sealed envelope method.
	1.2 Was the allocation sequence concealed until participants were enrolled and assigned to interventions?	PY	
	1.3 Did baseline differences between intervention groups suggest a problem with the randomization process?	N	There were no significant differences between the 3 groups in terms of age, sex, incidence of [illegible]
	Risk of bias judgement	Low	
Bias due to deviations from intended interventions	2.1.Were participants aware of their assigned intervention during the trial?	Y	The same physical therapists assessed the participants and also conducted the intervention programs.
	2.2.Were carers and people delivering the interventions aware of participants' assigned intervention during the trial?	Y	
	2.3. If Y/PY/NI to 2.1 or 2.2: Were there deviations from the intended intervention that arose because of the experimental context?	N	There were no significant differences between the 3 groups in terms of study withdrawal and [illegible]
	2.4 If Y/PY to 2.3: Were these deviations likely to have affected the outcome?	NA	
	2.5. If Y/PY/NI to 2.4: Were these deviations from intended intervention balanced between groups?	NA	
	2.6 Was an appropriate analysis used to estimate the effect of assignment to intervention?	Y	A 3 (group) × 5 (time) analysis of variancne Post hoc Bonferroni tests
	2.7 If N/PN/NI to 2.6: Was there potential for a substantial impact (on the result) of the failure to analyse participants in the group to which they were randomized?	NA	
	Risk of bias judgement	Low	
Bias due to missing outcome data	3.1 Were data for this outcome available for all, or nearly all, participants randomized?	Y	86 of 93 participants completed the 4-month intervention... foam rubber (n=29), stable [illegible]
	3.2 If N/PN/NI to 3.1: Is there evidence that result was not biased by missing outcome data?	NA	
	3.3 If N/PN to 3.2: Could missingness in the outcome depend on its true value?	NA	
	3.4 If Y/PY/NI to 3.3: Is it likely that missingness in the outcome depended on its true value?	NA	
	Risk of bias judgement	Low	
Bias in measurement of the outcome	4.1 Was the method of measuring the outcome inappropriate?	N	OLST CST TUG TST FES
	4.2 Could measurement or ascertainment of the outcome have differed between intervention groups?	PN	
	4.3 Were outcome assessors aware of the intervention received by study participants?	Y	The same physical therapists assessed the participants and also conducted the [illegible]
	4.4 If Y/PY/NI to 4.3: Could assessment of the outcome have been influenced by knowledge of intervention received?	PN	
	4.5 If Y/PY/NI to 4.4: Is it likely that assessment of the outcome was influenced by knowledge of intervention received?	NA	
	Risk of bias judgement	Low	
Bias in selection of the reported result	5.1 Were the data that produced this result analysed in accordance with a pre-specified analysis plan that was finalized before unblinded outcome data were available for analysis?	NI	
	5.2 ... multiple eligible outcome measurements (e.g. scales, definitions, time points) within the outcome domain?	N	
	5.3 ... multiple eligible analyses of the data?	PN	
	Risk of bias judgement	Some concerns	
Overall bias	Risk of bias judgement	Some concerns	

Unique ID	El-Khoury 2015	Study ID	El-Khoury 2015	Assessor	Chen
Ref or Label		Aim	assignment to intervention (the 'intention-to-treat' effect)		
Experimental	BT	Comparator	No training program	Source	Journal article(s); Trial protocol; Non-commercial trial registry record (e.g. ClinicalTrials.gov record)
Outcome	TUG 6m-WT FTSST OLST FES-I	Results		Weight	1

Domain	Signalling question	Response	Comments
Bias arising from the randomization process	1.1 Was the allocation sequence random?	Y	The randomisation lists were computer generated, based on randomly permuted blocks of varying size (2, 4 or 6, randomly sampled with equal probability).
	1.2 Was the allocation sequence concealed until participants were enrolled and assigned to interventions?	Y	
	1.3 Did baseline differences between intervention groups suggest a problem with the randomization process?	N	
	Risk of bias judgement	Low	
Bias due to deviations from intended interventions	2.1.Were participants aware of their assigned intervention during the trial?	Y	Intervention instructors were all regular employees of SIEL Bleu, specifically trained for the study.
	2.2.Were carers and people delivering the interventions aware of participants' assigned intervention during the trial?	Y	
	2.3. If Y/PY/NI to 2.1 or 2.2: Were there deviations from the intended intervention that arose because of the experimental context?	PN	A significant number of women in the intervention group never started the [illegible]
	2.4 If Y/PY to 2.3: Were these deviations likely to have affected the outcome?	NA	
	2.5. If Y/PY/NI to 2.4: Were these deviations from intended intervention balanced between groups?	NA	
	2.6 Was an appropriate analysis used to estimate the effect of assignment to intervention?	PY	Analysis was by intention to treat.
	2.7 If N/PN/NI to 2.6: Was there potential for a substantial impact (on the result) of the failure to analyse participants in the group to which they were randomized?	NA	
	Risk of bias judgement	Low	
Bias due to missing outcome data	3.1 Were data for this outcome available for all, or nearly all, participants randomized?	N	Data on falls were missing (incomplete) for 105 (14.9%) participants who either died or [illegible]
	3.2 If N/PN/NI to 3.1: Is there evidence that result was not biased by missing outcome data?	Y	The sensitivity analysis suggests that the risk of bias from attrition is probably low.
	3.3 If N/PN to 3.2: Could missingness in the outcome depend on its true value?	NA	
	3.4 If Y/PY/NI to 3.3: Is it likely that missingness in the outcome depended on its true value?	NA	
	Risk of bias judgement	Low	
Bias in measurement of the outcome	4.1 Was the method of measuring the outcome inappropriate?	N	calendar postcard + fall interview
	4.2 Could measurement or ascertainment of the outcome have differed between intervention groups?	N	
	4.3 Were outcome assessors aware of the intervention received by study participants?	N	Falls were classified by an expert geriatrician (BC) blinded to group assignment
	4.4 If Y/PY/NI to 4.3: Could assessment of the outcome have been influenced by knowledge of intervention received?	NA	
	4.5 If Y/PY/NI to 4.4: Is it likely that assessment of the outcome was influenced by knowledge of intervention received?	NA	
	Risk of bias judgement	Low	
Bias in selection of the reported result	5.1 Were the data that produced this result analysed in accordance with a pre-specified analysis plan that was finalized before unblinded outcome data were available for analysis?	PY	Trial registration ClinicalTrials.gov (NCT00545350).
	5.2 ... multiple eligible outcome measurements (e.g. scales, definitions, time points) within the outcome domain?	N	injurious falls (moderate+serious)
	5.3 ... multiple eligible analyses of the data?	N	Shared frailty model and Analysis was by intention to treat.
	Risk of bias judgement	Low	
Overall bias	Risk of bias judgement	Low	

**Unique ID**	Miko 2018	**Study ID**	Miko 2018	**Assessor**	Chen
**Ref or Label**		**Aim**	assignment to intervention (the 'intention-to-treat' effect)		
**Experimental**	BT	**Comparator**	Regular walking	**Source**	Journal article(s); Trial protocol; Non-commercial trial registry record (e.g. ClinicalTrials.gov record)
**Outcome**	TUG BBS RBT	**Results**		**Weight**	1

Domain	Signalling question	Response	Comments
**Bias arising from the randomization process**	1.1 Was the allocation sequence random?	Y	100 participants were randomly assigned to the intervention or control groups; randomization was performed based on the assigned number in the patient diary (using a
	1.2 Was the allocation sequence concealed until participants were enrolled and assigned to interventions?	Y	
	1.3 Did baseline differences between intervention groups suggest a problem with the randomization process?	N	between group Not significant
	**Risk of bias judgement**	**Low**	
**Bias due to deviations from intended interventions**	2.1.Were participants aware of their assigned intervention during the trial?	Y	Complete blinding was not possible, as physiotherapists leading exercise sessions were knowledgeable about the participants in the intervention group.
	2.2.Were carers and people delivering the interventions aware of participants' assigned intervention during the trial?	Y	
	2.3. If Y/PY/NI to 2.1 or 2.2: Were there deviations from the intended intervention that arose because of the experimental context?	N	Those participants who completed the programme had high adherence (over 80%)… [illegible]
	2.4 If Y/PY to 2.3: Were these deviations likely to have affected the outcome?	NA	
	2.5. If Y/PY/NI to 2.4: Were these deviations from intended intervention balanced between groups?	NA	
	2.6 Was an appropriate analysis used to estimate the effect of assignment to intervention?	Y	intention-to-treat
	2.7 If N/PN/NI to 2.6: Was there potential for a substantial impact (on the result) of the failure to analyse participants in the group to which they were randomized?	NA	
	**Risk of bias judgement**	**Low**	
**Bias due to missing outcome data**	3.1 Were data for this outcome available for all, or nearly all, participants randomized?	Y	Analysed (n = 49),Intervention Analysed (n = 48),Control (<5%)
	3.2 If N/PN/NI to 3.1: Is there evidence that result was not biased by missing outcome data?	NA	
	3.3 If N/PN to 3.2: Could missingness in the outcome depend on its true value?	NA	
	3.4 If Y/PY/NI to 3.3: Is it likely that missingness in the outcome depended on its true value?	NA	
	**Risk of bias judgement**	**Low**	
**Bias in measurement of the outcome**	4.1 Was the method of measuring the outcome inappropriate?	N	TUG BBS stabilometer ergometry
	4.2 Could measurement or ascertainment of the outcome have differed between intervention groups?	N	
	4.3 Were outcome assessors aware of the intervention received by study participants?	N	A physiotherapist blinded to the study groups assessed partici pants' balance in static and [illegible]
	4.4 If Y/PY/NI to 4.3: Could assessment of the outcome have been influenced by knowledge of intervention received?	NA	
	4.5 If Y/PY/NI to 4.4: Is it likely that assessment of the outcome was influenced by knowledge of intervention received?	NA	
	**Risk of bias judgement**	**Low**	
**Bias in selection of the reported result**	5.1 Were the data that produced this result analysed in accordance with a pre-specified analysis plan that was finalized before unblinded outcome data were available for analysis?	PY	
	5.2 ... multiple eligible outcome measurements (e.g. scales, definitions, time points) within the outcome domain?	N	
	5.3 ... multiple eligible analyses of the data?	N	T-test Mann-Whitney U
	**Risk of bias judgement**	**Low**	
**Overall bias**	**Risk of bias judgement**	**Low**	

**Unique ID**	Madureira 2007	**Study ID**	Madureira 2007	**Assessor**	Chen
**Ref or Label**		**Aim**	assignment to intervention (the 'intention-to-treat' effect)		
**Experimental**	BT	**Comparator**	No training program	**Source**	Journal article(s); Trial protocol; Non-commercial trial registry record (e.g. ClinicalTrials.gov record)
**Outcome**	BBS TUG	**Results**		**Weight**	1

Domain	Signalling question	Response	Comments
**Bias arising from the randomization process**	1.1 Was the allocation sequence random?	Y	The patients were randomized consecutively into two groups.
	1.2 Was the allocation sequence concealed until participants were enrolled and assigned to interventions?	PY	
	1.3 Did baseline differences between intervention groups suggest a problem with the randomization process?	N	The basal characteristics of the patients of both groups were similar… with no statistically [illegible]
	**Risk of bias judgement**	**Low**	
**Bias due to deviations from intended interventions**	2.1.Were participants aware of their assigned intervention during the trial?	Y	
	2.2.Were carers and people delivering the interventions aware of participants' assigned intervention during the trial?	Y	
	2.3. If Y/PY/NI to 2.1 or 2.2: Were there deviations from the intended intervention that arose because of the experimental context?	N	A high level of adherence was observed absences occurred with justifications
	2.4 If Y/PY to 2.3: Were these deviations likely to have affected the outcome?	NA	
	2.5. If Y/PY/NI to 2.4: Were these deviations from intended intervention balanced between groups?	NA	
	2.6 Was an appropriate analysis used to estimate the effect of assignment to intervention?	PN	Data analysis was realized on 60 patients six patients desisted
	2.7 If N/PN/NI to 2.6: Was there potential for a substantial impact (on the result) of the failure to analyse participants in the group to which they were randomized?	PN	
	**Risk of bias judgement**	**Some concerns**	
**Bias due to missing outcome data**	3.1 Were data for this outcome available for all, or nearly all, participants randomized?	PY	Data analysis was realized on 60 patients (30 Intervention, 30 Control) out of 66 randomized [illegible]
	3.2 If N/PN/NI to 3.1: Is there evidence that result was not biased by missing outcome data?	NA	
	3.3 If N/PN to 3.2: Could missingness in the outcome depend on its true value?	NA	
	3.4 If Y/PY/NI to 3.3: Is it likely that missingness in the outcome depended on its true value?	NA	
	**Risk of bias judgement**	**Low**	
**Bias in measurement of the outcome**	4.1 Was the method of measuring the outcome inappropriate?	N	BBS TUG
	4.2 Could measurement or ascertainment of the outcome have differed between intervention groups?	N	
	4.3 Were outcome assessors aware of the intervention received by study participants?	N	Static and dynamic balance and mobility were evaluated in all patients, before and at the end [illegible]
	4.4 If Y/PY/NI to 4.3: Could assessment of the outcome have been influenced by knowledge of intervention received?	NA	
	4.5 If Y/PY/NI to 4.4: Is it likely that assessment of the outcome was influenced by knowledge of intervention received?	NA	
	**Risk of bias judgement**	**Low**	
**Bias in selection of the reported result**	5.1 Were the data that produced this result analysed in accordance with a pre-specified analysis plan that was finalized before unblinded outcome data were available for analysis?	NI	
	5.2 ... multiple eligible outcome measurements (e.g. scales, definitions, time points) within the outcome domain?	N	BBS TUG
	5.3 ... multiple eligible analyses of the data?	N	
	**Risk of bias judgement**	**Some concerns**	
**Overall bias**	**Risk of bias judgement**	**Some concerns**	

**Unique ID**	Madureira 2010	**Study ID**	Madureira 2010	**Assessor**	Chen
**Ref or Label**		**Aim**	assignment to intervention (the 'intention-to-treat' effect)		
**Experimental**	BT	**Comparator**	No Training Program	**Source**	Journal article(s); Trial protocol; Non-commercial trial registry record (e.g. ClinicalTrials.gov record)
**Outcome**	BBS	**Results**		**Weight**	1

Domain	Signalling question	Response	Comments
**Bias arising from the randomization process**	1.1 Was the allocation sequence random?	Y	Patients were randomized into two groups ... via lottery method.
	1.2 Was the allocation sequence concealed until participants were enrolled and assigned to interventions?	PY	
	1.3 Did baseline differences between intervention groups suggest a problem with the randomization process?	N	The characteristics of the patients at onset of the study were similar between the two groups
	**Risk of bias judgement**	**Low**	
**Bias due to deviations from intended interventions**	2.1.Were participants aware of their assigned intervention during the trial?	Y	The group assignment was concealed from the assessor but not from the participants and trainers.supervised by an experienced physiotherapist
	2.2.Were carers and people delivering the interventions aware of participants' assigned intervention during the trial?	Y	
	2.3. If Y/PY/NI to 2.1 or 2.2: Were there deviations from the intended intervention that arose because of the experimental context?	N	exercise conducted by patients at home were not supervised
	2.4 If Y/PY to 2.3: Were these deviations likely to have affected the outcome?	NA	
	2.5. If Y/PY/NI to 2.4: Were these deviations from intended intervention balanced between groups?	NA	
	2.6 Was an appropriate analysis used to estimate the effect of assignment to intervention?	PN	
	2.7 If N/PN/NI to 2.6: Was there potential for a substantial impact (on the result) of the failure to analyse participants in the group to which they were randomized?	Y	
	**Risk of bias judgement**	**Some concerns**	
**Bias due to missing outcome data**	3.1 Were data for this outcome available for all, or nearly all, participants randomized?	Y	9.1% dropped
	3.2 If N/PN/NI to 3.1: Is there evidence that result was not biased by missing outcome data?	NA	
	3.3 If N/PN to 3.2: Could missingness in the outcome depend on its true value?	NA	
	3.4 If Y/PY/NI to 3.3: Is it likely that missingness in the outcome depended on its true value?	NA	
	**Risk of bias judgement**	**Low**	
**Bias in measurement of the outcome**	4.1 Was the method of measuring the outcome inappropriate?	N	BBS
	4.2 Could measurement or ascertainment of the outcome have differed between intervention groups?	N	
	4.3 Were outcome assessors aware of the intervention received by study participants?	N	assessed by two physiotherapists blinded to group assignment.
	4.4 If Y/PY/NI to 4.3: Could assessment of the outcome have been influenced by knowledge of intervention received?	NA	
	4.5 If Y/PY/NI to 4.4: Is it likely that assessment of the outcome was influenced by knowledge of intervention received?	NA	
	**Risk of bias judgement**	**Low**	
**Bias in selection of the reported result**	5.1 Were the data that produced this result analysed in accordance with a pre-specified analysis plan that was finalized before unblinded outcome data were available for analysis?	NI	
	5.2 ... multiple eligible outcome measurements (e.g. scales, definitions, time points) within the outcome domain?	N	BBS
	5.3 ... multiple eligible analyses of the data?	N	Mann-Whitney U
	**Risk of bias judgement**	**Some concerns**	
**Overall bias**	**Risk of bias judgement**	**Some concerns**	

**Unique ID**	Markovic 2015	**Study ID**	Markovic 2015	**Assessor**	Chen
**Ref or Label**		**Aim**	assignment to intervention (the 'intention-to-treat' effect)		
**Experimental**	BT	**Comparator**	PT	**Source**	Journal article(s); Trial protocol; Non-commercial trial registry record (e.g. ClinicalTrials.gov record)
**Outcome**	SB-COP	**Results**		**Weight**	1

Domain	Signalling question	Response	Comments
**Bias arising from the randomization process**	1.1 Was the allocation sequence random?	Y	The group allocation schedule was developed by a statistician using computer generated random numbers and the list was held off site by an independent person.
	1.2 Was the allocation sequence concealed until participants were enrolled and assigned to interventions?	Y	
	1.3 Did baseline differences between intervention groups suggest a problem with the randomization process?	N	baseline p>0.05
	**Risk of bias judgement**	**Low**	
**Bias due to deviations from intended interventions**	2.1.Were participants aware of their assigned intervention during the trial?	Y	Huber and Pilates
	2.2.Were carers and people delivering the interventions aware of participants' assigned intervention during the trial?	Y	
	2.3. If Y/PY/NI to 2.1 or 2.2: Were there deviations from the intended intervention that arose because of the experimental context?	N	Each training session was led by a trained specialist and supervised by the researchers. [illegible]
	2.4 If Y/PY to 2.3: Were these deviations likely to have affected the outcome?	NA	
	2.5. If Y/PY/NI to 2.4: Were these deviations from intended intervention balanced between groups?	NA	
	2.6 Was an appropriate analysis used to estimate the effect of assignment to intervention?	Y	
	2.7 If N/PN/NI to 2.6: Was there potential for a substantial impact (on the result) of the failure to analyse participants in the group to which they were randomized?	NA	
	**Risk of bias judgement**	**Low**	
**Bias due to missing outcome data**	3.1 Were data for this outcome available for all, or nearly all, participants randomized?	Y	Four of 34 participants… did not finish the experiment due to personal reasons.(88%)
	3.2 If N/PN/NI to 3.1: Is there evidence that result was not biased by missing outcome data?	NA	
	3.3 If N/PN to 3.2: Could missingness in the outcome depend on its true value?	NA	
	3.4 If Y/PY/NI to 3.3: Is it likely that missingness in the outcome depended on its true value?	NA	
	**Risk of bias judgement**	**Low**	
**Bias in measurement of the outcome**	4.1 Was the method of measuring the outcome inappropriate?	N	AMTI,CoP,Optojump
	4.2 Could measurement or ascertainment of the outcome have differed between intervention groups?	N	
	4.3 Were outcome assessors aware of the intervention received by study participants?	N	
	4.4 If Y/PY/NI to 4.3: Could assessment of the outcome have been influenced by knowledge of intervention received?	NA	
	4.5 If Y/PY/NI to 4.4: Is it likely that assessment of the outcome was influenced by knowledge of intervention received?	NA	
	**Risk of bias judgement**	**Low**	
**Bias in selection of the reported result**	5.1 Were the data that produced this result analysed in accordance with a pre-specified analysis plan that was finalized before unblinded outcome data were available for analysis?	PY	
	5.2 ... multiple eligible outcome measurements (e.g. scales, definitions, time points) within the outcome domain?	N	
	5.3 ... multiple eligible analyses of the data?	N	A two-way ANOVA Bonferroni post-hoc
	**Risk of bias judgement**	**Low**	
**Overall bias**	**Risk of bias judgement**	**Low**	

**Unique ID**	Wallén 2018	**Study ID**	Wallén 2018	**Assessor**	Chen
**Ref or Label**		**Aim**	assignment to intervention (the 'intention-to-treat' effect)		
**Experimental**	BT	**Comparator**	Conventional care	**Source**	Journal article(s); Trial protocol; Non-commercial trial registry record (e.g. ClinicalTrials.gov record)
**Outcome**	GT Mini-BESTest	**Results**		**Weight**	1

Domain	Signalling question	Response	Comments
**Bias arising from the randomization process**	1.1 Was the allocation sequence random?	Y	This study is a long-term follow-up of a randomized controlled trial the process of random allocation has been described elsewhere.
	1.2 Was the allocation sequence concealed until participants were enrolled and assigned to interventions?	PY	
	1.3 Did baseline differences between intervention groups suggest a problem with the randomization process?	N	There were no significant differences (P > .05) between the control and training group in [illegible]
	**Risk of bias judgement**	**Low**	
**Bias due to deviations from intended interventions**	2.1.Were participants aware of their assigned intervention during the trial?	Y	Each session was supervised by two physical therapists educated in the training program.
	2.2.Were carers and people delivering the interventions aware of participants' assigned intervention during the trial?	Y	
	2.3. If Y/PY/NI to 2.1 or 2.2: Were there deviations from the intended intervention that arose because of the experimental context?	N	
	2.4 If Y/PY to 2.3: Were these deviations likely to have affected the outcome?	NA	
	2.5. If Y/PY/NI to 2.4: Were these deviations from intended intervention balanced between groups?	NA	
	2.6 Was an appropriate analysis used to estimate the effect of assignment to intervention?	Y	An intention-to-treat protocol was applied, where missing data were substituted with [illegible]
	2.7 If N/PN/NI to 2.6: Was there potential for a substantial impact (on the result) of the failure to analyse participants in the group to which they were randomized?	NA	
	**Risk of bias judgement**	**Low**	
**Bias due to missing outcome data**	3.1 Were data for this outcome available for all, or nearly all, participants randomized?	Y	analyzed(N=100) dropped(n=9) (9%)
	3.2 If N/PN/NI to 3.1: Is there evidence that result was not biased by missing outcome data?	NA	
	3.3 If N/PN to 3.2: Could missingness in the outcome depend on its true value?	NA	
	3.4 If Y/PY/NI to 3.3: Is it likely that missingness in the outcome depended on its true value?	NA	
	**Risk of bias judgement**	**Low**	
**Bias in measurement of the outcome**	4.1 Was the method of measuring the outcome inappropriate?	N	GT Mini-BESTest
	4.2 Could measurement or ascertainment of the outcome have differed between intervention groups?	N	
	4.3 Were outcome assessors aware of the intervention received by study participants?	PY	Which group the participants belonged to was only masked to the test leaders at baseline [illegible]
	4.4 If Y/PY/NI to 4.3: Could assessment of the outcome have been influenced by knowledge of intervention received?	PN	
	4.5 If Y/PY/NI to 4.4: Is it likely that assessment of the outcome was influenced by knowledge of intervention received?	NA	
	**Risk of bias judgement**	**Low**	
**Bias in selection of the reported result**	5.1 Were the data that produced this result analysed in accordance with a pre-specified analysis plan that was finalized before unblinded outcome data were available for analysis?	Y	This study is a long-term follow-up of a rand omized controlled trial (trial registration [illegible]
	5.2 ... multiple eligible outcome measurements (e.g. scales, definitions, time points) within the outcome domain?	N	GT Mini-BESTest
	5.3 ... multiple eligible analyses of the data?	N	ITT ANOVA
	**Risk of bias judgement**	**Low**	
**Overall bias**	**Risk of bias judgement**	**Low**	

**Unique ID**	Bao 2018	**Study ID**	Bao 2018	**Assessor**	Chen
**Ref or Label**		**Aim**	assignment to intervention (the 'intention-to-treat' effect)		
**Experimental**	BT	**Comparator**	No training program	**Source**	Journal article(s); Trial protocol; Non-commercial trial registry record (e.g. ClinicalTrials.gov record)
**Outcome**	ABC SOT Mini-BESTest FTSST FSST FRT GT TUG	**Results**		**Weight**	1

Domain	Signalling question	Response	Comments
**Bias arising from the randomization process**	1.1 Was the allocation sequence random?	Y	The twelve participants were randomly assigned to the experimental group (EG) or control group (CG) The study team randomized the participant assignments by
	1.2 Was the allocation sequence concealed until participants were enrolled and assigned to interventions?	Y	
	1.3 Did baseline differences between intervention groups suggest a problem with the randomization process?	N	The two-tailed, independent samples t-test showed no significant differences for all [illegible]
	**Risk of bias judgement**	**Low**	
**Bias due to deviations from intended interventions**	2.1.Were participants aware of their assigned intervention during the trial?	Y	The EG received vibrotactile SA while the CG completed the training without vibrotactile SA. The treating physical therapist (different from the blinded assessor)and study team made
	2.2.Were carers and people delivering the interventions aware of participants' assigned intervention during the trial?	Y	
	2.3. If Y/PY/NI to 2.1 or 2.2: Were there deviations from the intended intervention that arose because of the experimental context?	N	All participants completed the training and the three CBTs without complaints, pains, falls, or [illegible]
	2.4 If Y/PY to 2.3: Were these deviations likely to have affected the outcome?	NA	
	2.5. If Y/PY/NI to 2.4: Were these deviations from intended intervention balanced between groups?	NA	
	2.6 Was an appropriate analysis used to estimate the effect of assignment to intervention?	Y	The effects of training with versus without SA on the clinical outcome measures were [illegible]
	2.7 If N/PN/NI to 2.6: Was there potential for a substantial impact (on the result) of the failure to analyse participants in the group to which they were randomized?	NA	
	**Risk of bias judgement**	**Low**	
**Bias due to missing outcome data**	3.1 Were data for this outcome available for all, or nearly all, participants randomized?	Y	All participants completed the training and the three CBTs without complaints, pains, falls, or [illegible]
	3.2 If N/PN/NI to 3.1: Is there evidence that result was not biased by missing outcome data?	NA	
	3.3 If N/PN to 3.2: Could missingness in the outcome depend on its true value?	NA	
	3.4 If Y/PY/NI to 3.3: Is it likely that missingness in the outcome depended on its true value?	NA	
	**Risk of bias judgement**	**Low**	
**Bias in measurement of the outcome**	4.1 Was the method of measuring the outcome inappropriate?	N	ABC SOT Mini-BESTest FTSST FSST FRT GT TUG
	4.2 Could measurement or ascertainment of the outcome have differed between intervention groups?	N	completed in the clinical setting by a physical therapist blinded to the participants' study [illegible]
	4.3 Were outcome assessors aware of the intervention received by study participants?	N	
	4.4 If Y/PY/NI to 4.3: Could assessment of the outcome have been influenced by knowledge of intervention received?	NA	
	4.5 If Y/PY/NI to 4.4: Is it likely that assessment of the outcome was influenced by knowledge of intervention received?	NA	
	**Risk of bias judgement**	**Low**	
**Bias in selection of the reported result**	5.1 Were the data that produced this result analysed in accordance with a pre-specified analysis plan that was finalized before unblinded outcome data were available for analysis?	PY	
	5.2 ... multiple eligible outcome measurements (e.g. scales, definitions, time points) within the outcome domain?	N	ABC SOT Mini-BESTest FTSST FSST FRT GT TUG
	5.3 ... multiple eligible analyses of the data?	N	T-test,MDC analysis
	**Risk of bias judgement**	**Low**	
**Overall bias**	**Risk of bias judgement**	**Low**	

**Unique ID**	BS 2012	**Study ID**	BS 2012	**Assessor**	Chen
**Ref or Label**		**Aim**	assignment to intervention (the 'intention-to-treat' effect)		
**Experimental**	BT	**Comparator**	BT	**Source**	Journal article(s); Trial protocol; Non-commercial trial registry record (e.g. ClinicalTrials.gov record)
**Outcome**	8UG OLST FRT COP	**Results**		**Weight**	1

Domain	Signalling question	Response	Comments
**Bias arising from the randomization process**	1.1 Was the allocation sequence random?	Y	Sixteen participants were randomly assigned to Tai Chi; Sixteen subjects were assigned to the SBEP; Sixteen people were randomly assigned to BYOGA
	1.2 Was the allocation sequence concealed until participants were enrolled and assigned to interventions?	PY	
	1.3 Did baseline differences between intervention groups suggest a problem with the randomization process?	PN	Only one of the baseline features was significant
	**Risk of bias judgement**	**Low**	
**Bias due to deviations from intended interventions**	2.1.Were participants aware of their assigned intervention during the trial?	Y	The class was taught by a Physical Therapist and graduate research assistants. A certified Tai Chi master taught the class. The yoga program was taught by two certified
	2.2.Were carers and people delivering the interventions aware of participants' assigned intervention during the trial?	Y	
	2.3. If Y/PY/NI to 2.1 or 2.2: Were there deviations from the intended intervention that arose because of the experimental context?	N	
	2.4 If Y/PY to 2.3: Were these deviations likely to have affected the outcome?	NA	
	2.5. If Y/PY/NI to 2.4: Were these deviations from intended intervention balanced between groups?	NA	
	2.6 Was an appropriate analysis used to estimate the effect of assignment to intervention?	Y	Data were analyzed using separate 3 (group) x 2 (time) repeated-measures ANOVA. For [illegible]
	2.7 If N/PN/NI to 2.6: Was there potential for a substantial impact (on the result) of the failure to analyse participants in the group to which they were randomized?	NA	
	**Risk of bias judgement**	**Low**	
**Bias due to missing outcome data**	3.1 Were data for this outcome available for all, or nearly all, participants randomized?	Y	Sixteen participants were randomly assigned to Tai Chi; eleven completed training
	3.2 If N/PN/NI to 3.1: Is there evidence that result was not biased by missing outcome data?	NA	
	3.3 If N/PN to 3.2: Could missingness in the outcome depend on its true value?	NA	
	3.4 If Y/PY/NI to 3.3: Is it likely that missingness in the outcome depended on its true value?	NA	
	**Risk of bias judgement**	**Low**	
**Bias in measurement of the outcome**	4.1 Was the method of measuring the outcome inappropriate?	N	8UG OLST FRT COP
	4.2 Could measurement or ascertainment of the outcome have differed between intervention groups?	N	All tests were conducted by the same testers at each time point. Tests were performed by [illegible]
	4.3 Were outcome assessors aware of the intervention received by study participants?	PN	
	4.4 If Y/PY/NI to 4.3: Could assessment of the outcome have been influenced by knowledge of intervention received?	NA	
	4.5 If Y/PY/NI to 4.4: Is it likely that assessment of the outcome was influenced by knowledge of intervention received?	NA	
	**Risk of bias judgement**	**Low**	
**Bias in selection of the reported result**	5.1 Were the data that produced this result analysed in accordance with a pre-specified analysis plan that was finalized before unblinded outcome data were available for analysis?	PY	
	5.2 ... multiple eligible outcome measurements (e.g. scales, definitions, time points) within the outcome domain?	N	8UG OLST FRT COP
	5.3 ... multiple eligible analyses of the data?	N	Data were analyzed using separate 3 (group) x 2 (time) repeated-measures ANOVA. For [illegible]
	**Risk of bias judgement**	**Low**	
**Overall bias**	**Risk of bias judgement**	**Low**	

**Unique ID**	SaNtos 2017	**Study ID**	SaNtos 2017	**Assessor**	Chen
**Ref or Label**		**Aim**	assignment to intervention (the 'intention-to-treat' effect)		
**Experimental**	BT	**Comparator**	RT	**Source**	Journal article(s); Trial protocol; Non-commercial trial registry record (e.g. ClinicalTrials.gov record)
**Outcome**	RBT OLST BESTest	**Results**		**Weight**	1

Domain	Signalling question	Response	Comments
**Bias arising from the randomization process**	1.1 Was the allocation sequence random?	Y	The PD individuals were selected using a random number table generator (www.random.org). and identical opaque sealed envelopes... An
	1.2 Was the allocation sequence concealed until participants were enrolled and assigned to interventions?	Y	
	1.3 Did baseline differences between intervention groups suggest a problem with the randomization process?	N	Both groups were homogeneous (p > 0.05) with regard to anthropometric and clinical [illegible]
	**Risk of bias judgement**	**Low**	
**Bias due to deviations from intended interventions**	2.1.Were participants aware of their assigned intervention during the trial?	Y	Due to the nature of the interventions it was not possible to blind the individuals with PD in regard to the two types of training. All physiotherapists engaged in the study...
	2.2.Were carers and people delivering the interventions aware of participants' assigned intervention during the trial?	Y	
	2.3. If Y/PY/NI to 2.1 or 2.2: Were there deviations from the intended intervention that arose because of the experimental context?	N	No adverse events or side effects associated with the exercise programs were reported
	2.4 If Y/PY to 2.3: Were these deviations likely to have affected the outcome?	NA	
	2.5. If Y/PY/NI to 2.4: Were these deviations from intended intervention balanced between groups?	NA	
	2.6 Was an appropriate analysis used to estimate the effect of assignment to intervention?	Y	All statistics were performed according to intention-to-treat analyses." [illegible]
	2.7 If N/PN/NI to 2.6: Was there potential for a substantial impact (on the result) of the failure to analyse participants in the group to which they were randomized?	NA	
	**Risk of bias judgement**	**Low**	
**Bias due to missing outcome data**	3.1 Were data for this outcome available for all, or nearly all, participants randomized?	Y	Analyzed in ITT analysis (n = 21 BT; n = 19 RT)
	3.2 If N/PN/NI to 3.1: Is there evidence that result was not biased by missing outcome data?	NA	
	3.3 If N/PN to 3.2: Could missingness in the outcome depend on its true value?	NA	
	3.4 If Y/PY/NI to 3.3: Is it likely that missingness in the outcome depended on its true value?	NA	
	**Risk of bias judgement**	**Low**	
**Bias in measurement of the outcome**	4.1 Was the method of measuring the outcome inappropriate?	N	force platform, COP,RBT OLST BESTest
	4.2 Could measurement or ascertainment of the outcome have differed between intervention groups?	N	A blinded assessor (physiotherapist and specialized in PD) assessed the balance [illegible]
	4.3 Were outcome assessors aware of the intervention received by study participants?	N	
	4.4 If Y/PY/NI to 4.3: Could assessment of the outcome have been influenced by knowledge of intervention received?	NA	
	4.5 If Y/PY/NI to 4.4: Is it likely that assessment of the outcome was influenced by knowledge of intervention received?	NA	
	**Risk of bias judgement**	**Low**	
**Bias in selection of the reported result**	5.1 Were the data that produced this result analysed in accordance with a pre-specified analysis plan that was finalized before unblinded outcome data were available for analysis?	Y	Conducted according to standards established by the CONSORT statement. [illegible]
	5.2 ... multiple eligible outcome measurements (e.g. scales, definitions, time points) within the outcome domain?	N	RBT OLST BESTest
	5.3 ... multiple eligible analyses of the data?	N	All statistics were performed according to intention-to-treat analyses." [illegible]
	**Risk of bias judgement**	**Low**	
**Overall bias**	**Risk of bias judgement**	**Low**	

**Unique ID**	Carter ND 2001	**Study ID**	Carter ND 2001	**Assessor**	Chen
**Ref or Label**		**Aim**	assignment to intervention (the 'intention-to-treat' effect)		
**Experimental**	BT	**Comparator**	No training program	**Source**	Journal article(s); Trial protocol; Non-commercial trial registry record (e.g. ClinicalTrials.gov record)
**Outcome**	SB DB TFE Run	**Results**		**Weight**	1

Domain	Signalling question	Response	Comments
**Bias arising from the randomization process**	1.1 Was the allocation sequence random?	Y	After baseline measurements, 93 women were randomised to exercise intervention (n = 45) or control (n = 48) groups.
	1.2 Was the allocation sequence concealed until participants were enrolled and assigned to interventions?	PY	
	1.3 Did baseline differences between intervention groups suggest a problem with the randomization process?	N	Exercise and control groups did not differ in baseline for age, height, and weight Although [illegible]
	**Risk of bias judgement**	**Low**	
**Bias due to deviations from intended interventions**	2.1.Were participants aware of their assigned intervention during the trial?	Y	All instructors are registered by the BC recreation and parks association and certified by the BC Women's Hospital Osteoporosis programme.
	2.2.Were carers and people delivering the interventions aware of participants' assigned intervention during the trial?	Y	
	2.3. If Y/PY/NI to 2.1 or 2.2: Were there deviations from the intended intervention that arose because of the experimental context?	N	Participants reported no injuries from either measurement or the exercise intervention. [illegible]
	2.4 If Y/PY to 2.3: Were these deviations likely to have affected the outcome?	NA	
	2.5. If Y/PY/NI to 2.4: Were these deviations from intended intervention balanced between groups?	NA	
	2.6 Was an appropriate analysis used to estimate the effect of assignment to intervention?	Y	Analysis of covariance was used to examine differences after controlling for confounding [illegible]
	2.7 If N/PN/NI to 2.6: Was there potential for a substantial impact (on the result) of the failure to analyse participants in the group to which they were randomized?	NA	
	**Risk of bias judgement**	**Low**	
**Bias due to missing outcome data**	3.1 Were data for this outcome available for all, or nearly all, participants randomized?	Y	Data reported are on the 79 women (39 control, 40 exercise) who were available for [illegible]
	3.2 If N/PN/NI to 3.1: Is there evidence that result was not biased by missing outcome data?	NA	
	3.3 If N/PN to 3.2: Could missingness in the outcome depend on its true value?	NA	
	3.4 If Y/PY/NI to 3.3: Is it likely that missingness in the outcome depended on its true value?	NA	
	**Risk of bias judgement**	**Low**	
**Bias in measurement of the outcome**	4.1 Was the method of measuring the outcome inappropriate?	N	Equitest (SOT) timed figure-of-eight test Lord's strain-gauge dynamometry (r = 0.92– [illegible]
	4.2 Could measurement or ascertainment of the outcome have differed between intervention groups?	N	An experienced neuroscientist clinician performed all static balance measurements.
	4.3 Were outcome assessors aware of the intervention received by study participants?	PN	
	4.4 If Y/PY/NI to 4.3: Could assessment of the outcome have been influenced by knowledge of intervention received?	NA	
	4.5 If Y/PY/NI to 4.4: Is it likely that assessment of the outcome was influenced by knowledge of intervention received?	NA	
	**Risk of bias judgement**	**Low**	
**Bias in selection of the reported result**	5.1 Were the data that produced this result analysed in accordance with a pre-specified analysis plan that was finalized before unblinded outcome data were available for analysis?	PY	
	5.2 ... multiple eligible outcome measurements (e.g. scales, definitions, time points) within the outcome domain?	PN	SB DB TFE Run
	5.3 ... multiple eligible analyses of the data?	N	Analysis of covariance was used to examine differences after controlling for confounding [illegible]
	**Risk of bias judgement**	**Low**	
**Overall bias**	**Risk of bias judgement**	**Low**	

**Unique ID**	Sadeghi H 2021	**Study ID**	Sadeghi H 2021	**Assessor**	Chen
**Ref or Label**		**Aim**	assignment to intervention (the 'intention-to-treat' effect)		
**Experimental**	BT	**Comparator**	Daily activities	**Source**	Journal article(s); Trial protocol; Non-commercial trial registry record (e.g. ClinicalTrials.gov record)
**Outcome**	OLST TST TUG 10MWT	**Results**		**Weight**	1

Domain	Signalling question	Response	Comments
**Bias arising from the randomization process**	1.1 Was the allocation sequence random?	Y	participants drew lots out of a hat and were randomly assigned into 4 parallel groups (BT, VR, MIX, CON).
	1.2 Was the allocation sequence concealed until participants were enrolled and assigned to interventions?	PY	
	1.3 Did baseline differences between intervention groups suggest a problem with the randomization process?	N	A 1-way analyses of variance were conducted on baseline scores across groups.
	**Risk of bias judgement**	**Low**	
**Bias due to deviations from intended interventions**	2.1.Were participants aware of their assigned intervention during the trial?	Y	The group exercise sessions were facilitated by 3 certified trainers and supervised by a researcher who was not involved in testing.
	2.2.Were carers and people delivering the interventions aware of participants' assigned intervention during the trial?	Y	
	2.3. If Y/PY/NI to 2.1 or 2.2: Were there deviations from the intended intervention that arose because of the experimental context?	N	All participants in the training groups attended at least 80% of their exercise classes. No [illegible]
	2.4 If Y/PY to 2.3: Were these deviations likely to have affected the outcome?	NA	
	2.5. If Y/PY/NI to 2.4: Were these deviations from intended intervention balanced between groups?	NA	
	2.6 Was an appropriate analysis used to estimate the effect of assignment to intervention?	Y	Separate 1-way analyses of covariance... were conducted... using baseline variables as [illegible]
	2.7 If N/PN/NI to 2.6: Was there potential for a substantial impact (on the result) of the failure to analyse participants in the group to which they were randomized?	NA	
	**Risk of bias judgement**	**Low**	
**Bias due to missing outcome data**	3.1 Were data for this outcome available for all, or nearly all, participants randomized?	Y	The final number of participants was 58 BT (n=14), VR (n=15), MIX (n=14), CON (n=15) [illegible]
	3.2 If N/PN/NI to 3.1: Is there evidence that result was not biased by missing outcome data?	NA	
	3.3 If N/PN to 3.2: Could missingness in the outcome depend on its true value?	NA	
	3.4 If Y/PY/NI to 3.3: Is it likely that missingness in the outcome depended on its true value?	NA	
	**Risk of bias judgement**	**Low**	
**Bias in measurement of the outcome**	4.1 Was the method of measuring the outcome inappropriate?	N	Biodex Isokinetic Dynamometer OLST TST TUG 10MWT
	4.2 Could measurement or ascertainment of the outcome have differed between intervention groups?	N	All researchers involved in testing were blinded to group allocation.
	4.3 Were outcome assessors aware of the intervention received by study participants?	N	
	4.4 If Y/PY/NI to 4.3: Could assessment of the outcome have been influenced by knowledge of intervention received?	NA	
	4.5 If Y/PY/NI to 4.4: Is it likely that assessment of the outcome was influenced by knowledge of intervention received?	NA	
	**Risk of bias judgement**	**Low**	
**Bias in selection of the reported result**	5.1 Were the data that produced this result analysed in accordance with a pre-specified analysis plan that was finalized before unblinded outcome data were available for analysis?	N	The hamstring and quadriceps muscles strength tests were added to the methods [illegible]
	5.2 ... multiple eligible outcome measurements (e.g. scales, definitions, time points) within the outcome domain?	N	OLST TST TUG 10MWT
	5.3 ... multiple eligible analyses of the data?	N	ANCOVA t-test Bonferroni η^2 and ω^2
	**Risk of bias judgement**	**Some concerns**	
**Overall bias**	**Risk of bias judgement**	**Some concerns**	

**Unique ID**	Sörlén 2021	**Study ID**	Sörlén 2021	**Assessor**	Chen
**Ref or Label**		**Aim**	assignment to intervention (the 'intention-to-treat' effect)		
**Experimental**	BT	**Comparator**	No training program	**Source**	Journal article(s); Trial protocol; Non-commercial trial registry record (e.g. ClinicalTrials.gov record)
**Outcome**	TUG FES FES-I	**Results**		**Weight**	1

Domain	Signalling question	Response	Comments
**Bias arising from the randomization process**	1.1 Was the allocation sequence random?	Y	A six-sided dice was used for allocation at a 1:1-ratio between exercise and control.
	1.2 Was the allocation sequence concealed until participants were enrolled and assigned to interventions?	PY	The random allocation sequence, enrollment of participants, and intervention assignments
	1.3 Did baseline differences between intervention groups suggest a problem with the randomization process?	PN	[illegible] TUG have little difference (and no statistical [illegible]
	**Risk of bias judgement**	**Low**	
**Bias due to deviations from intended interventions**	2.1.Were participants aware of their assigned intervention during the trial?	Y	The training was performed in groups under supervision by an Exercise Physiologist.
	2.2.Were carers and people delivering the interventions aware of participants' assigned intervention during the trial?	Y	
	2.3. If Y/PY/NI to 2.1 or 2.2: Were there deviations from the intended intervention that arose because of the experimental context?	N	[illegible] group sessions was 87% 32% were fully [illegible]
	2.4 If Y/PY to 2.3: Were these deviations likely to have affected the outcome?	NA	
	2.5. If Y/PY/NI to 2.4: Were these deviations from intended intervention balanced between groups?	NA	
	2.6 Was an appropriate analysis used to estimate the effect of assignment to intervention?	Y	[illegible] using ANCOVA with covariate-adjustments [illegible]
	2.7 If N/PN/NI to 2.6: Was there potential for a substantial impact (on the result) of the failure to analyse participants in the group to which they were randomized?	NA	
	**Risk of bias judgement**	**Low**	
**Bias due to missing outcome data**	3.1 Were data for this outcome available for all, or nearly all, participants randomized?	PY	total(n=65) dropped(n=14) (n=21.5%)
	3.2 If N/PN/NI to 3.1: Is there evidence that result was not biased by missing outcome data?	NA	
	3.3 If N/PN to 3.2: Could missingness in the outcome depend on its true value?	NA	
	3.4 If Y/PY/NI to 3.3: Is it likely that missingness in the outcome depended on its true value?	NA	
	**Risk of bias judgement**	**Low**	
**Bias in measurement of the outcome**	4.1 Was the method of measuring the outcome inappropriate?	N	TUG FES FES-I (ICC for PSEO = 0.60, PSEC = 0.94)
	4.2 Could measurement or ascertainment of the outcome have differed between intervention groups?	N	[illegible] author, unblinded after the 1st manuscript [illegible]
	4.3 Were outcome assessors aware of the intervention received by study participants?	N	The training was performed in groups under supervision by an Exercise Physiologist.
	4.4 If Y/PY/NI to 4.3: Could assessment of the outcome have been influenced by knowledge of intervention received?	NA	
	4.5 If Y/PY/NI to 4.4: Is it likely that assessment of the outcome was influenced by knowledge of intervention received?	NA	
	**Risk of bias judgement**	**Low**	
**Bias in selection of the reported result**	5.1 Were the data that produced this result analysed in accordance with a pre-specified analysis plan that was finalized before unblinded outcome data were available for analysis?	PY	[illegible] clinicaltrials.gov (NCT03227666), approved [illegible]
	5.2 ... multiple eligible outcome measurements (e.g. scales, definitions, time points) within the outcome domain?	N	TUG FES FES-I
	5.3 ... multiple eligible analyses of the data?	N	[illegible] using ANCOVA with covariate-adjustments [illegible]
	**Risk of bias judgement**	**Low**	
**Overall bias**	**Risk of bias judgement**	**Low**	

**Unique ID**	Donath 2016	**Study ID**	Donath 2016	**Assessor**	Chen
**Ref or Label**		**Aim**	assignment to intervention (the 'intention-to-treat' effect)		
**Experimental**	BT	**Comparator**	Regular daily activity	**Source**	Journal article(s); Trial protocol; Non-commercial trial registry record (e.g. ClinicalTrials.gov record)
**Outcome**	OLST YBT	**Results**	YBT	**Weight**	1

Domain	Signalling question	Response	Comments
**Bias arising from the randomization process**	1.1 Was the allocation sequence random?	Y	The study was designed as a 3-armed randomized controlled trial with 3 parallel groups.
	1.2 Was the allocation sequence concealed until participants were enrolled and assigned to interventions?	Y	The assignment to the groups was conducted
	1.3 Did baseline differences between intervention groups suggest a problem with the randomization process?	N	There was no difference in baseline between the three group
	**Risk of bias judgement**	**Low**	
**Bias due to deviations from intended interventions**	2.1.Were participants aware of their assigned intervention during the trial?	Y	we merely did not blind the training supervisor and the assessor
	2.2.Were carers and people delivering the interventions aware of participants' assigned intervention during the trial?	Y	
	2.3. If Y/PY/NI to 2.1 or 2.2: Were there deviations from the intended intervention that arose because of the experimental context?	PN	Attendance rate(PIL: 90%，BAL: 93%)
	2.4 If Y/PY to 2.3: Were these deviations likely to have affected the outcome?	NA	
	2.5. If Y/PY/NI to 2.4: Were these deviations from intended intervention balanced between groups?	NA	
	2.6 Was an appropriate analysis used to estimate the effect of assignment to intervention?	N	conducted an as-treated analysis instead of an intention-to-treat analysis
	2.7 If N/PN/NI to 2.6: Was there potential for a substantial impact (on the result) of the failure to analyse participants in the group to which they were randomized?	PN	
	**Risk of bias judgement**	**Some concerns**	
**Bias due to missing outcome data**	3.1 Were data for this outcome available for all, or nearly all, participants randomized?	PY	[illegible] subjects were tested after completing the [illegible]
	3.2 If N/PN/NI to 3.1: Is there evidence that result was not biased by missing outcome data?	NA	
	3.3 If N/PN to 3.2: Could missingness in the outcome depend on its true value?	NA	
	3.4 If Y/PY/NI to 3.3: Is it likely that missingness in the outcome depended on its true value?	NA	
	**Risk of bias judgement**	**Low**	
**Bias in measurement of the outcome**	4.1 Was the method of measuring the outcome inappropriate?	N	OLST YBT
	4.2 Could measurement or ascertainment of the outcome have differed between intervention groups?	N	only one experienced investigator performed the tests.
	4.3 Were outcome assessors aware of the intervention received by study participants?	Y	we merely did not blind the assessor
	4.4 If Y/PY/NI to 4.3: Could assessment of the outcome have been influenced by knowledge of intervention received?	N	Most of the outcomes were objective measures (such as COP displacement, test time, etc.), which were not influenced by subjective judgment.
	4.5 If Y/PY/NI to 4.4: Is it likely that assessment of the outcome was influenced by knowledge of intervention received?	NA	
	**Risk of bias judgement**	**Low**	
**Bias in selection of the reported result**	5.1 Were the data that produced this result analysed in accordance with a pre-specified analysis plan that was finalized before unblinded outcome data were available for analysis?	NI	
	5.2 ... multiple eligible outcome measurements (e.g. scales, definitions, time points) within the outcome domain?	N	OLST YBT
	5.3 ... multiple eligible analyses of the data?	N	conducted an as-treated analysis instead of an intention-to-treat analysis
	**Risk of bias judgement**	**Some concerns**	
**Overall bias**	**Risk of bias judgement**	**Some concerns**	

**Unique ID**	Allin 2016	**Study ID**	Allin 2016	**Assessor**	Chen
**Ref or Label**		**Aim**	assignment to intervention (the 'intention-to-treat' effect)		
**Experimental**	BT	**Comparator**	No training program	**Source**	Journal article(s); Trial protocol; Non-commercial trial registry record (e.g. ClinicalTrials.gov record)
**Outcome**	GT	**Results**		**Weight**	1

Domain	Signalling question	Response	Comments
**Bias arising from the randomization process**	1.1 Was the allocation sequence random?	Y	Participants were first assigned to either the PBT or control intervention using minimization to balance groups with respect to age, sex, and physical activity level.
	1.2 Was the allocation sequence concealed until participants were enrolled and assigned to interventions?	PY	
	1.3 Did baseline differences between intervention groups suggest a problem with the randomization process?	N	Participant characteristics did not differ between the three slip groups
	**Risk of bias judgement**	**Low**	
**Bias due to deviations from intended interventions**	2.1.Were participants aware of their assigned intervention during the trial?	Y	Neither the participants nor investigators were blinded to group assignment.
	2.2.Were carers and people delivering the interventions aware of participants' assigned intervention during the trial?	Y	
	2.3. If Y/PY/NI to 2.1 or 2.2: Were there deviations from the intended intervention that arose because of the experimental context?	N	All 34 participants completed all four sessions of their assigned intervention.
	2.4 If Y/PY to 2.3: Were these deviations likely to have affected the outcome?	NA	
	2.5. If Y/PY/NI to 2.4: Were these deviations from intended intervention balanced between groups?	NA	
	2.6 Was an appropriate analysis used to estimate the effect of assignment to intervention?	Y	Welch's analysis of variance pairwise comparisons Fisher's Exact test
	2.7 If N/PN/NI to 2.6: Was there potential for a substantial impact (on the result) of the failure to analyse participants in the group to which they were randomized?	NA	
	**Risk of bias judgement**	**Low**	
**Bias due to missing outcome data**	3.1 Were data for this outcome available for all, or nearly all, participants randomized?	Y	All 34 participants completed all four sessions of their assigned intervention, and all 34 [illegible]
	3.2 If N/PN/NI to 3.1: Is there evidence that result was not biased by missing outcome data?	NA	
	3.3 If N/PN to 3.2: Could missingness in the outcome depend on its true value?	NA	
	3.4 If Y/PY/NI to 3.3: Is it likely that missingness in the outcome depended on its true value?	NA	
	**Risk of bias judgement**	**Low**	
**Bias in measurement of the outcome**	4.1 Was the method of measuring the outcome inappropriate?	N	
	4.2 Could measurement or ascertainment of the outcome have differed between intervention groups?	N	All sessions took place in a research lab, and were administered by the same investigator
	4.3 Were outcome assessors aware of the intervention received by study participants?	Y	Neither the participants nor investigators were blinded to group assignment.
	4.4 If Y/PY/NI to 4.3: Could assessment of the outcome have been influenced by knowledge of intervention received?	PN	
	4.5 If Y/PY/NI to 4.4: Is it likely that assessment of the outcome was influenced by knowledge of intervention received?	NA	
	**Risk of bias judgement**	**Low**	
**Bias in selection of the reported result**	5.1 Were the data that produced this result analysed in accordance with a pre-specified analysis plan that was finalized before unblinded outcome data were available for analysis?	PY	
	5.2 ... multiple eligible outcome measurements (e.g. scales, definitions, time points) within the outcome domain?	N	GT
	5.3 ... multiple eligible analyses of the data?	N	Welch's analysis of variance pairwise comparisons Fisher's Exact test
	**Risk of bias judgement**	**Low**	
**Overall bias**	**Risk of bias judgement**	**Low**	
