# Supplementary material for: Effects of different neuromuscular training modalities on balance performance in older adults: a systematic review and network meta-analysis
Source: Front Physiol. 2025 Aug 8;16:1623908. doi: 10.3389/fphys.2025.1623908 (PMC12370742; doi:10.3389/fphys.2025.1623908)
Supplement: Supplementary file 1 [file DataSheet1.zip › Supplementary Materials/Table S1 The complete search strategy for the databases.docx]

**Table S1.** The complete search strategy for the databases

| **Database** | **Complete Search Strategy** |
| --- | --- |
| PubMed | ("Aged"[Mesh]) OR ((((((((old people[Title/Abstract]) OR (elders[Title/Abstract])) OR (senior[Title/Abstract])) OR (old adult[Title/Abstract])) OR (older people[Title/Abstract])) OR (older adults[Title/Abstract])) OR (geriatric[Title/Abstract])) OR (Elderly[Title/Abstract])) AND ("Postural Balance"[MeSH Terms] OR ("posture balance"[Title/Abstract] OR "balance posture"[Title/Abstract] OR "posture balances"[Title/Abstract] OR "posture equilibrium"[Title/Abstract] OR "equilibrium posture"[Title/Abstract] OR (("Postural"[All Fields] OR "posturally"[All Fields] OR "Posture"[MeSH Terms] OR "Posture"[All Fields] OR "postures"[All Fields] OR "postured"[All Fields] OR "posturing"[All Fields]) OR "Equilibriums"[Title/Abstract]) OR (("Postural"[All Fields] OR "posturally"[All Fields] OR "Posture"[MeSH Terms] OR "Posture"[All Fields] OR "postures"[All Fields] OR "postured"[All Fields] OR "posturing"[All Fields]) OR "Equilibriums"[Title/Abstract]) OR "equilibrium musculoskeletal"[Title/Abstract] OR "postural equilibrium"[Title/Abstract] OR "equilibrium postural"[Title/Abstract] OR "balance postural"[Title/Abstract] OR "postural control"[Title/Abstract] OR "control postural"[Title/Abstract] OR "postural controls"[Title/Abstract] OR "posture control"[Title/Abstract] OR "control posture"[Title/Abstract] OR "posture controls"[Title/Abstract])) AND (((((neuromuscular training[MeSH Terms]) ) OR (neuromuscular[Title/Abstract])) OR (proprioceptive training program[Title/Abstract])) OR (sensorimotor training program[Title/Abstract])) AND (((randomized controlled trail[Title/Abstract]) OR (randomized[Title/Abstract])) OR (placebo[Title/Abstract])) |
| Cochrane Library | #1 MeSH descriptor: [Postual Balance] explode all trees |
|  | #2 (Postural Balance):ti,ab,kw or(Balance, Posture):ti,ab,kw or(Posture Balances):ti,ab,kw or(Posture Equilibrium):ti,ab,kw or(Equilibrium, Posture):ti,ab,kw or(Posture Equilibriums):ti,ab,kw or(Musculoskeletal Equilibrium):ti,ab,kw or(Equilibrium, Musculoskeletal):ti,ab,kw or(Postural Equilibrium):ti,ab,kw or(Equilibrium, Postural):ti,ab,kw or(Balance, Postural):ti,ab,kw or(Postural Control):ti,ab,kw or(Control, Postural):ti,ab,kw or(Postural Controls):ti,ab,kw or(Posture Control):ti,ab,kw or(Control, Posture):ti,ab,kw or(Posture Controls):ti,ab,kw |
|  | #3 #1 OR #2 |
|  | #4 MeSH descriptor: [Aged] in all MeSH products |
|  | #5 (old people):ti,ab,kw or(elders):ti,ab,kw or(senior):ti,ab,kw or(old adult):ti,ab,kw or(older people):ti,ab,kw or(older adults):ti,ab,kw or(geriatric):ti,ab,kw or(Elderly):ti,ab,kw |
|  | #6 #4 OR #5 |
|  | #7 (neuromuscular training):ti,ab,kw or (neuromuscular):ti,ab,kw or (Neural muscular training):ti,ab,kw or (proprioceptive training program):ti,ab,kw or (Somatosensory Training Program):ti,ab,kw or (Kinesthetic Training Program):ti,ab,kw or (Functional Proprioceptive Training Program):ti,ab,kw or (Body Awareness Training Program):ti,ab,kw or (Balance and Coordination Training Program):ti,ab,kw or (Proprioception-Based Rehabilitation Program):ti,ab,kw or (sensorimotor training program):ti,ab,kw or (Multisensory Motor Training Program):ti,ab,kw or (Whole-Body Vibration Training):ti,ab,kw or (Nerve-muscle training):ti,ab,kw or (Neuro-muscular conditioning):ti,ab,kw or (Neural-muscular conditioning):ti,ab,kw or (erve-muscle coordination training):ti,ab,kw or (NMT):ti,ab,kw or (Neuromuscular Training Program):ti,ab,kw or (WBV):ti,ab,kw or (Whole-Body Vibration Therapy):ti,ab,kw or (Whole-Body Vibration Conditioning ):ti,ab,kw or (Vibration-Enhanced Training ):ti,ab,kw or (Balance Training):ti,ab,kw or (Balance Rehabilitation ):ti,ab,kw or (Balance Enhancement Program):ti,ab,kw or (Balance and Posture Training):ti,ab,kw or ( Balance Conditioning):ti,ab,kw or (Stability Training):ti,ab,kw or (Balance and Coordination Training):ti,ab,kw |
|  | #8 #3 AND #6 AND #7 |
| Embase | #1 'aged'/exp |
|  | #2 'old people':ab,ti or'elders':ab,ti or'senior':ab,ti or'old adult':ab,ti or'older people':ab,ti or'older adults':ab,ti or'geriatric':ab,ti or'Elderly':ab,ti or'geriatric':ab,ti |
|  | #3 'neuromuscular training':ab,ti or'neuromuscular':ab,ti or'Neural muscular training':ab,ti or'Nerve-muscle training':ab,ti or'Neural-muscular conditioning':ab,ti or'Neuro-muscular conditioning':ab,ti or'erve-muscle coordination training':ab,ti or'NMT':ab,ti or'Neuromuscular Training Program':ab,ti or'proprioceptive training program':ab,ti or'Somatosensory Training Program':ab,ti or'Kinesthetic Training Program':ab,ti or'Functional Proprioceptive Training Program':ab,ti or'Body Awareness Training Program':ab,ti or'Balance and Coordination Training Program':ab,ti or'sensorimotor training program':ab,ti or'Proprioception-Based Rehabilitation Program':ab,ti or'Multisensory Motor Training Program':ab,ti or'WBV':ab,ti or'Whole-Body Vibration Training':ab,ti or'Whole-Body Vibration Therapy':ab,ti or'Whole-Body Vibration Conditioning ':ab,ti or'Balance Training':ab,ti or'Vibration-Enhanced Training ':ab,ti or'Balance Rehabilitation ':ab,ti or'Balance Enhancement Program':ab,ti or'Balance and Posture Training':ab,ti or' Balance Conditioning':ab,ti or'Stability Training':ab,ti or'Balance and Coordination Training':ab,ti |
|  | #4 'balance':ab,ti or 'Postural Balance':ab,ti or 'Posture Balance':ab,ti or 'Balance, Posture':ab,ti or 'Posture Balances':ab,ti or 'Posture Equilibrium':ab,ti or 'Posture Equilibriums':ab,ti or 'Equilibrium, Posture':ab,ti or 'Musculoskeletal Equilibrium':ab,ti or 'Equilibrium, Musculoskeletal':ab,ti or 'Postural Equilibrium':ab,ti or 'Equilibrium, Postural':ab,ti or 'Balance, Postural':ab,ti or 'Postural Control':ab,ti or 'Control, Postural':ab,ti or 'Postural Controls':ab,ti or 'Posture Control':ab,ti or 'Posture Controls':ab,ti or 'Control, Posture':ab,ti or |
|  | #5 #1 OR #2 |
|  | #6 #3 AND #4 AND #5 |
| Web of Science | (((((((((TS=(Aged)) OR TS=(old people)) OR TS=(elders)) OR TS=(senior)) OR TS=(old adult)) OR TS=(older people)) OR TS=(older adults)) OR TS=(geriatric)) OR TS=(Elderly)) AND  ((((((((((((((((((((((((((((((TS=(neuromuscular training)) OR TS=(neuromuscular)) OR TS=(Neural muscular training)) OR TS=(Nerve-muscle training)) OR TS=(Neuro-muscular conditioning)) OR TS=(Neural-muscular conditioning)) OR TS=(erve-muscle coordination training)) OR TS=(NMT)) OR TS=(Neuromuscular Training Program)) OR TS=(proprioceptive training program)) OR TS=(Somatosensory Training Program)) OR TS=(Kinesthetic Training Program)) OR TS=(Functional Proprioceptive Training Program)) OR TS=(Body Awareness Training Program)) OR TS=(Balance and Coordination Training Program)) OR TS=(Proprioception-Based Rehabilitation Program)) OR TS=(sensorimotor training program)) OR TS=(Multisensory Motor Training Program)) OR TS=(WBV)) OR TS=(Whole-Body Vibration Training)) OR TS=(Whole-Body Vibration Therapy)) OR TS=(Whole-Body Vibration Conditioning )) OR TS=(Vibration-Enhanced Training )) OR TS=(Balance Training)) OR TS=(Balance Rehabilitation )) OR TS=(Balance Enhancement Program)) OR TS=(Balance and Posture Training)) OR TS=( Balance Conditioning)) OR TS=(Stability Training)) OR TS=(Balance and Coordination Training)) AND (((((((((((((((((TS=(Postural Balance)) OR TS=(Balance, Posture)) OR TS=(Posture Balances)) OR TS=(Posture Equilibrium)) OR TS=(Equilibrium, Posture)) OR TS=(Posture Equilibriums)) OR TS=(Musculoskeletal Equilibrium)) OR TS=(Equilibrium, Musculoskeletal)) OR TS=(Postural Equilibrium)) OR TS=(Equilibrium, Postural)) OR TS=(Balance, Postural)) OR TS=(Postural Control)) OR TS=(Control, Postural)) OR TS=(Postural Controls)) OR TS=(Posture Control)) OR TS=(Control, Posture)) OR TS=(Posture Controls)) NOT (SILOID==("PPRN")) AND  (((TS=(randomized controlled)) OR TS=(randomized)) OR TS=(placebo)) |
| EBSCOhost | S1 AB neuromuscular training OR AB Neural muscular training OR AB Nerve-muscle training OR AB NMT OR AB proprioceptive training program OR AB Stability Training OR AB Functional Proprioceptive Training Program OR AB Proprioception-Based Rehabilitation Program OR AB sensorimotor training program OR AB WBV OR AB Whole-Body Vibration Training OR AB Balance Training |
|  | S2 AB Postural Balance OR AB Balance, Posture OR AB Posture Balances OR AB Equilibrium, Posture OR AB Posture Equilibriums OR AB Musculoskeletal Equilibrium OR AB Equilibrium, Musculoskeletal OR AB Postural Equilibrium OR AB Balance, Postural OR AB Postural Control OR AB Postural Controls OR AB Posture Controls |
|  | S3 AB Aged OR AB old people OR AB elders OR AB senior OR AB old adult OR AB older people OR AB older adults OR AB geriatric OR AB Elderly |
|  | S4 S1 AND S2 AND S3 |
