# Supplementary material for: Effects of different neuromuscular training modalities on balance performance in older adults: a systematic review and network meta-analysis
Source: Front Physiol. 2025 Aug 8;16:1623908. doi: 10.3389/fphys.2025.1623908 (PMC12370742; doi:10.3389/fphys.2025.1623908)
Supplement: Supplementary file 1 [file DataSheet1.zip › Supplementary Materials/Table S2 Assessment of Loop Inconsistency in the Network Meta-Analysis.docx]

**Table S2.** Assessment of Loop Inconsistency in the Network Meta-Analysis

| Outcome | Loop | IF | seIF | z_value | p_value | CI_95% | Loop_Heterog_tau2 |
| --- | --- | --- | --- | --- | --- | --- | --- |
| TUGT | Control-WBVT-BT | 0.217 | 0.618 | 0.352 | 0.725 | (0.00,1.43) | 0.229 |
| WT | Control-NT-BT | 0.779 | 0.526 | 1.480 | 0.139 | (0.00,1.81) | 0.035 |
| WT | Control-WBVT-BT | 0.287 | 0.452 | 0.635 | 0.525 | (0.00,1.17) | 0.048 |
| BBS | Control-WBVT-BT | 1.576 | 2.218 | 0.711 | 0.477 | (0.00,5.92) | 1.601 |
