# Supplementary material for: Effects of different neuromuscular training modalities on balance performance in older adults: a systematic review and network meta-analysis
Source: Front Physiol. 2025 Aug 8;16:1623908. doi: 10.3389/fphys.2025.1623908 (PMC12370742; doi:10.3389/fphys.2025.1623908)
Supplement: Supplementary file 1 [file DataSheet1.zip › Supplementary Materials/Table S3 Egger’s Test for Publication Bias Across Outcome Measures.docx]

| **Table S3** Egger’s Test for Publication Bias Across Outcome Measures | | | | |  |
| --- | --- | --- | --- | --- | --- |
| Outcome | β₁ Estimate | SE of β₁ | z-value | p-value |  |
|  |  |  |  |  |  |
| TUGT | -1.83 | 1.023 | -1.79 | 0.0742 |  |
| WT | -1.87 | 2.631 | -0.71 | 0.4761 |  |
| BBS | -0.32 | 0.815 | -0.4 | 0.6908 |  |

Note: Egger’s test was conducted under a random-effects model (REML) to assess potential small-study effects. A significant p-value (typically p < 0.05) indicates possible publication bias. β₁ represents the regression intercept of standardized effect sizes against their standard errors.
