# Supplementary figures and images for: Effects of different neuromuscular training modalities on balance performance in older adults: a systematic review and network meta-analysis
Source: Front Physiol. 2025 Aug 8;16:1623908. doi: 10.3389/fphys.2025.1623908 (PMC12370742; doi:10.3389/fphys.2025.1623908)

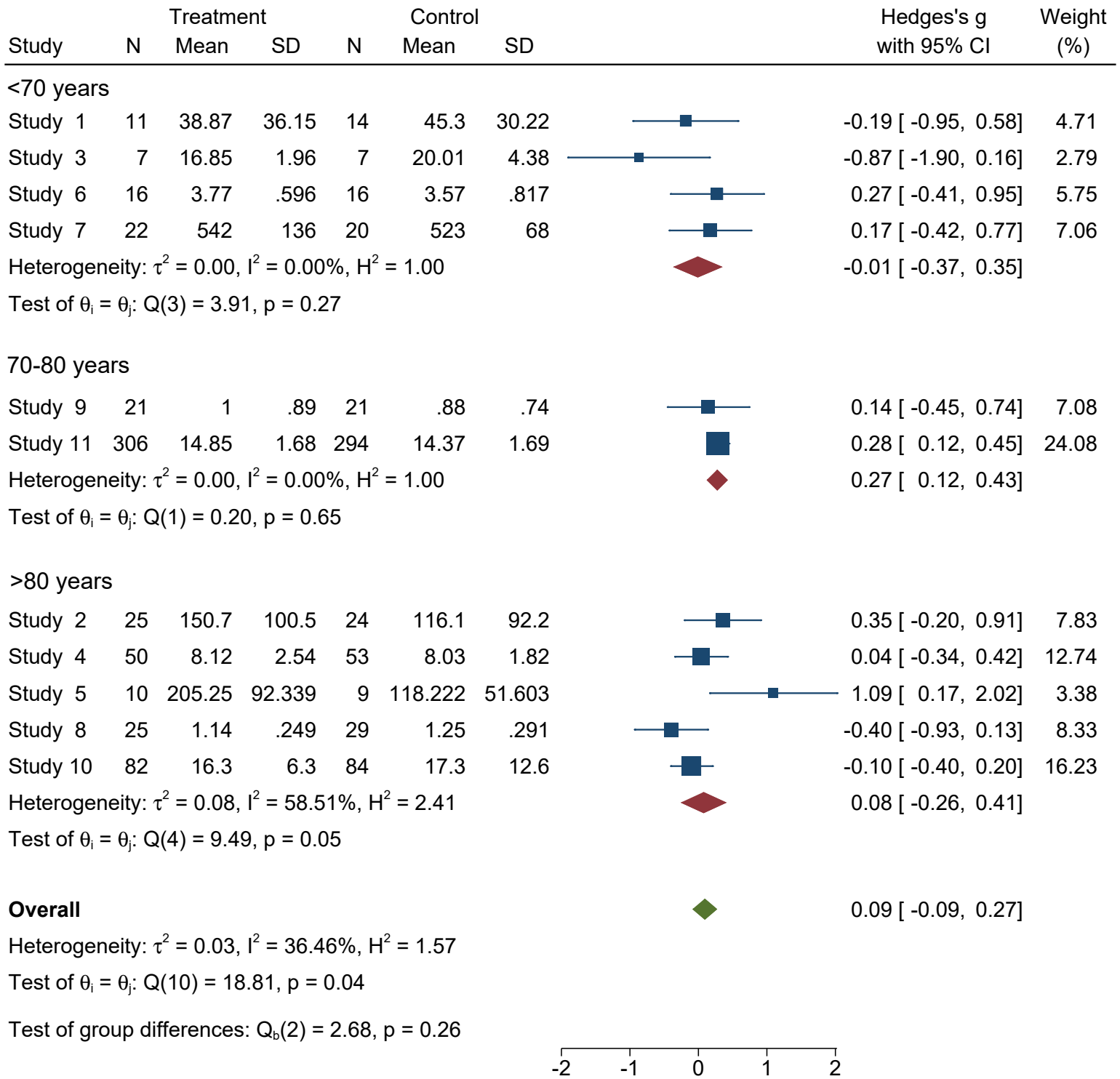

Supplement: Supplementary file 1 [file DataSheet1.zip › Supplementary Materials/Figure S10 Subgroup analysis of WT by age groups.pdf]

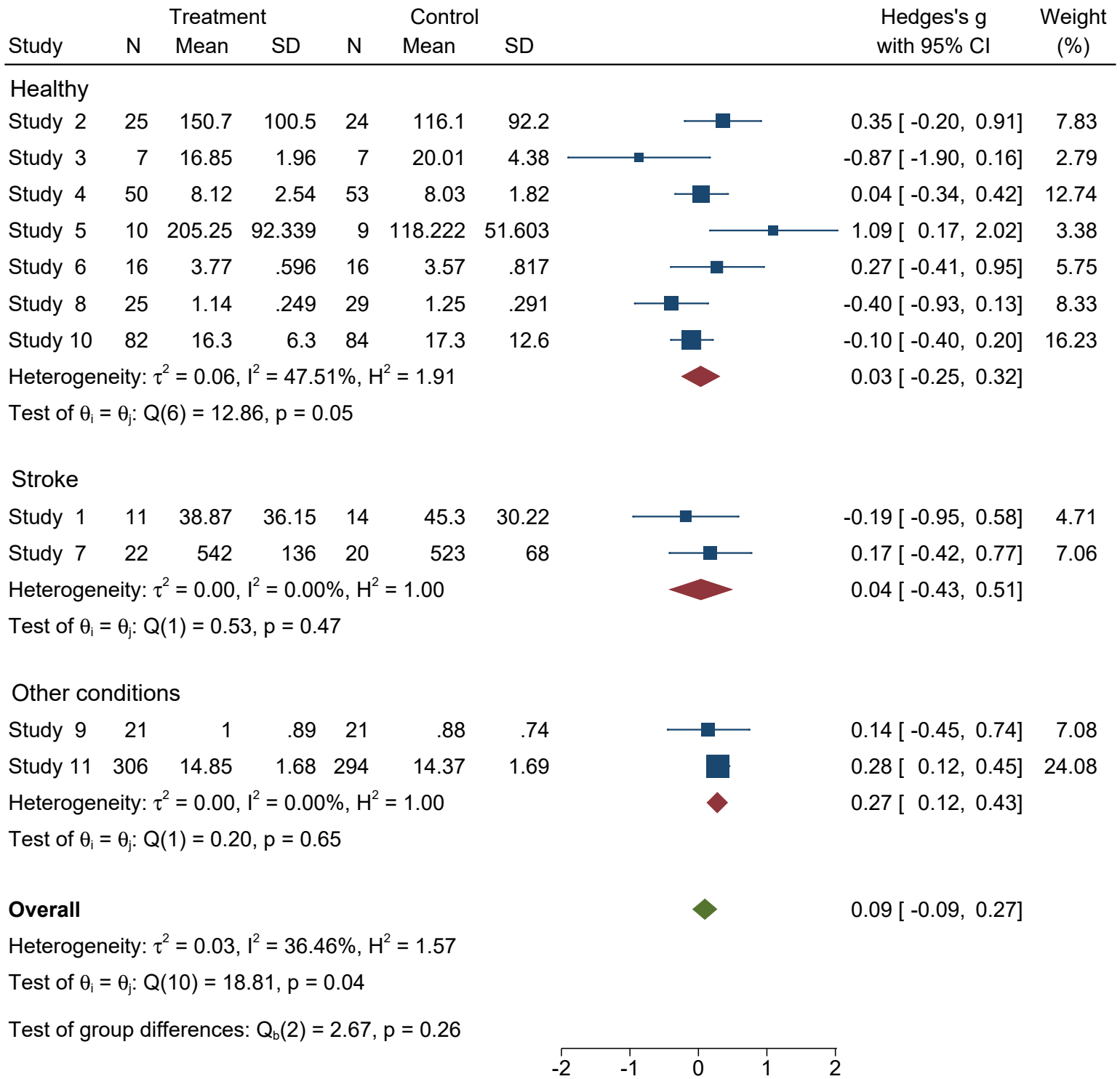

Supplement: Supplementary file 1 [file DataSheet1.zip › Supplementary Materials/Figure S11 Subgroup analysis of WT by health status.pdf]

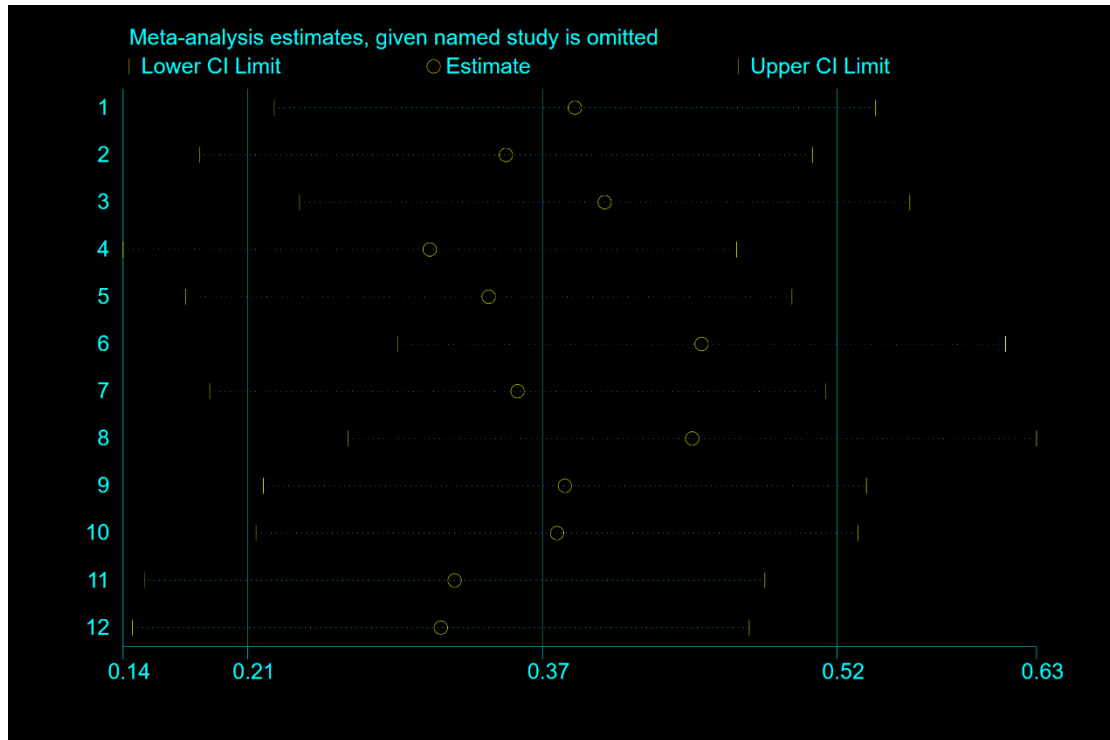

Supplement: Supplementary file 1 [file DataSheet1.zip › Supplementary Materials/Figure S12 Leave-one-out sensitivity analysis of BBS.pdf]

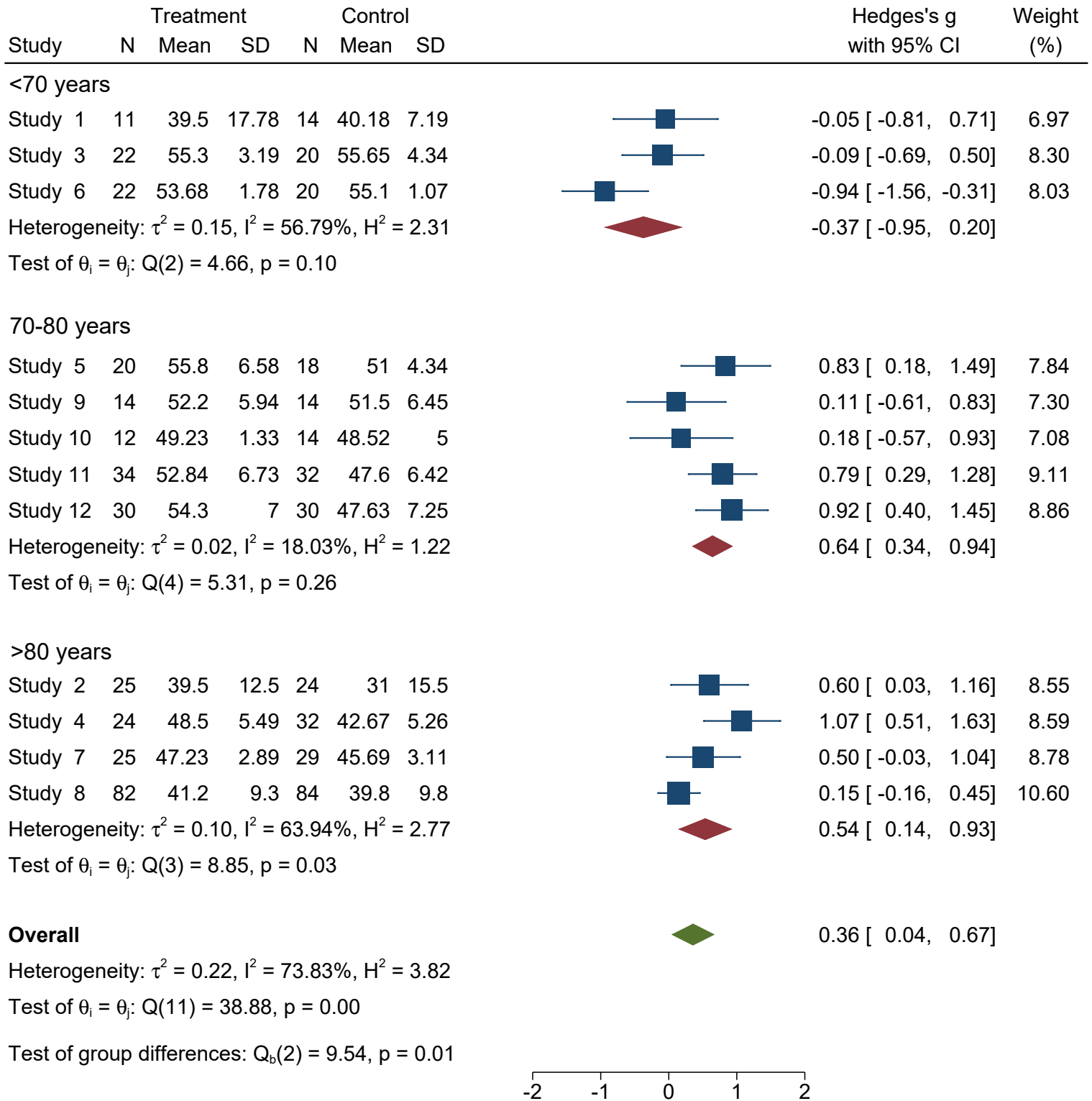

Supplement: Supplementary file 1 [file DataSheet1.zip › Supplementary Materials/Figure S13 Subgroup analysis of BBS by age groups.pdf]

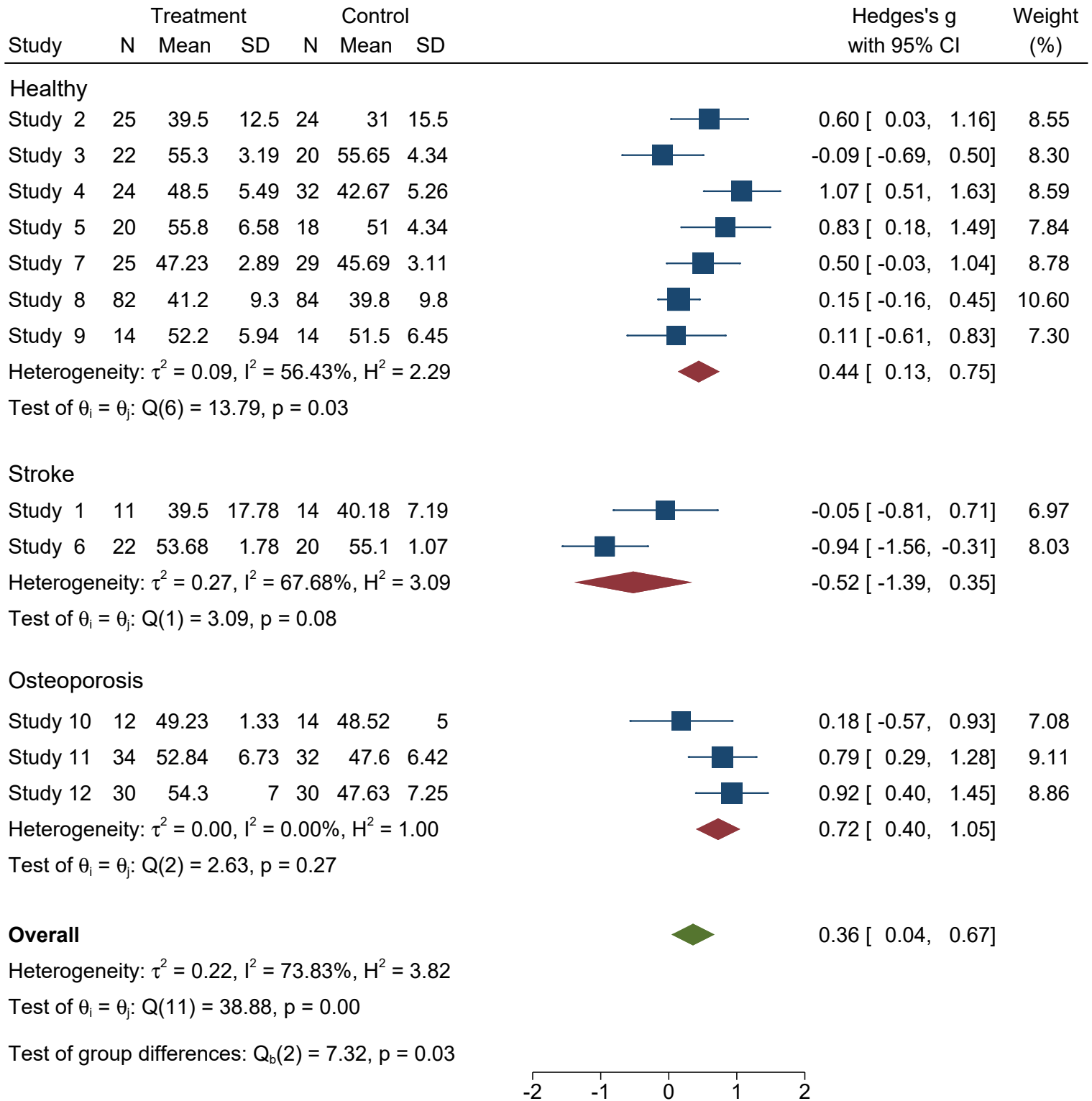

Random-effects REML model

Supplement: Supplementary file 1 [file DataSheet1.zip › Supplementary Materials/Figure S14 Subgroup analysis of BBS by health status.pdf]

**Treatment Effect****Mean with 95%CI and 95%PrI**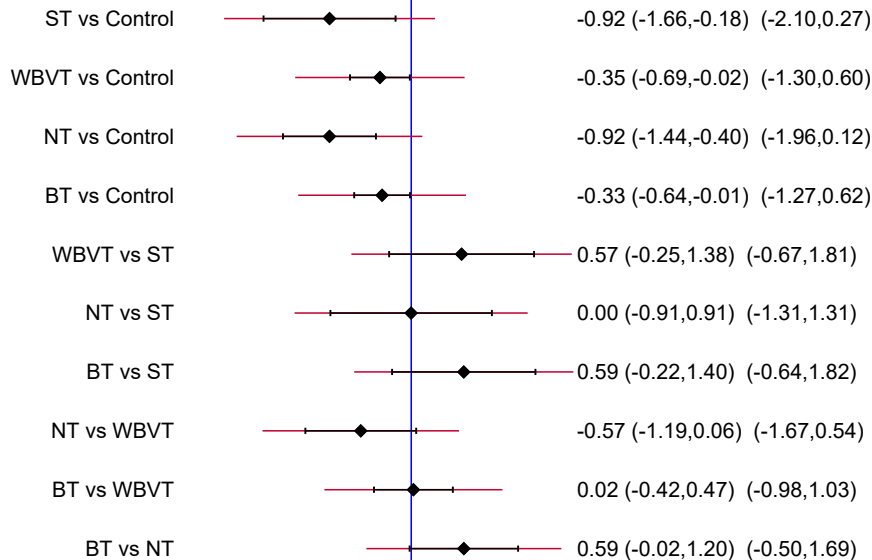

-2.1 -1.1 0 .8 1.8

Supplement: Supplementary file 1 [file DataSheet1.zip › Supplementary Materials/Figure S2 Predictive interval plot of TUGT.pdf]

# Treatment Effect

# Mean with 95%CI and 95%PrI

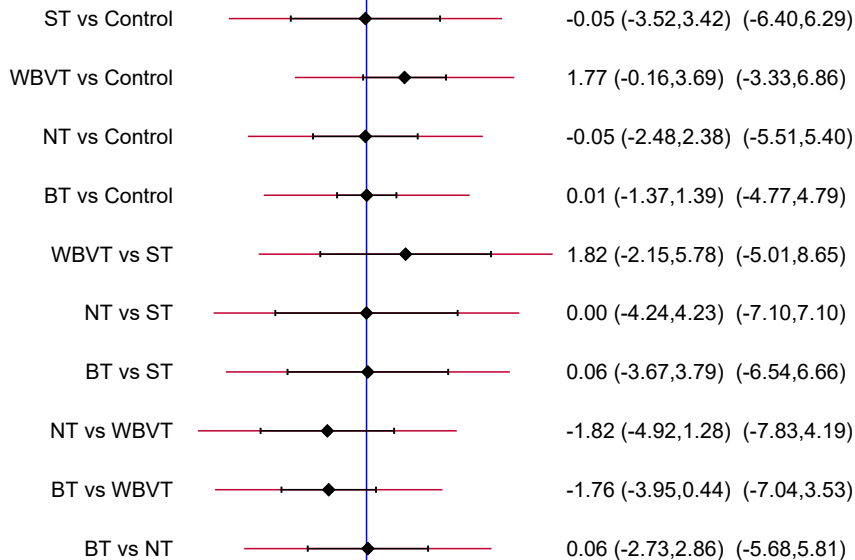

-7.8 -3.7 0 4.5 8.6

Supplement: Supplementary file 1 [file DataSheet1.zip › Supplementary Materials/Figure S4 Predictive interval plot of BBS.pdf]

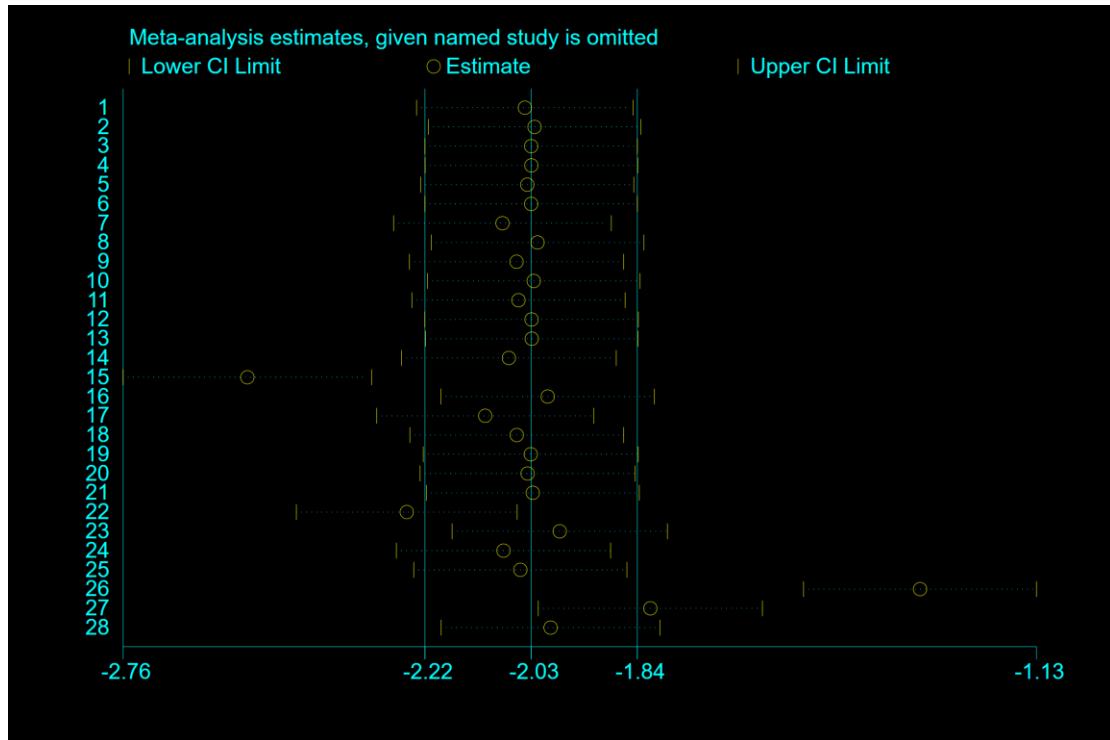

Supplement: Supplementary file 1 [file DataSheet1.zip › Supplementary Materials/Figure S5 Leave-one-out sensitivity analysis of TUGT before exclusion of high-risk studies.pdf]

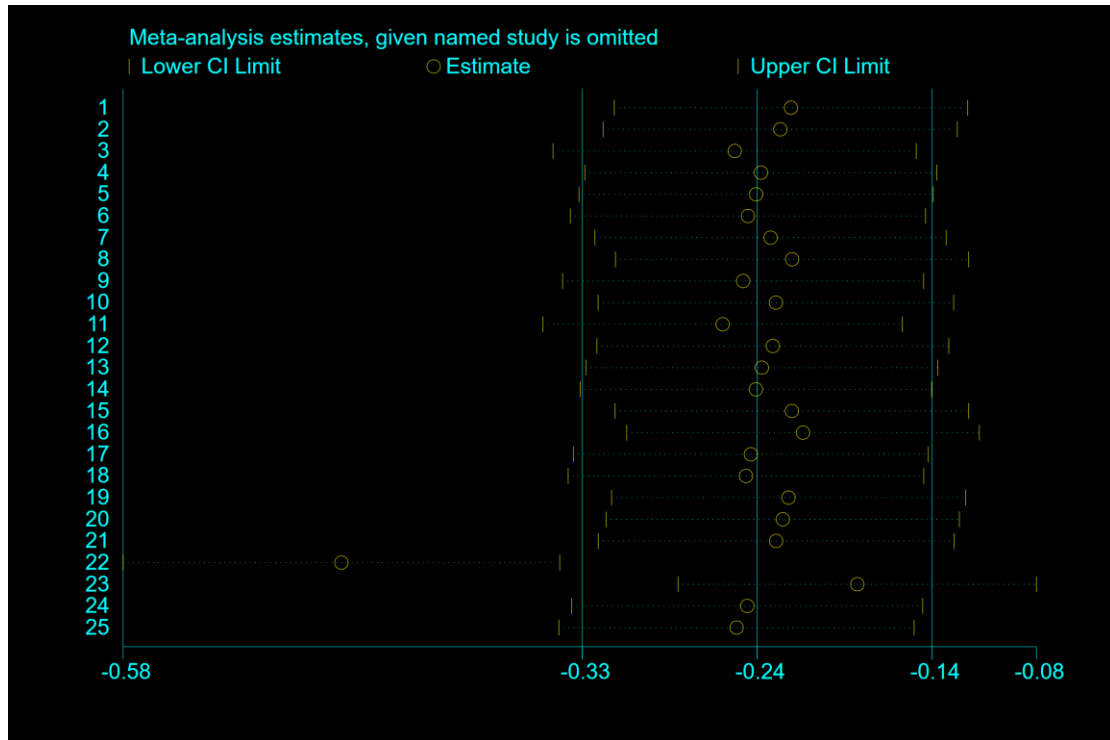

Supplement: Supplementary file 1 [file DataSheet1.zip › Supplementary Materials/Figure S6 Leave-one-out sensitivity analysis of TUGT after exclusion of high-risk studies.pdf]

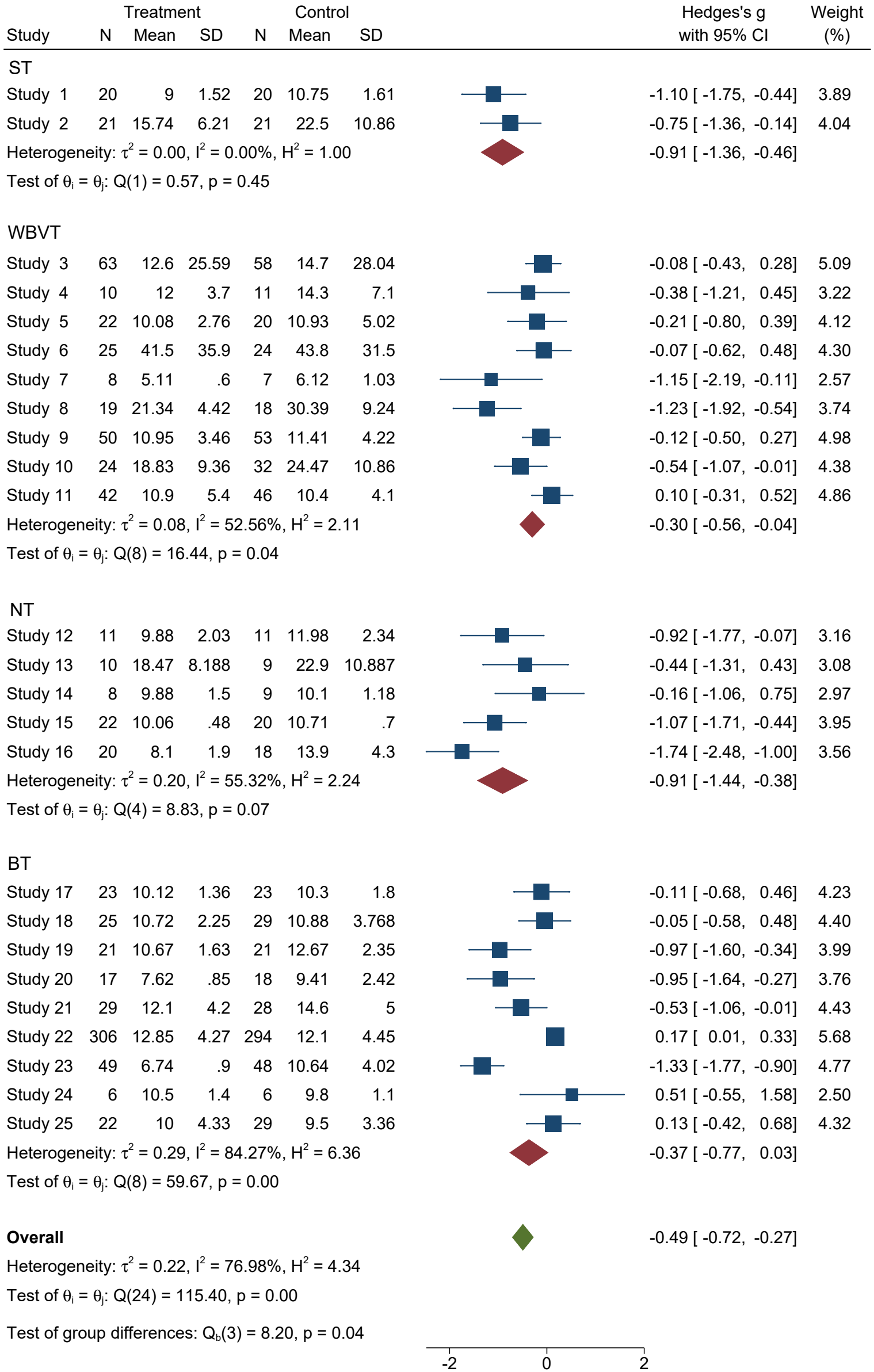

Supplement: Supplementary file 1 [file DataSheet1.zip › Supplementary Materials/Figure S7 Subgroup analysis of TUGT by training modality.pdf]

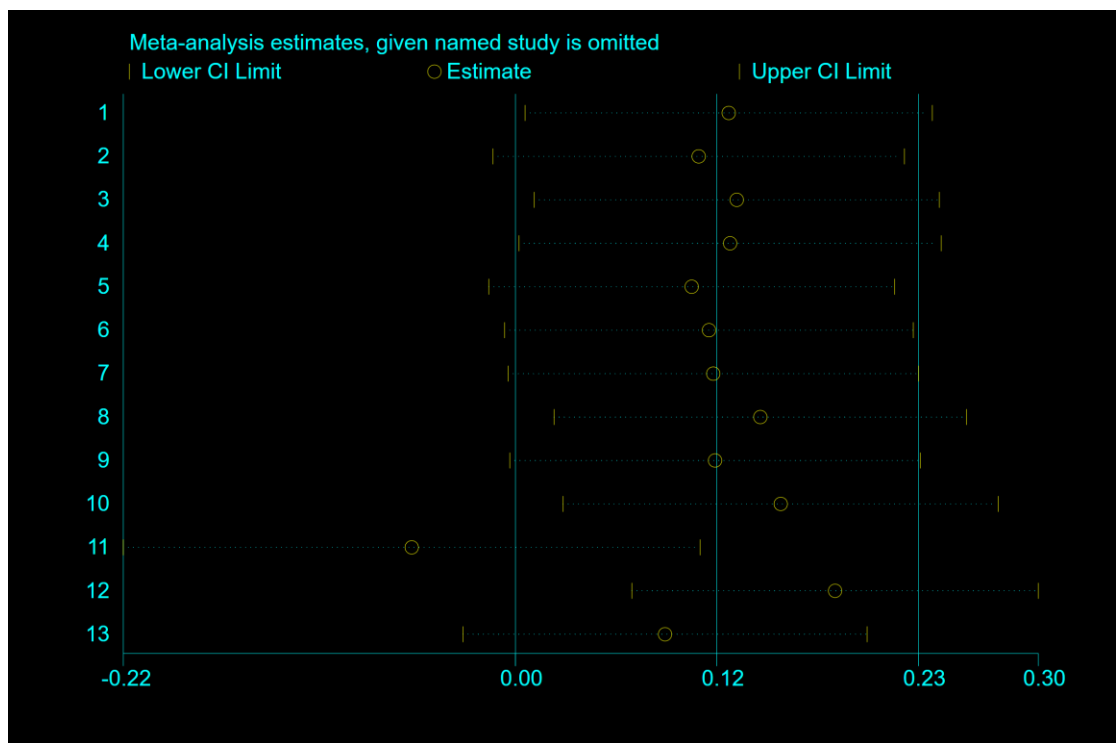

Supplement: Supplementary file 1 [file DataSheet1.zip › Supplementary Materials/Figure S8 Leave-one-out sensitivity analysis of WT before exclusion of high-risk studies.pdf]

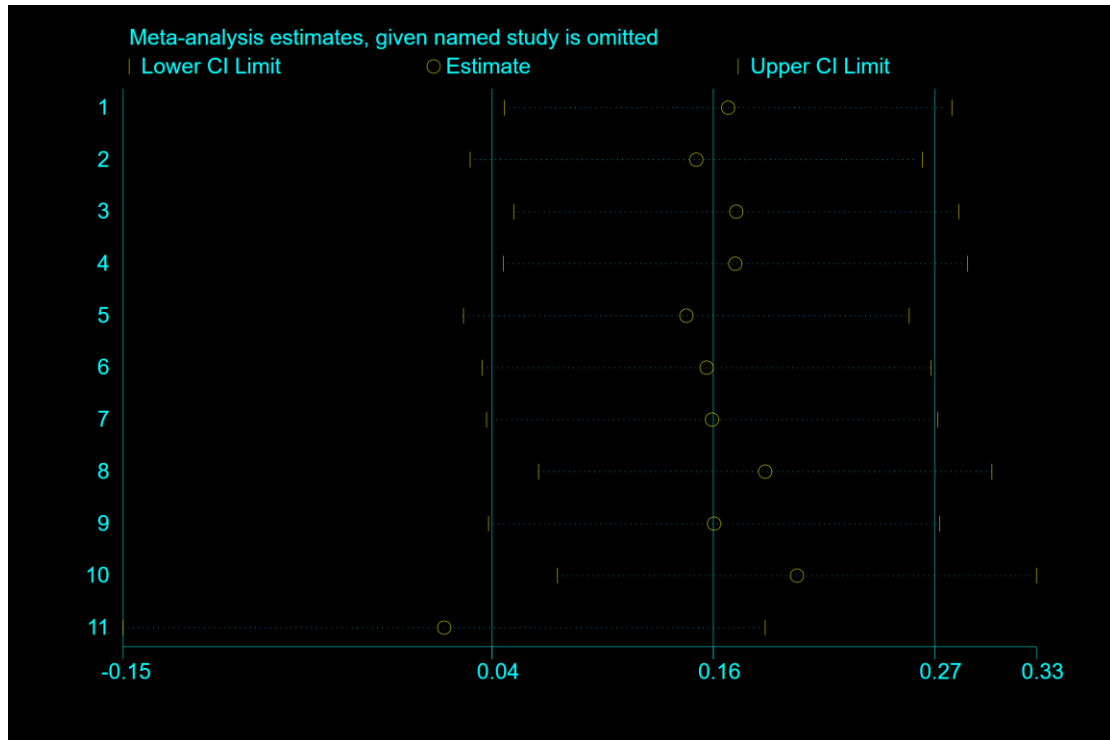

Supplement: Supplementary file 1 [file DataSheet1.zip › Supplementary Materials/Figure S9 Leave-one-out sensitivity analysis of WT after exclusion of high-risk studies.pdf]
